# Supplementary material for: Evaluating dengue burden in Africa in passive fever surveillance and seroprevalence studies: protocol of field studies of the Dengue Vaccine Initiative
Source: BMJ Open. 2018 Jan 21;8(1):e017673. doi: 10.1136/bmjopen-2017-017673 (PMC5780679; doi:10.1136/bmjopen-2017-017673)
Supplement: Author's manuscript [file bmjopen-2017-017673.draft_revisions.pdf]

BMJ Open

BMJ Open is committed to open peer review. As part of this commitment we make the peer review history of every article we publish publicly available.

When an article is published we post the peer reviewers' comments and the authors' responses online. We also post the versions of the paper that were used during peer review. These are the versions that the peer review comments apply to.

The versions of the paper that follow are the versions that were submitted during the peer review process. They are not the versions of record or the final published versions. They should not be cited or distributed as the published version of this manuscript.

BMJ Open is an open access journal and the full, final, typeset and author-corrected version of record of the manuscript is available on our site with no access controls, subscription charges or pay-per-view fees (http://bmjopen.bmj.com).

If you have any questions on BMJ Open's open peer review process please email editorial.bmjopen@bmj.com

BMJ Open

Evaluating dengue burden in Africa in passive fever surveillance and sero-prevalence studies: protocol of field studies of the Dengue Vaccine Initiative

Journal:	*BMJ Open*
Manuscript ID	bmjopen-2017-017673
Article Type:	Protocol
Date Submitted by the Author:	10-May-2017
Complete List of Authors:	Lim, Jacqueline; International Vaccine Institute, Global Dengue and Aedes-transmitted Diseases Consortium; London School of Hygiene and Tropical Medicine Faculty of Epidemiology and Population Health<br>Carabali, Mabel; International Vaccine Institute<br>Lee, Jung-Seok; International Vaccine Institute<br>Lee, Kang Sung ; International Vaccine Institute<br>Namkung, Suk; International Vaccine Institute<br>Lim, Sl-Ki; International Vaccine Institute<br>Ridde, Valéry; Université de Montréal, Centre de recherche du Centre hospitalier de l'Université de Montréal (CR-CHUM); Université de Montréal, Département de médecine sociale et préventive<br>Fernandes, Jose; Hopital Albert Schweitzer, Lambaréné,, Centre de Recherches Médicales de Lambaréné<br>Lell, Bertrand; Hopital Albert Schweitzer, Lambaréné, Centre de Recherches Médicales de Lambaréné<br>Matendechero, Sultani ; Ministry of Health, Kenya, Dept. of Neglected Tropical Diseases<br>Esen, Meral; University of Tübingen, Institute of Tropical Medicine<br>Andia, Esther; Kenya Medical Research Institute<br>Oyembo, Noah; Kenya Medical Research Institute<br>Barro, Ahmed; Action-Gouvernance-Integration-Reinforcement, Program Equité<br>Bonnet, Emmanuel ; Institut de recherche pour le developpement<br>Njenga, Sammy; Kenya Medical Research Institute, Eastern & Southern Africa Centre of International Parasite Control<br>Agnandji, Selidji ; Hopital Albert Schweitzer, Lambaréné, Centre de Recherches Médicales de Lambaréné<br>Yaro, Seydou; Centre MURAZ<br>Alexander, Neal; London School of Hygiene and Tropical Medicine Faculty of Epidemiology and Population Health<br>Yoon, In Kyu; International Vaccine Institute, Global Dengue and Aedes-transmitted Diseases Consortium
**Primary Subject Heading**:	Public health
Secondary Subject Heading:	Infectious diseases
Keywords:	dengue, Epidemiology < INFECTIOUS DISEASES, Public health < INFECTIOUS DISEASES, Tropical medicine < INFECTIOUS DISEASES

For peer review only

Evaluating dengue burden in Africa in passive fever surveillance and sero-prevalence studies: protocol of field studies of the Dengue Vaccine Initiative

Jacqueline K. Lim[1*], Mabel Carabali[1], Jung-Seok Lee[1], Kang Sung Lee[1], Suk Namkung[1], Sl-Ki Lim[1], Valery Ridde[2], Jose Fernandes[3], Bertrand Lell[3], Sultani Hadley Matendechero[4], Meral Esen[5], Esther Andia[6], Noah Oyembo[6], Ahmed Barro[7], Emmanuel Bonnet[8], Sammy M. Njenga[6], Selidji Todagbe Agnandji[3], Seydou Yaro[9], Neal Alexander[10], In-Kyu Yoon[1]

[1]International Vaccine Institute, Seoul, Korea, Republic of; [2]Montreal School of Public Health (ESPUM), Montreal, QC, Canada; [3]Centre de Recherches Médicales de Lambaréné, Hopital Albert Schweitzer, B.P.118, Lambaréné, Gabon; [4]Communicable Disease Prevention and Control, Ministry of Health, Nairobi, Kenya; [5]Institute of Tropical Medicine, University of Tübingen, Germany; [6]Eastern and Southern Africa Centre of International Parasite Control (ESACIPAC), Kenya Medical Research Institute (KEMRI), Nairobi, Kenya; [7]AGIR, Ouagadougou, Burkina Faso, [8]Institut de Recherche pour le Développement (IRD), Paris, France; [9]Centre Muraz, Bobo-Dioulasso, Burkina Faso; [10]Epidemiology and Public Health Department, London School of Hygiene and Tropical Medicine

*Corresponding author
Jacqueline K. Lim[1*], MPH
Research Scientist
Global Dengue and Aedes-transmitted Diseases Consortium (GDAC)
International Vaccine Institute (IVI)
SNU Research Park, Gwankak-ro 1
Gwanak-gu, Seoul, Korea 151-191
T: +82-2-881-1340 (Dir) / +82-2-872-2801 (ext. 340)
Email: kalim@ivi.int

Author information

Dr. Mabel Carabali, MD, MSc<br>Local medical epidemiologist<br>Dengue Vaccine Initiative<br>International Vaccine Institute (IVI)<br>Seoul, Korea<br>Email: mabel.carabali@gmail.com	Jung-Seok Lee, MSc, MPA<br>Health economist<br>International Vaccine Institute (IVI)<br>Seoul, Korea<br>Email: jslee@ivi.int
Kang Sung Lee, MSc<br>Researcher<br>International Vaccine Institute (IVI)<br>Seoul, Korea<br>Email: KangSungLee@ivi.int	Suk Namkung BSc<br>Program Research Assistant<br>International Vaccine Institute (IVI)<br>Seoul, Korea<br>Email: Suk.Namkung@ivi.int
Sl-Ki Lim, MPH<br>Researcher<br>International Vaccine Institute (IVI)<br>Seoul, Korea<br>Email: slkilim@ivi.int	Dr. Valery Ridde, PhD<br>Principal Scientist<br>Associate Professor, Department of Social and Preventive Medicine, École de santé publique, Université de Montréal<br>Email: valery.ridde@umontreal.ca

For peer review only - http://bmjopen.bmj.com/site/about/guidelines.xhtml

Jose Fernandes, MD<br>Physician Investigator<br>Centre de Recherches Médicales de Lambaréné, Hopital Albert Schweitzer, Lambaréné, Gabon<br>Email: jose.fernandes@lambarene.org	Bertrand Lell, MD, MSc<br>Co-director<br>Centre de Recherches Médicales de Lambaréné, Hopital Albert Schweitzer, Lambaréné, Gabon<br>Email: Bertrand.lell@lambarene.org
Sultani Hadley Matendechero, MD<br>Head of Neglected Tropical Diseases<br>Ministry of Health<br>Nairobi, Kenya<br>Email: hadleysultani@gmail.com	Meral Esen, MD<br>Group leader<br>Institute of Tropical Medicine<br>University of Tübingen<br>Germany,<br>Email: meral.esen@uni-turbingen.de
Esther Andia, BSc<br>Study coordinator<br>Kenya Medical Research Institute (KEMRI)<br>Nairobi, KENYA<br>Email: astar812@gmail.com	Noah Oyembo. BSc<br>Study coordinator<br>Kenya Medical Research Institute (KEMRI)<br>Nairobi, KENYA<br>Email: unouah@gmail.com
Ahmed Barro, BSc<br>Research Coordinator<br>Program Equité AGIR<br>Ouagadougou, Burkina Faso<br>Email: siebarro@gmail.com	Emmanuel Bonnet, PhD<br>Chargé de recherche<br>Institut de Recherche pour le Développement (IRD), Paris, France<br>Email: emmanuel.bonnet@ird.fr
Sammy M. Njenga, PhD<br>Director, Eastern and Southern Africa Centre of International Parasite Control (ESACIPAC)<br>Kenya Medical Research Institute (KEMRI)<br>Nairobi, KENYA<br>Email: SNjenga@kemri.org	Selidji Todagbe Agnandji, MD, PhD<br>Co-director<br>Centre de Recherches Médicales de Lambaréné, Hopital Albert Schweitzer, Lambaréné, Gabon<br>Email: agnandjis@cermel.org
Seydou Yaro, MD<br>Medical Doctor and Epidemiologist<br>Head of the Research Unit of Potential Epidemic Diseases<br>Centre MURAZ, Bobo-Dioulasso, Burkina Faso<br>Email: yaro_seydou@yahoo.com	Neal Alexander, PhD<br>Reader in Medical Statistics and Epidemiology<br>Epidemiology and Public Health Department<br>London School of Hygiene and Tropical Medicine<br>Email:Neal.Alexander@lshtm.ac.uk
In-Kyu Yoon, MD<br>Deputy Director General of Science<br>Director, Global Dengue and Aedes-transmitted Diseases Consortium (GDAC)<br>International Vaccine Institute (IVI)<br>Seoul, Korea 151-191<br>Email: InKyu.Yoon@ivi.int	

For peer review only

Abstract

Introduction.
Dengue is an important and well-documented public health problem in the Asia-Pacific and Latin American regions. However, in Africa, information on disease burden is limited to case reports and reports of sporadic outbreaks, thus hindering the implementation of public health actions for disease control.

Methods and Analysis.
In 2014-17, the Dengue Vaccine Initiative initiated field studies at three sites in Ouagadougou, Burkina Faso; Lambaréné, Gabon; and Mombasa, Kenya; to obtain comparable incidence data on dengue and assess its burden through standardized hospital-based surveillance and community-based serological methods. Multidisciplinary measurements of the burden of dengue were obtained through passive facility-based fever surveillance, cost-of-illness surveys, serological surveys, and healthcare utilization surveys. All three sites conducted case detection using standardized procedures with uniform laboratory assays to diagnose dengue. Healthcare utilization surveys were conducted to adjust population denominators in incidence calculations for differing healthcare seeking patterns. The fever surveillance data will allow calculation of age-specific incidence rates and comparison of symptomatic presentation between dengue and non-dengue patients using multivariable logistic regression. Serological surveys assessed changes in immune status of cohorts of 3,000 randomly selected residents at each site at 6 month intervals. The age-stratified serosurvey data will allow calculation of seroprevalence and force of infection of dengue. Cost-of-illness evaluations were conducted in a target sample size of 120-150 dengue-confirmed patients with acute dengue by Rapid Diagnostic patients.

Ethics and Dissemination.
The protocol for each study obtained ethical approvals from the Institutional Review Boards of the International Vaccine Institute, the London School of Hygiene and Tropical Medicine, and the Ethics Committee of host country institutions. By standardizing methods to evaluate dengue burden across several sites in Africa, these studies will generate evidence for dengue burden in Africa and data will be disseminated as publication in peer-review journals by the end of 2017.

Strengths of this study

- No study has been conducted with multi-disciplinary population-based approach to measure burden of dengue in multisites in Africa.
- This study will provide incidence of dengue in Africa from the surveillance and also provide prevalence and force of infection from the serological surveys.
- The manuscript provides details on how the studies were conducted in a standardized fashion across three sites with inherent site-specific differences.

Limitations of this study

- This was not a cohort study, so may under-estimate dengue disease burden by measuring incidence by conducting passive, facility-based, surveillance.
- There may be limited generalizability as this study is conducted in only 3 locations in Africa.

For peer review only

Word counts: 298 (abstract) and 6,557 (text)

Keywords: dengue; Africa; seroepidemiologic Studies; incidence

Running head: Evaluating dengue burden in Africa: DVI field studies

For peer review only

Background

Dengue fever, a mosquito-borne flavivirus infection caused by four related but antigenically distinct dengue viruses (DENVs, serotypes 1–4), is a major and rapidly increasing global public health problem. Recent studies have estimated an annual incidence of 50–100 million symptomatic infections globally [1]. Dengue is a high burden disease that disproportionately affects countries in the tropics and subtropics, many of which have limited health care resources [2]. Although one dengue vaccine has been recently licensed in several endemic countries, the vaccine has restricted age and epidemiological indications. Other prevention and control measures such as vector control are suboptimal as standalone interventions [3, 4], and no drugs for treatment are currently available.

The presence of *Aedes* mosquitoes and reports of dengue cases have been documented as early as 1823 in Africa [5]. However, most of these reports are limited to a few countries, and many cases have been from travellers, with a small number of reported autochthonous cases [6]. Amarasinghe et al. indicated that dengue cases have been reported in 34 countries in Africa, with most of these countries also having *Aedes* mosquitoes [6]. However, prior studies which suggested the presence of dengue were limited by their retrospective design or sample collection (blood donors or sample collected from surveys of other diseases) to demonstrate the true, population-based, burden of dengue. Also, while many dengue endemic countries in Asia and Latin America have mandatory reporting of dengue cases to public health authorities, and national surveillance systems in place to monitor incidence patterns [7], most African countries lack such established reporting mechanisms, and only sporadic outbreaks and individual case reports have been documented. In addition, the frequently non-specific clinical presentation of dengue may be difficult to distinguish from the myriad other infectious diseases present in Africa, since dengue diagnostic assays are not widely available.

For peer review

Thus, the burden of dengue remains largely unknown in Africa [6, 8]. Without such dengue burden data, informed decision-making about prevention and control measures, including dengue vaccine introduction, in Africa are not possible.

To improve estimates of population-based dengue disease burden in Africa, the Dengue Vaccine Initiative (DVI) initiated field studies at three sites in West, West-Central, and East Africa in Ouagadougou, Burkina Faso; Lambaréné, Gabon; and Mombasa, Kenya; respectively. In each of the three sites, a standardized package of study components, including passive facility-based fever surveillance, healthcare utilization surveys, cost-of-illness surveys, and serological surveys (Fig 1), was initiated between December, 2014 and March, 2016.

Methods

Site selection

Study sites were selected, in part, based on their likelihood of supporting DENV transmission. Dengue outbreaks and cases reported in the literature, available seroprevalence studies, and country-specific dengue risk maps of the probability of DENV transmission and the level of evidence of dengue burden from modelling were considered in site selection. [5] [9]. In addition, adequate research infrastructure to implement the studies was taken into account. Finally, inclusion of different regions of Africa was also a factor in site selection. Thus, Ouagadougou, Burkina Faso; Lambaréné, Gabon; and Mombasa, Kenya; were selected, respectively, to measure the true burden of dengue in West, (West-) Central, and East Africa.

In Burkina Faso, the first reported dengue outbreak occurred in Ouagadougou in 1982 due to DENV-2 [6]. Serological prevalence of dengue was found to be 26.3% in a rural setting (Nouna village) and 36.5% in an urban setting (Ouagadougou) in 2006 [10]. More

For peer review only - http://bmjopen.bmj.com/site/about/guidelines.xhtml

recently, an observational study conducted by Ridde et al. among febrile patients consulting at selected study facilities in 2013-14 showed 8.7 % (33/379) to be positive by dengue RDT; and 15 of 60 samples tested by RT-PCR to be dengue positive [11]. With evidence for the presence of dengue, along with a strong health and demographic surveillance system (Ouaga-HDSS) which could be used to describe the demographic characteristics of the catchment area, a field study was initiated in Ouagadougou, Burkina Faso in December 2014.

In Gabon, cases of dengue hemorrhagic fever (DHF) caused by up to three different DENV serotypes have been reported, and dengue seroprevalence has been found to be between 5 and 20% [12-14]. A recently published study results demonstrated seroprevalence of 12.3% among toddlers approximately 30 months of age in semi-rural Lambaréné between 2007 and 2010 [15]. However, a different study in 2005-2008 suggested minimal DENV transmission in rural areas of Gabon [16]. This latter study examined antibodies against dengue in individuals from randomly selected villages representing about 10% of all Gabonese villages. Blood samples were tested by anti-DENV IgG and IgM capture ELISA and found to have only minimal IgG (0.5%) and IgM (0.5%) seroprevalence. Based on these low prevalences, authors concluded that there was no active circulation of DENV in rural Gabon. However, the low seroprevalence may have been affected by low sensitivities of the tests used leading to a high rate of false negative, and/or selection bias in the blood sample pool among the selected villagers [17]. Seroprevalence estimates may have also been impacted by the possibility of false-positive results due to IgG cross-reactivity among flaviviruses [16]. Nevertheless, given the possibility of DENV circulation in Gabon, a field study was initiated in Lambaréné in March 2015 in a community with a catchment population of about 77,000 residents, using the clinical research infrastructure of the Centre de Recherches Medicales de Lambaréné (CERMEL), benefiting from experienced research staff who conducted a large Phase 3 malaria vaccine trial [18, 19].

For peer review only

In Kenya, more evidence is available for the presence of dengue based on local data. Dengue was the most common viral pathogen in retrospectively tested blood specimens from HIV-negative survey samples from the 2007 Kenya AIDS Indicator Survey. Antibody testing for dengue as well as chikungunya and Rift Valley fever was performed by IgG ELISA using either commercial kits or CDC assays; 12.5% were found to be dengue positive [20]. Similarly, a household survey found 13% of individuals from 701 households in Mombasa had serological evidence of either past or current DENV infection [21]. These data suggest that there is more dengue in Kenya than indicated by public health reporting, possibly due to misdiagnosis [20, 21]. A field study was initiated in Mombasa, Kenya in March 2016.

Study participants

For the passive facility-based fever surveillance, individuals who met the following criteria were eligible for study enrollment:

1. Age 1- 55 years old;
2. Resident of the catchment area covered by healthcare facilities participating in the study, without plans to move out of the catchment area within 12 months;
3. Signed informed consent, and assent for those aged between 7 and 18 years; and
4. Patients presenting with current fever (axillary temperature ≥ 37.5º C) or history of fever for ≤ 7 days duration without localizing signs (fever caused by a localized infection as well as fever with a known and confirmed etiology other than dengue, such as malaria confirmed by malaria RDT- listed in the patient identification SOP).

For the serological survey, criteria 1-3 were applied. For the healthcare utilization survey, household interviews were conducted among the heads or representatives of the household invited from each family participating in the serosurvey.

For peer review only - http://bmjopen.bmj.com/site/about/guidelines.xhtml

Study area and population

Burkina Faso, located in West Africa, has a population of 14,017,462 with 22.7% living in urban areas. The country is mainly rural with about 29% of the population reported to be living in urban areas in 2014. However, Burkina Faso is urbanizing rapidly and is positioned at the 4th country with the fastest urbanization in the last 25 years [22, 23]. The capital, Ouagadougou, has a population of 2,741,128. The majority of the population live in urban settings. About 45% of the population are under 15 years of age [24]. The city is divided into 12 districts and 52 sectors. Ouagadougou is the country's largest city and the cultural and economic center. The city is part of the Soudano-Sahelian area, with a rainfall of about 800 mm per year. The rainy season is from May to October, with a mean temperature of 28 °C (82 °F). The cold season runs from December to January, with a minimum average temperature of 16 °C (61 °F). During the hot season, which runs from March to May, the temperature can reach as high as 43 °C (109 °F).

The Health and Demographic Surveillance System is in place in Ouagadougou. Ouaga HDSS monitors a population of 81,717 residents; according to this surveillance system, the city population is very stable with a rate of migration of 4.1% and more than 80% of the inhabitants with ownership of their houses [20]. A map of the city and distribution of the population is shown in Figure 2.

Gabon, located on the west coast of Central Africa, has an area of nearly 270,000 square kilometres (100,000 sq. mi) with a population estimated at 1.5 million. Its capital and largest city is Libreville. In 2014, it is reported that 87% of the Gabonese population lived in urban areas [23]. The sixth largest city, Lambaréné, the capital of Moyen-Ogooué, is located 75 kilometers south of the equator, with a population of 25,257 in 2009. The majority of

For peer review only

Lambaréné residents live in semi-rural areas. About 42% of the Gabonese population is under 15 years of age [24]. Similarly, Lambaréné's population is relatively young with about 50% under 20 years of age.

The health services of Gabon are mostly public, but there are some private institutions as well. With one of the best medical infrastructure in the region, almost 90% of the population have access to health care services. Albert Schweitzer Hospital (ASH) is a private institution which served as a study site for the passive fever surveillance study [25, 26].

Kenya, located in East Africa, lies on the equator, covering 581,309 km^2 (224,445 sq. mi), with a population of approximately 45 million people in 2014 [2]. Kenya generally has a warm and humid tropical climate, but is diverse ranging from the cooler climate around the capital city, Nairobi, to a hot and dry climate inland, as well as a desert-like climate in the north-eastern regions along the border with Somalia and Ethiopia [27]. The capital, Nairobi, is a regional commercial hub. The main industries include agriculture, exporting tea and coffee, as well as the service industry.

Kenya is divided into 47 semi-autonomous counties. Mombasa is the country's second largest city after Nairobi, and is located on the east coast of the country [2]. Administratively, Mombasa is the capital of Mombasa County, what was formerly called Coast Province. This overall Coast region covers over 80,000 km^2 in the south-eastern part of Kenya, constituting about 15% of the country's land area, with a population of 3,325,307 residents.

The main driver of economy of Mombasa is tourism and trading industry. Mombasa itself has a population of about 1.3 million with almost 50% of the population under 15 years of age [24]. Increasingly, the population of the province lives in urban areas; at present about 45% live in Mombasa and other urban centers. The long rains begins around April and the

For peer review only

short rains begins in October [27]. Mean annual temperature ranges from 24°C to 27°C, but maximum temperature averages over 30°C during the hottest months, January to April.

Figure 3 shows the area of Mvita subcounty of Mombasa, which was the catchment area for the study in Kenya, with a catchment population of 74,735 residents. The map indicates the three facilities involved in the study.

Sample size

Given the paucity of available age-specific dengue incidence data in the study countries or nearby countries, it was difficult to obtain population-based incidence to make assumptions when calculating sample sizes. The required catchment population for the passive facility-based fever surveillance was roughly estimated based on the limited data available in the literature. Annual incidence estimates were calculated based on available prevalence estimates with the assumption that the outcome of interest has zero prevalence at age zero, and that force of infection is constant. It was assumed that prevalence estimates found for one particular age group would be adjusted as the annual incidence and used across all ages.

Wichmann et al. calculated an expansion factor for children by comparing data from three cohort studies to national surveillance data in Southeast Asia [28]. For children in Thailand, the age-specific expansion factors calculated were 11.85 for <5 years, 8.76 for 5-9 years, and 7.81 for 10-14 years [28]. The estimates used in our calculation were not from population-based studies and the degree of under-reporting of dengue in Africa is unknown. While it would have been ideal to adjust the incidence further for likely underestimation, the annual incidence used in sample size calculations could not be adjusted for possible under-reporting due to the lack of data. The sample sizes were calculated with 95% confidence levels and a margin of error at a fixed significance level within 25% of the true proportion of

For peer review only

incidence. This gives relative precision of 75%, considering the gap in evidence for dengue incidence in the study areas. The final sample sizes were calculated by assuming 20% non-response rate or loss to follow-up. The required catchment population size for the fever surveillance study in Burkina Faso was estimated to be 100,000, Gabon to be 77,000, and Kenya to be 70,000. In these catchment populations, the number of enrolled subjects depends on the number of eligible patients who seek care at the study facilities. How many eligible febrile episodes would actually present at our study facilities was difficult to predict; but after assessment of the volume of febrile patients at the facilities, a realistic upper limit for enrollment for a study period of approximately 1.5 years was set at 3,000 subjects to offer enrollment to all consenting eligible patients.

For the serological survey, the sample size was calculated similarly using the prevalence proportion based on published literature. Seroprevalence of 0.304 for Burkina Faso [10], 0.123 for Gabon [16], and 0.144 for Kenya [29] were used. With the same confidence levels and allowed margin of error, and assuming 5-15% non-response rate (variable by site), the sample size was calculated to be 3,000 participants at each site. Again, with the scarcity of data from the selected countries, there were no other prevalence estimates reported or estimates from different age groups. As prevalence is expected to increase with age, and higher prevalence would give a smaller sample size, our calculations are likely to be conservative.

Study components

Fever surveillance – design and methods

To determine burden due to symptomatic dengue in each of the three sites in Burkina Faso, Gabon, and Kenya, passive facility-based fever surveillance was implemented in a

For peer review only

well-defined catchment area population. In Burkina Faso, the surveillance study was initiated in December 2014 in five selected primary health care centres, locally called “Centre de Santé et de Promotion Sociale” (CSPS), in the municipality of Ouagadougou, with a catchment population of 105,000 residents. This project was implemented in collaboration with Centre Muraz in Bobo-Dioulasso, EQUITE sante program (a collaborative program between University of Montreal and Action-Gouvernance-Integration-Reinforcement, AGIR, based in Ouagadougou, funded by Canadian Institute of Health Research), and DVI. In Gabon, the surveillance study was initiated in the Albert Schweitzer Hospital serving a catchment population of 130,000 residents in the Moyen-Ogooué and surroundings within Lambaréné, in collaboration with CERMEL and Institute of Tropical Medicine in Tubingen, Germany. In Kenya, the surveillance study was implemented at Ganjoni dispensary, Tudor sub-county Hospital, and Coast Provincial General Hospital, serving a catchment population of 70,000 residents in Mombasa, in collaboration with Kenya Medical Research Institute (KEMRI) and Ministry of Health of Kenya.

As described in Fig. 2, both outpatients and inpatients at the designated study facilities, who meet inclusion criteria as mentioned earlier are tested for dengue, first with SD Dengue Duo® RDT. Dengue confirmation is done by detection of dengue virus in serum samples using PCR, as well as anti-dengue IgM and IgG antibodies in acute and convalescent serum by ELISA (SD Dengue IgM & IgG capture ELISA® tests, Standard Diagnostics, Yongin-Si, Korea) [30, 31]. Every consecutive patient meeting inclusion criteria is eligible for enrolment during the study period. Infants < 1 year old were not included due to operational limitations, such as difficulty of infantile bleeding.

In Ouagadougou, Burkina Faso, the fever surveillance initiated in December 2014

For peer review only

continued until February 2017 (approximately 2 years). In Lambaréné, Gabon, the fever surveillance initiated in April 2015 continued until January, 2017 (approximately 1.5 years). In Mombasa, Kenya, the fever surveillance initiated in March 2016 will continue until May 2017.

Among subjects enrolled in the fever surveillance, those who are positive by dengue rapid diagnostic test are offered further enrolment in the cost-of-illness survey, consisting of interviews on the day of acute illness visit, day 10-14 from the first visit, and day 28, if illness continues. The cost-of-illness survey questionnaire was designed to estimate the direct medical, direct non-medical, and indirect costs associated with dengue-positive patients identified at study facilities. This survey also estimates the cost of treating dengue at the facility level. Data are gathered by linking patients' medical records concerning outpatient visits, inpatient visits, and service consumption (e.g., diagnostic tests, medication, and other services provided to patients). The cost-of-illness portion of the study will be described separately.

Fever surveillance – laboratory testing

In all three sites, acute samples are tested using a commercial RDT for dengue NS1 and IgM/IgG (Dengue Duo®, Standard Diagnostics, Yongin-Si, Korea). Dengue Duo RDT is used on the day of acute illness visit at the site of patient presentation (day 1). The acute and convalescent samples are subsequently tested at a local laboratory using dengue IgM/IgG ELISA (SD Dengue IgM & IgG Capture ELISA®, Standard Diagnostics, Yongin-Si, Korea).

The serum is separated and stored in 4 aliquots of about 500 µL for various lab tests, as indicated in consent documents.

Samples with positive results by RDT or ELISA undergo further testing by RT-PCR.

For peer review only

Four DENV serotype-specific real-time RT-PCR assays are used for laboratory confirmation of dengue and serotyping [32]. The DENV 1-4 RT-PCR assays are carried out in 25μL reaction mixtures containing 5μL template RNA, TagMan® Fast Virus 1-step mastermix (Applied Biosystems®), 0.9 μM of each primer, and 0.2 μM probe [32]. Amplification and detection are performed in a StepOne Plus real-time PCR system and the baseline and threshold are determined using the auto-baseline and threshold feature in StepOne Software v2.2.2 (Applied Biosystems®). Thermocycling parameters are as follows: reverse transcription at 50 °C for 5 min, inactivation at 95 °C for 20 s, followed by 45 cycles of fluorescence detection at 95 °C for 3 s, and annealing at 60 °C for 30 s [32]. A specimen is considered positive if target amplification was recorded within 40 cycles.

Serological survey – design and methods

While the facility-based fever surveillance studies provide estimates of the burden of medically-attended dengue disease, evaluation of all DENV infections in a population – including subclinical and mildly symptomatic infections, which impact immune status – is needed to capture the overall impact of dengue. As part of the study package, population-based serological surveys were conducted in the same catchment population used for the fever surveillance. At each of the three sites in Africa, the serosurvey was conducted on a cohort of approximately 3,000 randomly selected residents of urban and semi-urban parts of Ouagadougou, Lambaréné, and Mombasa. Without individual-level census information on all residents of Lambaréné and Mombasa, with help of community/village health workers, randomization was done based on neighbourhoods (or defined areas for which the health workers/volunteers are responsible) as cluster units. As the community/village health workers

For peer review only

are familiar with the villages and their residents, they are good entry points into the communities. With these health workers, the field team screened houses in the selected villages by knocking on doors of every 5~7 houses, depending on the household density per neighbourhood. Also, demographic information collected in previous research projects conducted in the same area was used as a guide, if available. In the case of the site in Ouagadougou, HDSS data were available and the EQUITE CIHR research program of University of Montreal had set up a geographic information system (GIS) database system of houses in the study area. Using these data, households of potential enrollees of the serosurvey were pre-selected randomly and household visits were made. In the three sites, about 45% of the serosurvey samples were targeted to be collected from children 1 - 14 years-of-age, and 55% were targeted to be collected from adults between 15 and 55 years of age to reflect the age distribution of the general population of the area. Household-based enrollment was offered to the head of the household until the specific cap for the age-group was reached in Lambaréné and Mombasa.

Randomly-selected subjects 1-55 years of age underwent phlebotomy (5ml for children and 7ml for adults) twice — during pre-transmission (before the rainy season) and post-transmission (after the rainy season) at 6 month intervals. The sera were evaluated using IgG indirect ELISA at baseline and after 6 months. The presence of dengue IgG antibodies at 6 month intervals will be used to estimate the level of occurrence of inapparent DENV infection and to calculate the rate of infection in the catchment population. Flow cytometry-based DENV neutralization assays will be applied to a subset of samples to assess for presence of dengue neutralizing antibodies and seroconversion over the 6 month interval. In addition to overall seroconversion, age-specific seroconversion estimates in the catchment population as well as the proportion of inapparent infections are determined.

For peer review only

Serological survey – laboratory testing

From the samples collected in the serosurvey, about 200 µL of serum are used and tested at a local laboratory using dengue IgG ELISA (Panbio Dengue IgG Indirect ELISA®, Alere North America, LLC, Florida, United States). Given potential serological cross-reactivity among flaviviruses [33], flow cytometry-based neutralization assays will be performed against selected flaviviruses to include yellow fever virus, West Nile virus, Zika virus, and Japanese Encephalitis virus at the International Vaccine Institute, Seoul, Korea [34, 35].

About 1,000 µL of serum is kept aside for this procedure. The flow cytometry-based neutralization assays are performed in duplicate in 96-well cell culture plates with flat-bottom wells, each containing DC-SIGN-expressing U937 cells [34]. The amount of virus used in the assay infects between 7 and 15% of the cells. Human immune sera are serially diluted and the virus is pre-incubated with the sera for 1 h at 37°C [34]. The cells are washed, and the virus and serum mixture is added to the cells for 1 h at 37°C, and the cells are further incubated for 24 to 48 h at 37°C in 5% CO_2. The cells are fixed, permeabilized, and stained with fluoresce-conjugated monoclonal antibody 4G2, which recognizes the flavivirus E protein [36]. FACScan flow cytometer (Becton Dickinson, San Diego, CA) is used to analyze the cells [34]. The serum dilution that neutralized 50% of the viruses is calculated by nonlinear, dose-response regression analysis with Prism 4.0 software (GraphPad Software, Inc., San Diego, CA).

In addition, a Luminex-based multiplex immunoassay will be performed on a randomly selected sub-sample to assess for IgG to different flaviviruses [37]. Detection of IgG against ZIKV and each the four DENV serotypes was performed on patient serum

For peer review only

samples using an in-house microsphere-based multiplex immuno-assay (arbo-MIA) [38, 39]. The arbo-MIA is based on a mixture of microspheres covalently coupled with either DENV-1, -2, -3, -4 or ZIKV recombinant antigens (E protein domain III) produced in Drosophila S2 expression system. Briefly, microsphere mixtures were sequentially incubated in the dark under constant shaking with a 1:400 dilution of patient serum samples, with 2 µg/mL anti-human IgG biotin-conjugated antibody (Jackson Immunoresearch, West Grove, PA) and with 2 µg/mL streptavidin-R-phycoerythrin conjugate (Life technologies). After the final incubation, the median fluorescence intensity (MFI) of each microsphere set was quantified using a BioPlex 200 instrument (Bio-Rad Laboratories, Hercules, CA). Samples were considered seropositive if the ratio of MFI values obtained for the viral antigen to the control antigen was superior to the defined cut-off. The cut-off of the MIA was determined for each viral antigen by ROC curve analysis using well characterized sera.

In Lambaréné, the enrolment bleed, took place in November -December 2015 with 2nd blood collection in May 2016. In Ouagadougou, the enrolment bleed took place in May-June 2015 with follow-up blood collections in December 2015, June 2016, and January 2017. In Mombasa, enrolment bleed took place in May 2016 with the 2nd blood collection in November 2016 – February 2017.

Healthcare Utilization Survey

As the passive fever surveillance is conducted at the study facilities, potential dengue patients could be missed if they seek care elsewhere. To identify the proportion of fever and dengue cases potentially missed by the passive surveillance system due to patients living in the study area but seeking care outside of study facilities, a population-based healthcare utilization survey was conducted in 400 randomly selected households from the study

For peer review only

catchment area to characterize their healthcare utilization patterns of the households when they have (self-reported) febrile episodes among the family members. In addition to assessing health-seeking behaviours of the residents, preferences in terms of health-seeking behavior and respective reasons for their preferences were investigated. The questionnaire was administered to 400 heads of households. Among 3,000 residents who participated in the serosurvey, there were about 600 households. From these households, 400 heads of households were randomly selected and offered enrolment in the health utilization survey. Heads of households or a senior representative within the household were asked questions on health seeking patterns of their family members. The surveys provide data to determine the proportion of these cases missed by our passive fever surveillance system. Also, the surveys document health seeking patterns, who would seek care at which facilities, who would make health-seeking related decisions, and the reasons for their preferences.

Study questionnaires

For the fever surveillance study, questionnaires are administered at the acute illness visit and the convalescent visit. The convalescent visit may take place at the health care facility (10-14 days later) or at the patient's home (15-21 days after the acute visit), according to patient preference and availability. The questionnaires are completed by medical staff of the study facilities, including demographic and clinical information (e.g., signs, symptoms, past medical history, treatments prescribed, and diagnoses). The same staff also complete the follow-up questionnaire at the convalescent visit within 21 days from the acute visit. Study nurses complete surveillance enrolment log. Lab technicians complete the lab section (mostly dengue-related diagnostics) and the forms are compiled by the study coordinator on site.

For the serosurvey component, questionnaires are administered at the household by trained field team staff at each serosurvey visit. Study nurses complete the questionnaire after a brief

For peer review only

physical and medical examination. There are (a) follow-up visit(s) in about 6 months and the same staff make the household visits to complete the follow-up questionnaire. Enrolment log is maintained by our study coordinator on site.

Variables of the surveillance questionnaires

The variables collected are listed in table 1.

Table 1. List of variables collected in the passive fever surveillance data collection form

**Topic**	**Description**	**Items**
Basic information	Demographic and basic information about the patient and the treatment received	Type of treatment where patient is enrolled (IPD vs. OPD)<br>Date of fever onset, duration of fever<br>Current temperature<br>Tourniquet test results<br>Patient's address (district and village-level)<br>Date of visit, date of birth, age, and sex<br>Weight and height
General health condition	Current condition of the patient (self-report) and underlying diseases of the patient	How well the patient could handle daily activities<br>Pre-existing conditions
Signs and symptoms during this illness	A set of sign and symptoms that may be related to fever and dengue (DF and DHF)	Rash, fatigue, headache, retro-orbital pain, neck/ear pain, sore throat, breathing difficulty, cough, expectoration, gastrointestinal signs (Nausea/vomiting, diarrhea, abdominal pain, etc.), hemorrhagic signs (nose/gum bleeding, ecchymosis, petechiae, etc.), signs of shock (cyanosis, capillary refill), arthralgia, myalgia, loss of appetite, jaundice, etc.
Medical History:	Previous dengue-related or other flavivirus infection as well as vaccination history (self-report)	Previous dengue infection and related hospitalization<br>Previous infection to other commonly circulating arboviral infection in the area<br>Yellow fever vaccination history
Laboratory findings	Records from the routine laboratory tests widely used in	Platelet count, hematocrit, haemoglobin, leukocytes, neutrophils,

For peer review only

	clinical fever/dengue patient management, as part of the hospital care procedure	protein level, AST, ALT, urine test results, etc.
Clinical Diagnosis	Clinician's diagnosis with or without referring to the RDT	Diagnosis given by the physician based on clinical presentation after physical examination of the patient.
Dengue testing results	Results from the dengue tests, mainly RDTs for dengue as well as other commonly circulating arbovirus in the area	Dates of blood draw<br>Test results of the RDT<br>IgM/IgG capture ELISA results<br>PCR results (if available)
Treatment	Medicine(s) prescribed and the starting and end dates	Antibiotics, paracetamol, ibuprofen, aspirin, and others that may be site-specifically prescribed
Outcome	Outcome of this particular visit	Hospitalized, returned home, or referral
Hospitalization	Information collected only among hospitalized patients in the surveillance to record other severe signs and progression of illness	Admission and discharge diagnoses<br>Presence of haemorrhagic signs or shock syndrome
Hospital Charges	Expenses and hospital charges incurred by patient on the visit 1	Amount of the out of pocket payment by the patient or the family/or guardian<br>Breakdown of the hospital charges (laboratory, medication, admission-related charges)
Final outcome	Outcome of the patient's illness at the 2nd visit	Final diagnosis given for the patient outcome of illness<br>Completion of study participation (early termination and the reason, etc.)

Planned statistical analysis

From the fever surveillance data, incidence of symptomatic dengue among patients that seek health care at the study facilities will be calculated. Age-specific incidence rates in all the children and adults will be determined by referring to the size and distribution of the general population of the study area at the time of surveillance as the denominator in calculation of the incidence of symptomatic dengue cases. Each person residing in the study area is assumed to contribute 12 months of person time to the denominator. Although the

For peer review only

study areas all report a low migration rate, the in-migration is assumed to balance the out-migration of the population during the study period. Age-specific incidence of symptomatic dengue will be calculated by using age-specific denominators and the number of symptomatic dengue cases in eligible individuals as the numerator.

Using the data collected in the Healthcare Utilization Survey, the proportion of febrile cases missed by the passive surveillance system will be determined. Then using the proportion, the numerator will be further adjusted in recognition of those missed fever cases from the study area, which could have been dengue. Also, comparison will be made between those that agreed to participate and those that declined participation so that our sample of febrile cases is representative of febrile patients of the general population of the catchment area.

SPSS software will be used for analysis of the fever surveillance data. Multivariable logistic regression will be used to compare confirmed dengue patients versus non-dengue febrile patients in terms of symptomatic presentation, based on signs and symptoms collected from all patients with laboratory-confirmed dengue by serology and RT-PCR, adjusting for possible confounders, such as age, days since onset of fever, primary vs. secondary infection, inpatient vs. outpatient, etc. Differences in symptomatic complex of DF (and DHF, if data allows) by age and serotype will be also determined using multivariable logistic regression.

As outpatient disease accounts for the greater part of dengue disease burden, clinical profile of individuals with DENV infection will be characterized by the type of treatment (hospitalized and outpatients), as well as by severity of the disease (severe vs. non-severe by the 2009 WHO criteria) [40]. Classification is determined after the course of illness is completed (typically during the convalescent visit). Symptomatic dengue is classified as outpatient or hospitalized. Progression of dengue is recorded in the CRF as DF, DHF I, DHF II, DHF III or DHF IV and clinical patterns will be compared by the severity grade [40, 41].

For peer review only

These will be compared to results obtained from other DVI studies in Latin America (Colombia) and Asia (Thailand, Vietnam, and Cambodia). Overall, comparisons will be made across Burkina Faso, Gabon and Kenya.

With the age-stratified sera that reflect age distribution of the general population of the country, the serological survey sampling strategy ensures sufficient subjects to obtain precise age-specific estimates of sero-positivity and sero-conversion of the catchment area population. The sero-conversion rate and change in the immune status will be determined by age group during the study period. The age-stratified serosurvey data will also allow calculation of the force of infection of dengue in the study population. Comparisons will be made among Burkina Faso, Gabon and Kenya.

Ethical considerations

To minimize inconvenience of the study to patients, clinicians and nurses were sensitized and trained regarding the study requirements and procedures in order for data collection to be integrated into routine patient care. The clinicians and nurses selected for the study receive coordinated support from study field staff throughout the study process. Written informed consent, and assent for participants 7 (13 for Kenya) -17 years of age, was obtained from patients by study staff. Study staff go through consent and assent documents for short summary of the disease, detailed description of study procedure, and information on reimbursement. Patient data are documented in the study designated office and only the study staff have access to the data that had been de-identified without any personal identifiable information. Data are exclusively handled in the study office and stored safely in a protected database in the study office as well as the DVI main server.

The protocol for each study obtained ethical approvals from the Institutional Review Boards (IRBs) of the International Vaccine Institute, the London School of Hygiene and

For peer review only

Tropical Medicine, and the Ethics Committee of host country institutions, including KEMRI Scientific and Ethical Review Unit in Kenya, Gabon National Ethics Committee and Institutional Ethics Committee, Scientific Review Board of CERMEL in Gabon, and the IRB of Centre Hospitalier de l'Université de Montréal (CRCHUM) at University of Montreal, and the National Health Ethical Committee of Burkina Faso.

Results and discussion

Despite the fact that dengue cases and *Aedes* mosquitoes have been reported in Africa as early as 1823 [5], very few dengue studies have been conducted in Africa. Also, little evidence is from population-based studies [6]. Compared to the volume of evidence from SE Asia and the Americas, there is critical data scarcity on dengue in Africa. Suspicion of substantial dengue burden in Africa is based on limited reports of outbreaks and a handful of sero-prevalence studies testing different viruses among samples that likely do not represent the general population. In the three countries selected for our field studies, somewhat more data is available but is also very limited. In Burkina Faso, recent reported incidence was 8.7% based on dengue RDT from an observational study conducted in 2013 [11]. In Gabon, one study suggested minimal DENV circulation in rural areas [16], while a recent study reported 12.3% seroprevalence among toddlers 30 months of age in semi-rural parts of Lambaréné [15]. In Kenya, about 13% of the individuals in Mombasa have been reported evidence of past or current DENV infection [21]. Despite the limited scope and generalizability of these studies, they suggest that there may be more dengue than previously appreciated due to underestimation and misdiagnosis [20, 21].

These studies all indicate presence of dengue and some level of underlying sero-prevalence in the countries of our field studies. However, often these studies are limited by their retrospective design or sample collection (blood donors or sample collected from

For peer review only

surveys of other diseases) in terms of assessing the true, population-based, burden of dengue. We proposed to address this gap by population-based dengue surveillance and sero-prevalence studies in West, (West-) Central, and East Africa.

The present studies at three sites in Africa will provide important information on undocumented DENV circulation in Africa. Such data will help to strengthen the evidence base for dengue burden in Africa. Better defined disease burden data based on our studies could be used to assess the relative need for dengue prevention and control measures, such as whether a dengue vaccine would be a cost-effective public health intervention for countries in Africa. Clinical findings from our studies could also be used as a guide for dengue case detection and case management.

The studies have some important limitations. One potential source of bias in estimating the incidence of symptomatic dengue is under-ascertainment due to the community residents with relevant symptoms seeking care from other healthcare providers and facilities than the facility under surveillance. As the study design remains passive surveillance, cases are ascertained only at study facilities. By estimating the proportion of febrile patients seeking care elsewhere, the degree of fever patients missed by the study facility will be determined. Also, depending on the transmission volume of dengue or other co-circulating diseases with onset of fever there may be patients that are diagnosed with other diseases, and ruled out of dengue. These factors may increase the likelihood of under-reporting or over-reporting.

In addition, the sero-survey and healthcare utilization survey are conducted on a randomized sub-sample of the catchment area population and there may be limited generalizability of the data collected from these surveys. With unknown differences among those that agree to participate and those that do not agree, the data may not be representative of the general population of the study countries.

For peer review only

Conclusions

Data collection continues and study closure is planned in all three sites in April – May, 2017. Although not described in this paper, preliminary results indicate a substantial level of dengue incidence and prevalence in each site. The data collected from our studies will provide a more accurate assessment of the unknown dengue disease burden in Burkina Faso, Gabon, and Kenya. These data can fill a gap in undocumented burden of dengue in the region and, collectively, may be used to infer dengue burden in other areas of Western, Central, and Eastern Africa. Countries in Africa may not consider introduction of a dengue vaccine as the foremost priority in the near future due to many other competing public health problems and limited resources as a major challenge. For cost-effective implementation of public health interventions, accurate data on dengue burden from epidemiological studies would be needed for policy makers to make evidence-based decisions on control and prevention of dengue. Our studies will provide some much needed information based on population-based research to assess dengue burden in Africa.

For peer review only

List of abbreviations

GDAC - Global Dengue and *Aedes*-transmitted Diseases Consortium
IVI - International Vaccine Institute
DENV- dengue viruses
DVI - Dengue Vaccine Initiative
Ouaga-HDSS - health and demographic surveillance system
DHF - dengue hemorrhagic fever
CERMEL - Centre de Recherches Médicales de Lambaréné
ASH – Albert Schweitzer Hospital
CSPS - Centre de Santé et de Promotion Sociale
KEMRI - Kenya Medical Research Institute
SD – Standard Diagnostics
GIS - geographic information system
MFI - median fluorescence intensity
CRCHUM - Centre Hospitalier de l'Université de Montréal

For peer review only

Declarations

- Ethics approval and consent to participate

The protocol for each study obtained ethical approvals from the Institutional Review Boards (IRBs) of the International Vaccine Institute, the London School of Hygiene and Tropical Medicine, and the Ethics Committee of host country institutions, including KEMRI Scientific and Ethical Review Unit in Kenya, Gabon National Ethics Committee and Institutional Ethics Committee, Scientific Review Board of CERMEL in Gabon, and the IRB of Centre Hospitalier de l'Université de Montréal (CRCHUM) at University of Montreal, and the National Health Ethical Committee of Burkina Faso.

- Consent for publication

Not applicable

- Availability of data and material

Data sharing is not applicable to this article as no datasets were analyzed during the current study.

This manuscript does not include data from the studies described here in. This is a protocol paper. The datasets that are being generated for analysis as described in the current study are not yet publicly available as the studies are currently ongoing at the time of submission. They will be available from the corresponding author on reasonable request.

- Competing interests

The authors declare that they have no competing interests.

- Funding

The current study was supported by funds from the Bill and Melinda Gates Foundation (OPP 1053432). The funding body had no role in the design of the study and collection, analysis, and interpretation of data and in writing the manuscript.

For peer review only

- Authors' contributions

JKL designed the study, is overseeing data collection, and was a major contributor in writing the manuscript. MC co-designed the study, oversaw some parts of data collection, and supported in writing of the manuscript. JSL was a contributor in designing of the study and oversight of parts of data collection. KSL was a contributor in oversight of data collection. SN supported in data collection. SKL supported in data collection. VR supported in designing of the study and was a major contributor in finalization of the manuscript. JF was a contributor in data collection. BL was a contributor in designing of the study and data collection. SHM was a contributor in designing of the study and site establishment. ME was a contributor in designing of the study. EA supported in data collection. NO supported in data collection. AB supported in data collection. EB supported in data generation. SMN was a contributor in designing of the study and site establishment. STA was a contributor in designing of the study and site establishment. SY was a contributor in designing of the study and site establishment. NA was a major contributor in providing oversight of the data collection and finalization of the manuscript. IKY was a major contributor in designing of the study and finalization of the manuscript.
All authors read and approved the final manuscript.

- Acknowledgements

We thank the doctors and laboratory staff of CSPS of Ouagadougou, ASH, Ganjoni dispensary, Tudor sub-county Hospital, and Coast Provincial General Hospital, as well as at Centre Muraz, CERMEL, and KEMRI. We thank collaborators at AGIR, IRD France, and University of Montreal, and University of Tubingen. Lastly, we would like to thank the DVI team and statisticians and administrative staff at the International Vaccine Institute for their helpful comments during the preparation of this manuscript and support during the studies.

For peer review only

References

1. Bhatt S, Gething PW, Brady OJ, Messina JP, Farlow AW, Moyes CL, Drake JM, Brownstein JS, Hoen AG, Sankoh O *et al*: **The global distribution and burden of dengue**. *Nature* 2013, **496**(7446):504-507.
2. Murray NE, Quam MB, Wilder-Smith A: **Epidemiology of dengue: past, present and future prospects**. *Clin Epidemiol* 2013, **5**:299-309.
3. Chang M, Christophel E, Gopinath D, Abdur R, on behalf of Malaria OVaPD, World Health Organization Regional Office for the Western Pacific: **Challenges and future perspective for dengue vector control in the Western Pacific Region, Regional Analysis**. *Western Pacific Surveillance and Response Journal* 2011, **2**(2):9-16.
4. Eisen L, Beaty B, Morrison A, Scott T: **ProactiveVector control strategies and improved monitoring and evaluation practices for dengue prevention.** *J Med Entomol* 2009, **46**(6):1245-1255.
5. Messina JP BO, Scott TW, Zou C, Pigott DM, Duda KA, Bhatt S, Katzelnick L, Howes RE, Battle KE, Simmons CP, Hay SI.: **Global spread of dengue virus types: mapping the 70 year history.** *Trends Microbiol* 2014, **22**(3):138-146.
6. Amarasinghe A KJ, Letson GW, Margolis HS.: **Dengue virus infection in Africa.** *Emerg Infect Dis* 2011, **17**(8):1349-1354. .
7. Beatty ME SA, Fitzsimons DW, Hanna JN, Lam SK, Vong S, Guzman MG, Mendez-Galvan JF, Halstead SB, Letson GW, Kuritsky J, Mahoney R, Margolis HS; Asia-Pacific and Americas Dengue Prevention Boards Surveillance Working Group.: **Best practices in dengue surveillance: a report from the Asia-Pacific and Americas Dengue Prevention Boards.** *PLoS Negl Trop Dis* 2010, **4**(11):e890.
8. Were F: **The dengue situation in Africa**. *Paediatrics and Internatinoal Child Health* 2012, **32**(S1):18-21.
9. Brady OJ, Gething PW, Bhatt S, Messina JP, Brownstein JS, Hoen AG, Moyes CL, Farlow AW, Scott TW, Hay SI: **Refining the global spatial limits of dengue virus transmission by evidence-based consensus**. *PLoS neglected tropical diseases* 2012, **6**(8):e1760.
10. Collenberg E, Ouedraogo T, Ganame J, Fickenscher H, Kynast-Wolf G, Becher H, Kouyate B, Krausslich HG, Sangare L, Tebit DM: **Seroprevalence of six different viruses among pregnant women and blood donors in rural and urban Burkina Faso: A comparative analysis**. *J Med Virol* 2006, **78**(5):683-692.
11. Ridde V, Agier I, Bonnet E, Carabali M, Dabiré K, Fournet F, Ly A, Meda I, Parra B: **Presence of three dengue serotypes in Ouagadougou (Burkina Faso): research and public health implications.** *Infect Dis Poverty* 2016, **5**(23).
12. Caron M, Grard G, Paupy C, Mombo IM, Bikie Bi Nso B, Kassa Kassa FR, Nkoghe D, Leroy EM: **First evidence of simultaneous circulation of three different dengue virus serotypes in Africa**. *PloS one* 2013, **8**(10):e78030.
13. Leroy EM, Nkoghe D, Ollomo B, Nze-Nkogue C, Becquart P, Grard G, Pourrut X, Charrel R, Moureau G, Ndjoyi-Mbiguino A *et al*: **Concurrent chikungunya and dengue virus infections during simultaneous outbreaks, Gabon, 2007**. *Emerging infectious diseases* 2009, **15**(4):591-593.
14. Caron M, Paupy C, Grard G, Becquart P, Mombo I, Nso BB, Kassa Kassa F, Nkoghe D, Leroy EM: **Recent introduction and rapid dissemination of Chikungunya virus and Dengue virus serotype 2 associated with human and mosquito coinfections in Gabon, central Africa**. *Clinical infectious diseases : an official publication of the Infectious Diseases Society of America* 2012, **55**(6):e45-53.

15. Gabor J, Schwarz N, Esen M, Kremsner P, Grobusch M: **Dengue and chikungunya seroprevalence in Gabonese infants prior to major outbreaks in 2007 and 2010: A sero-epidemiological study.** *Travel Med Infect Dis* 2016.
16. Pourrut X, Nkoghe D, Gonzalez JP, Leroy E: **No evidence of dengue virus circulation in rural Gabon**. *Emerging infectious diseases* 2011, **17**(8):1568-1569.
17. Andries A, Duong V, Ngan C, Ong S, Huy R, Sroin K, Te V, Bunthin Y, Try P, P B: **Field evaluation and impact on clinical management of a rapid diagnostic kit that detects dengue NS1, IgM and IgG**. *PLoS Negl Trop Dis* 2012, **6**(12):e1993.
18. Lell B, Agnandji S, von Glasenapp I, Haertle S, Oyakhiromen S, Issifou S, Vekemans J, Leach A, Lievens M, Dubois MC *et al*: **A randomized trial assessing the safety and immunogenicity of AS01 and AS02 adjuvanted RTS,S malaria vaccine candidates in children in Gabon.** *PLoS One* 2009, **4**(10):e7611. .
19. Agnandji ST, Lell B, Soulanoudjingar SS, Fernandes JF, Abossolo BP, Conzelmann C, Methogo BG, Doucka Y, Flamen A, Mordmüller B *et al*: **First results of phase 3 trial of RTS,S/AS01 malaria vaccine in African children.** *N Engl J Med* 2011, **365**(20):1863-1875. .
20. Ochieng C AP, Vittor AY, Nyoka R, Gikunju S, Wachira C, Waiboci L, Umuro M, Kim AA, Nderitu L, Juma B, Montgomery JM, Breiman RF, Fields B.: **Seroprevalence of Infections with Dengue, Rift Valley Fever and Chikungunya Viruses in Kenya, 2007**. *PLoS One* 2015, **10**(7).
21. Ellis EM, Neatherlin JC, Delorey M, Ochieng M, Mohamed AH, Mogeni DO, Hunsperger E, Patta S, Gikunju S, Waiboic L *et al*: **A household serosurvey to estimate the magnitude of a dengue outbreak in Mombasa, Kenya, 2013**. *PLoS Negl Trop Dis* 2015, **9**(4):e0003733.
22. United Nations Development Programme: **Population, urban (% of population)**. In. Edited by Reports HD, vol. 2016: UNDP; 2013.
23. United Nations: **World Urbanization Prospects: The 2014 Revision, Highlights**. In. Edited by Department of Economic and Social Affairs PD. United Nations: United Nations; 2014.
24. Central Intelligence Agency: **Age structure**. In: *The world factbook* vol. 2016: Central Intelligence Agency.
25. Ramharter M, Adegnika AA, Agnandji ST, Matsiegui PB, Grobusch MP, Winkler S, Graninger W, Krishna S, Yazdanbakhsh M, Mordmüller B *et al*: **History and perspectives of medical research at the Albert Schweitzer Hospital in Lambaréné, Gabon**. *The Middle European Journal of Medicine, WIENER KLINISCHE WOCHENSCHRIFT* 2007, **119**(Suppl 3):8–12.
26. Schwarz NG, Gysels M, Pell C, Gabor J, Schlie M, Issifou S, Lell B, Kremsner PG, Grobusch MP, Pool R: **Reasons for non-adherence to vaccination at mother and child care clinics (MCCs) in Lambaréné, Gabon**. *Vaccine* 2009, **27**(2009):5371-5375.
27. **Kenya** [https://en.wikipedia.org/w/index.php?title=Kenya&oldid=737329303]
28. Wichmann O YI, Vong S, Limkittikul K, Gibbons R, Mammen MP, et al.: **Dengue in Thailand and Cambodia: An assessment of the degree of underestimated disease burden based on reported cases.** . *PLoS NTD* 2010, **Accepted in Feb. 2010.**
29. Mease L, Coldren R, Musila L, Prosser T, Ogolla F, Ofula V, Schoepp R, Rossi C, Adungo N: **Seroprevalence and distribution of arboviral infections among rural Kenyan adults: a cross-sectional study.** *Virol J* 2011, **8**(371).
30. Beatty ME, Stone A, Fitzsimons D, Hanna JN, Lam SK, Vong S, Guzman MG, Mendez-Galvan J, Halstead S, Letson GW *et al*: **Best Practices in Dengue**

For peer review only

Surveillance: A Report from the Asia-Pacific and Americas Dengue Prevention Boards. *PLoS NTD* 2010, ***Submitted***.
31. World Health Organization: **Guidelines for the clinical evaluation of dengue vaccines in endemic areas**. In. Geneva: World Health Organization; 2008.
32. Alm E, Lindegren G, Falk K, Lagerqvist N: **One-step real-time RT-PCR assays for serotyping dengue virus in clinical samples.** *BMC Infect Dis* 2015, **15**(493).
33. Peeling R, Artsob H, Pelegrino J, Buchy P, Cardosa M, Devi S, Enria D, Farrar J, Gubler D, Guzman M *et al*: **Evaluation of diagnostic tests: dengue**. *Nature Reviews Microbiology* 2010:S30-S37
34. Kraus A, Messer W, Haymore L, de Silva A: **Comparison of plaque- and flow cytometry-based methods for measuring dengue virus neutralization**. *J Clin Microbiol* 2007, **45**(11):3777-3780.
35. de Alwis R, de Silva A: **Measuring antibody neutralization of dengue virus (DENV) using a flow cytometry-based technique**. *Methods Mol Biol* 2014, **1138**:27-39.
36. Lambeth C, White L, Johnston R, de Silva A: **Flow cytometry-based assay for titrating dengue virus.** *J Clin Microbiol* 2005, **43**(7):3267-3272.
37. Cabral-Castroa M, Saramago Peraltab R, Cavalcantia M, Puccioni-Sohlerd M, Carvalhoe V, da Costa Vasconcelose P, Peraltaa J: **A Luminex-based single DNA fragment amplification assay as a practical tool for detecting and serotyping dengue virus**. *Journal of virological methods* 2016, **236**:18-24.
38. Balasuriya U, Shi P, Wong S, Demarest V, Gardner I, Hullinger P, Ferraro G, Boone J, De Cino C, Glaser A *et al*: **Detection of antibodies to West Nile virus in equine sera using microsphere immunoassay.** *J Vet Diagn Invest* 2006, **18**(4):392-395.
39. Beck C, Desprès P, Paulous S, Vanhomwegen J, Lowenski S, Nowotny N, Durand B, Garnier A, Blaise-Boisseau S, Guitton E *et al*: **A High-Performance Multiplex Immunoassay for Serodiagnosis of Flavivirus-Associated Neurological Diseases in Horses.** *Biomed Res Int* 2015, **2015**(678084).
40. Hadinegoro SRS: **The revised WHO dengue case classification: does the system need to be modified?** *Paediatr Int Child Health* 2012, **32**(S1):33-38.
41. Guilarde AO, Turchi MD, Siqueira Jr JB, Feres VCR, Rocha B, Levi JE, Souza VAUF, Boas LSV, Pannuti CS, Martelli CMT: **Dengue and Dengue Hemorrhagic Fever among Adults: Clinical Outcomes Related to Viremia, Serotypes, and Antibody Response**. *The Journal of Infectious Diseases*, **197**(6): 817-824. .

For peer review only

Fig. 1 Description of the study components, including passive facility-based fever surveillance, healthcare utilization surveys, cost-of-illness surveys, and serological surveys

There are two arms in the study package, composed of four parts. In the health facility-based arm of the study package, there are the passive facility-based fever surveillance and cost-of-illness survey embedded within the surveillance. In the community arm of the study, there are serological survey and healthcare utilization survey.

For peer review only

Fig. 2 A map of the city and distribution of the population in Ouagadougou, Burkina Faso

Fig. 3 A map of the city and distribution of the population in Lambaréné, Gabon

Fig. 4 A map of the city and distribution of the population in Mombasa, Kenya

Figures 2 – 4 show the map of the study area at each site in Burkina Faso, Gabon, and Kenya.

For peer review only

Fig. 5 Patient flow in the fever surveillance

Eligible febrile patients identified and enrolled as study subjects will follow these steps to complete participation in the passive fever surveillance.

For peer review only

Fig. 6 Laboratory testing algorithm for dengue

Samples from subjects of the passive fever surveillance would follow these steps of the testing algorithm for confirmation of dengue.

For peer review only

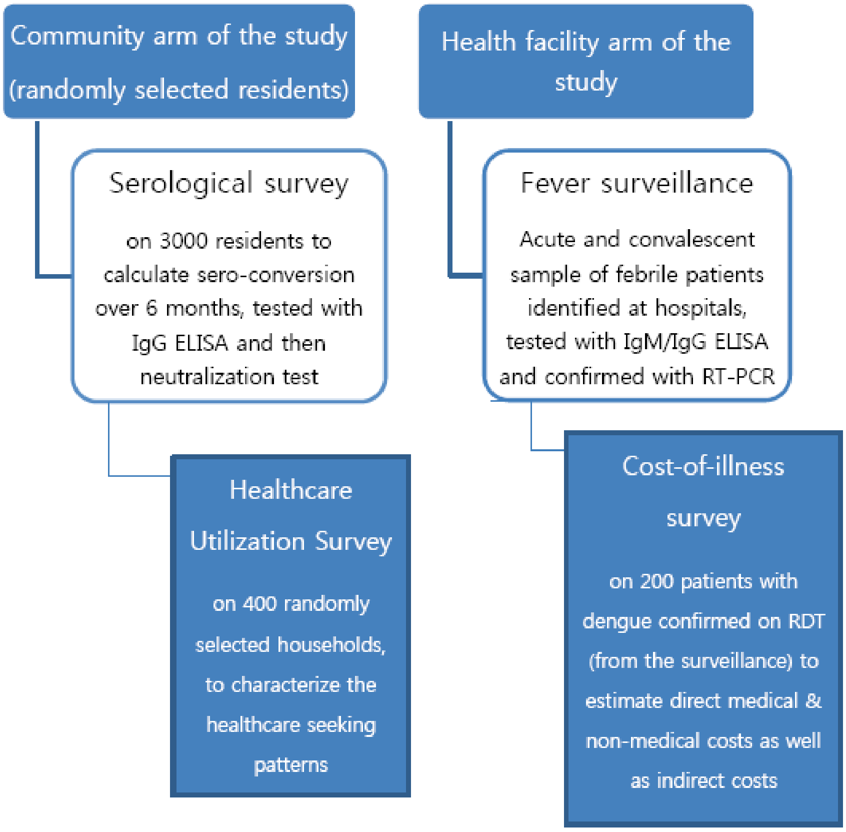

For peer review only

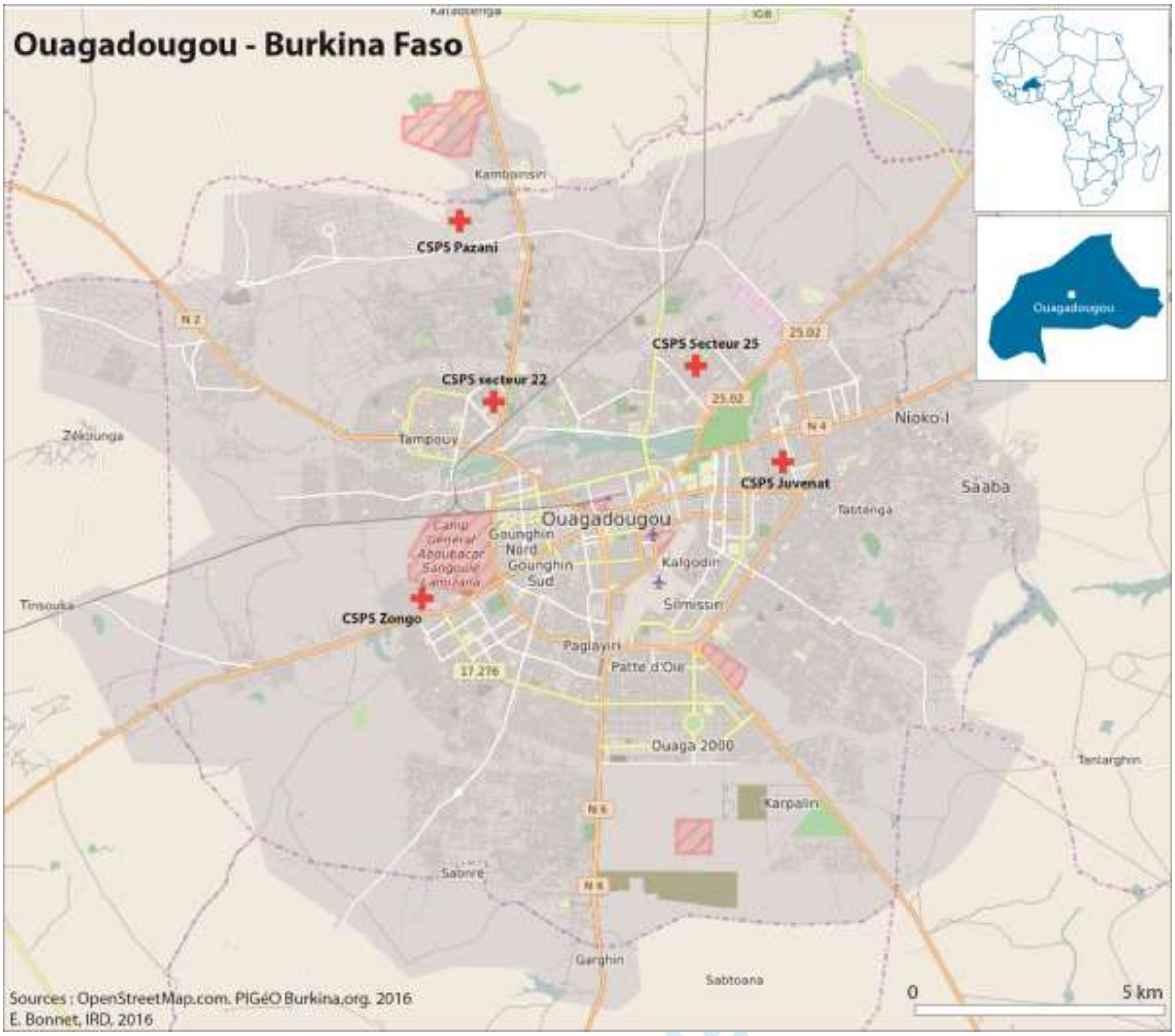

For peer review only

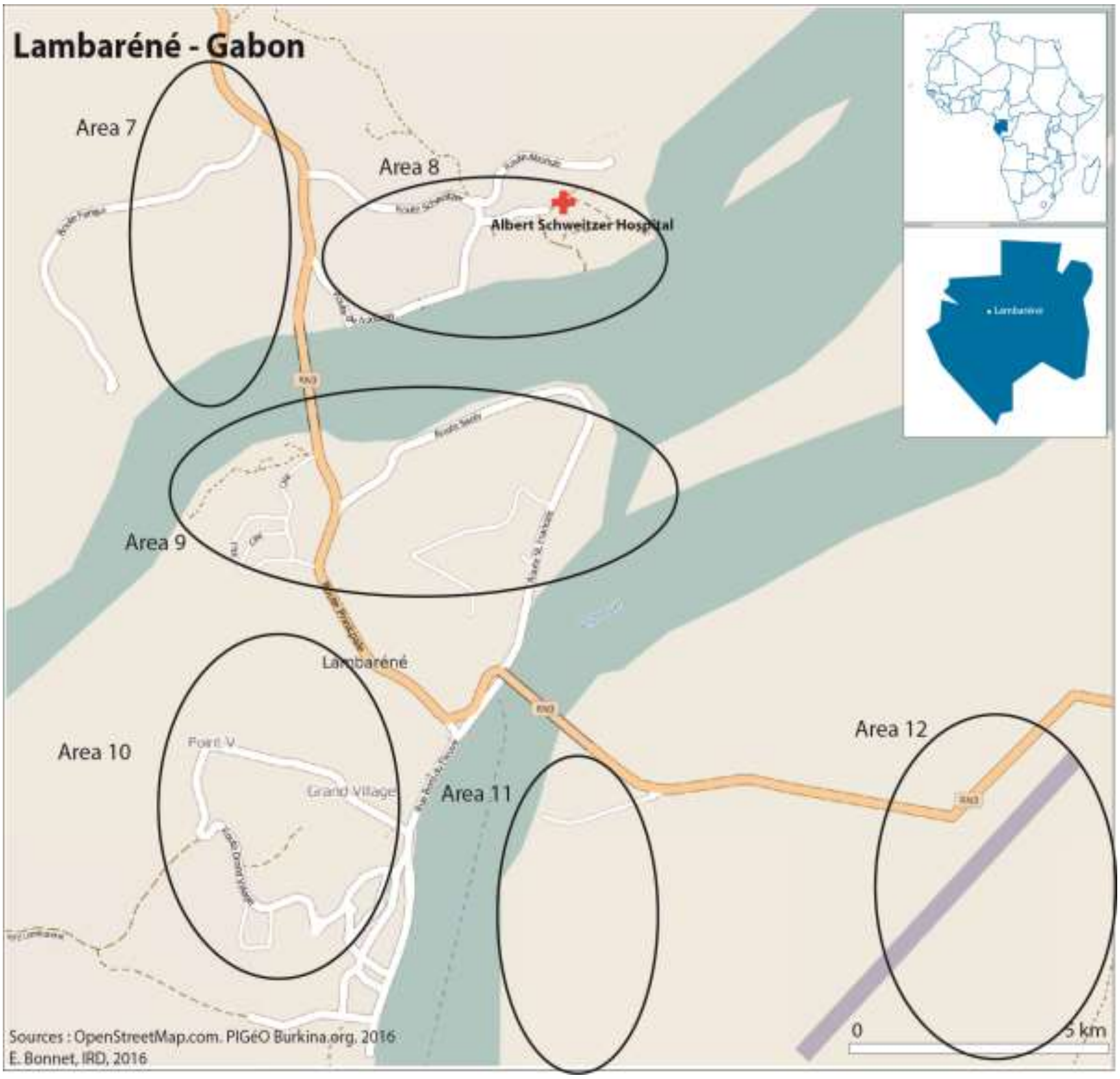
Lambaréné - Gabon
Area 7
Area 8
Albert Schweitzer Hospital
Area 9
Lambaréné
Area 10
Grand Village
Area 11
Area 12
0
5 km
Sources : OpenStreetMap.com. PIGéO Burkina.org. 2016
E. Bonnet, IRD, 2016

only

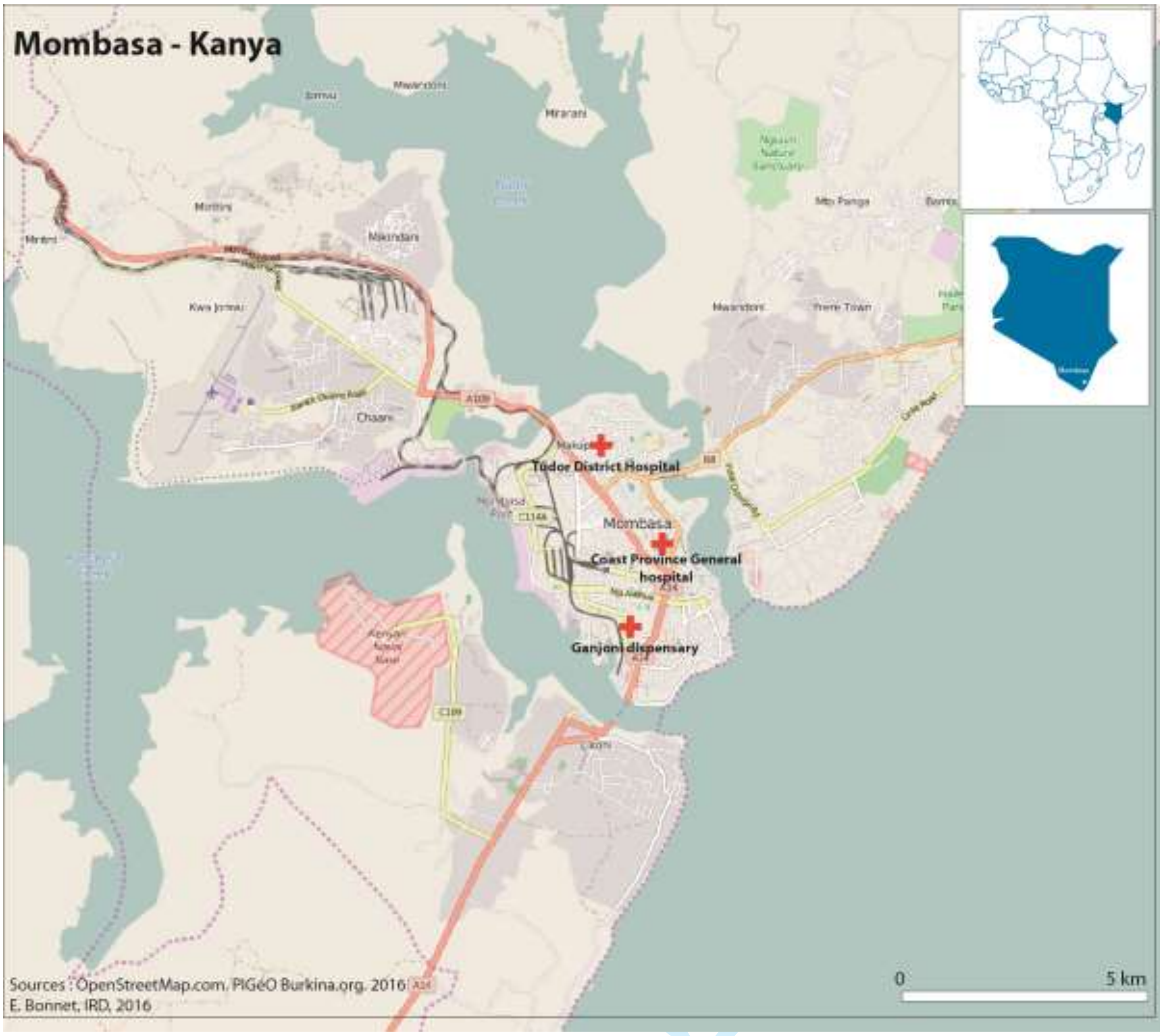
Mombasa - Kanya
Tudor District Hospital
Mombasa
Coast Province General hospital
Ganjoni dispensary
Sources : OpenStreetMap.com, PIGéO Burkina.org, 2016
E. Bonnet, IRD, 2016
0
5 km

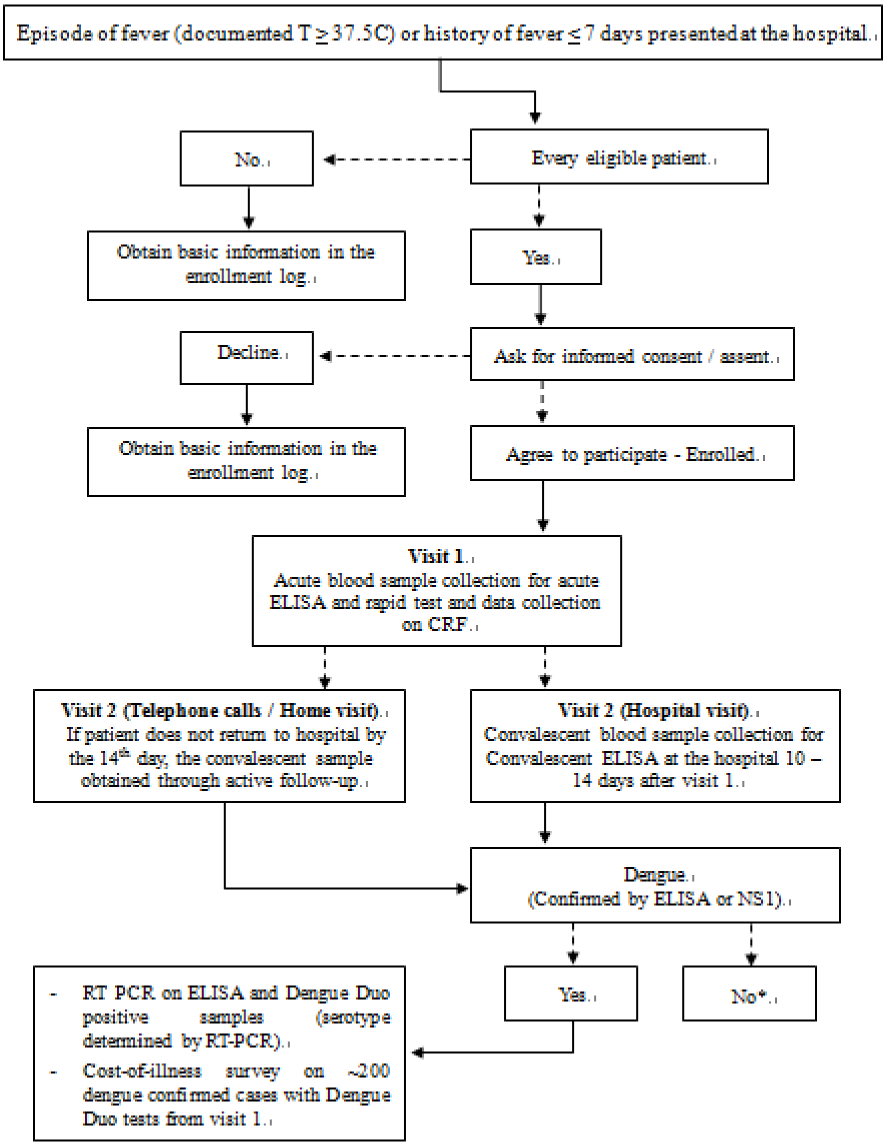

* A small number of those samples that are negative on ELISA or NS1 are tested with PCR to exclude false negative results of the ELISA.

.

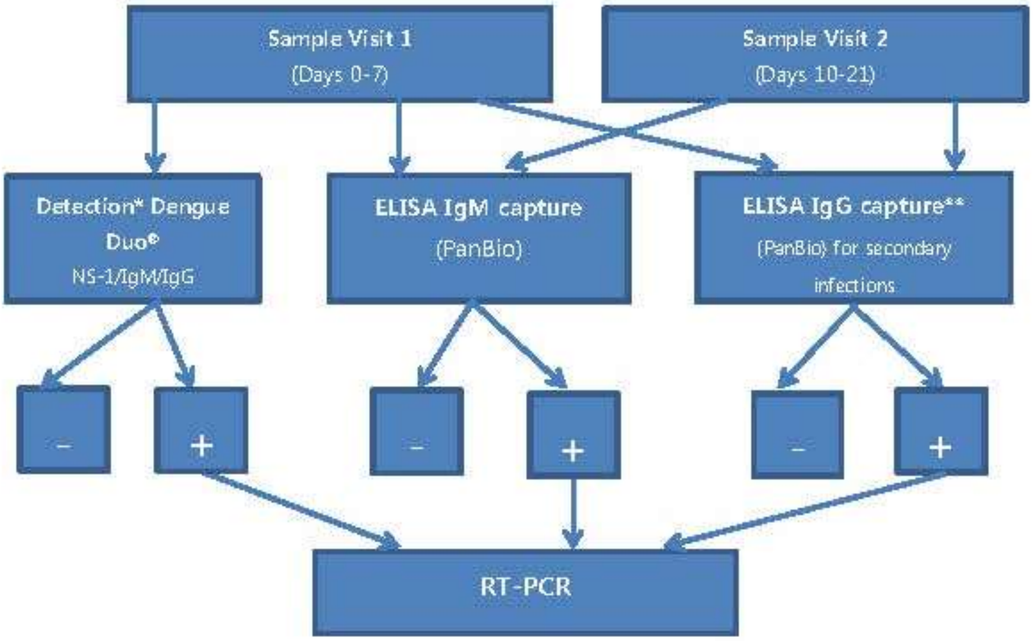

Fig. 6 Laboratory testing algorithm for dengue

* Dengue Duo® test is performed on enrolled febrile patients to identify dengue cases for immediate follow-up of dengue-confirmed cases in the cost-of-illness survey.

**Selected samples, including those that were found positive by IgM and NS1 on Dengue Duo®, as well as those positive by IgM and IgG capture ELISA, will be tested with RT-PCR.

review only

BMJ Open

Evaluating dengue burden in Africa in passive fever surveillance and sero-prevalence studies: protocol of field studies of the Dengue Vaccine Initiative

Journal:	*BMJ Open*
Manuscript ID	bmjopen-2017-017673.R1
Article Type:	Protocol
Date Submitted by the Author:	09-Aug-2017
Complete List of Authors:	Lim, Jacqueline; International Vaccine Institute, Global Dengue and Aedes-transmitted Diseases Consortium; London School of Hygiene and Tropical Medicine Faculty of Epidemiology and Population Health<br>Carabali, Mabel; International Vaccine Institute<br>Lee, Jung-Seok; International Vaccine Institute<br>Lee, Kang Sung ; International Vaccine Institute<br>Namkung, Suk; International Vaccine Institute<br>Lim, SI-Ki; International Vaccine Institute<br>Ridde, Valéry; Université de Montréal, Centre de recherche du Centre hospitalier de l'Université de Montréal (CR-CHUM); Université de Montréal, Département de médecine sociale et préventive<br>Fernandes, Jose; Hopital Albert Schweitzer, Lambaréné,, Centre de Recherches Médicales de Lambaréné<br>Lell, Bertrand; Hopital Albert Schweitzer, Lambaréné, Centre de Recherches Médicales de Lambaréné<br>Matendechero, Sultani ; Ministry of Health, Kenya, Dept. of Neglected Tropical Diseases<br>Esen, Meral; University of Tübingen, Institute of Tropical Medicine<br>Andia, Esther; Kenya Medical Research Institute<br>Oyembo, Noah; Kenya Medical Research Institute<br>Barro, Ahmed; Action-Gouvernance-Integration-Reinforcement, Program Equité<br>Bonnet, Emmanuel ; Institut de recherche pour le developpement<br>Njenga, Sammy; Kenya Medical Research Institute, Eastern & Southern Africa Centre of International Parasite Control<br>Agnandji, Selidji ; Hopital Albert Schweitzer, Lambaréné, Centre de Recherches Médicales de Lambaréné<br>Yaro, Seydou; Centre MURAZ<br>Alexander, Neal; London School of Hygiene and Tropical Medicine Faculty of Epidemiology and Population Health<br>Yoon, In Kyu; International Vaccine Institute, Global Dengue and Aedes-transmitted Diseases Consortium
**Primary Subject Heading**:	Infectious diseases
Secondary Subject Heading:	Infectious diseases, Epidemiology, Global health, Public health
Keywords:	dengue, Epidemiology < INFECTIOUS DISEASES, Public health < INFECTIOUS DISEASES, Tropical medicine < INFECTIOUS DISEASES

For peer review only

igfEvaluating dengue burden in Africa in passive fever surveillance and sero-prevalence studies: protocol of field studies of the Dengue Vaccine Initiative

Jacqueline K. Lim[1*], Mabel Carabali[1], Jung-Seok Lee[1], Kang Sung Lee[1], Suk Namkung[1], Sl-Ki Lim[1], Valery Ridde[2], Jose Fernandes[3], Bertrand Lell[3], Sultani Hadley Matendechero[4], Meral Esen[5], Esther Andia[6], Noah Oyembo[6], Ahmed Barro[7], Emmanuel Bonnet[8], Sammy M. Njenga[6], Selidji Todagbe Agnandji[3], Seydou Yaro[9], Neal Alexander[10], In Kyu Yoon[1]

[1]International Vaccine Institute, Seoul, Korea, Republic of; [2]Montreal School of Public Health (ESPUM), Montreal, QC, Canada; [3]Centre de Recherches Médicales de Lambaréné, Hôpital Albert Schweitzer, B.P.118, Lambaréné, Gabon; [4]Communicable Disease Prevention and Control, Ministry of Health, Nairobi, Kenya; [5]Institute of Tropical Medicine, University of Tübingen, Germany; [6]Eastern and Southern Africa Centre of International Parasite Control (ESACIPAC), Kenya Medical Research Institute (KEMRI), Nairobi, Kenya; [7]AGIR, Ouagadougou, Burkina Faso, [8]Institut de Recherche pour le Développement (IRD), Paris, France; [9]Centre Muraz, Bobo-Dioulasso, Burkina Faso; [10]Epidemiology and Public Health Department, London School of Hygiene and Tropical Medicine

*Corresponding author
Jacqueline K. Lim[1*], MPH
Research Scientist
Global Dengue and Aedes-transmitted Diseases Consortium (GDAC)
International Vaccine Institute (IVI)
SNU Research Park, Gwankak-ro 1
Gwanak-gu, Seoul, Korea 151-191
T: +82-2-881-1340 (Dir) / +82-2-872-2801 (ext. 340)
Email: kalim@ivi.int

36 pages
Word count of the text: 6,422 (not counting the Abstract)
Word count of the abstract: 320
Figures and tables: 6 figures and 1 table

For peer review only

Abstract

Introduction.
Dengue is an important and well-documented public health problem in the Asia-Pacific and Latin American regions. However, in Africa, information on disease burden is limited to case reports and reports of sporadic outbreaks, thus hindering the implementation of public health actions for disease control. To gather evidence on the undocumented burden of dengue in Africa, epidemiological studies with standardized methods were launched in three locations in Africa.

Methods and Analysis.
In 2014-17, the Dengue Vaccine Initiative (DVI) initiated field studies at three sites in Ouagadougou, Burkina Faso; Lambaréné, Gabon; and Mombasa, Kenya; to obtain comparable incidence data on dengue and assess its burden through standardized hospital-based surveillance and community-based serological methods. Multidisciplinary measurements of the burden of dengue were obtained through field studies that included passive facility-based fever surveillance, cost-of-illness surveys, serological surveys, and healthcare utilization surveys. All three sites conducted case detection using standardized procedures with uniform laboratory assays to diagnose dengue. Healthcare utilization surveys were conducted to adjust population denominators in incidence calculations for differing healthcare seeking patterns. The fever surveillance data will allow calculation of age-specific incidence rates and comparison of symptomatic presentation between dengue and non-dengue patients using multivariable logistic regression. Serological surveys assessed changes in immune status of cohorts of approximately 3,000 randomly selected residents at each site at 6 month intervals. The age-stratified serosurvey data will allow calculation of seroprevalence and force of infection of dengue. Cost-of-illness evaluations were conducted among patients with acute dengue by Rapid Diagnostic Test.

Ethics and Dissemination.
The protocol for each study obtained ethical approvals from the Institutional Review Boards of the International Vaccine Institute, the London School of Hygiene and Tropical Medicine, and the Ethics Committee of host country institutions. By standardizing methods to evaluate dengue burden across several sites in Africa, these studies will generate evidence for dengue burden in Africa and data will be disseminated as publication in peer-review journals by the end of 2017.

For peer review only

Strengths of this study

- There have not been population-based studies conducted with a multi-disciplinary approach (i.e. surveillance, healthcare utilization, and sero-survey in one catchment area population). Data from the passive surveillance will be used to calculate annual incidences of dengue and data from the serosurvey will estimate force of infection and prevalence.
- The studies were conducted in three locations in Africa, based on standardized methods and laboratory algorithm. Thus, comparison by site would be possible.

Limitations of this study

- This is not a cohort study. The passive facility-based surveillance may lead to under-estimation of the burden of dengue fever by measuring incidence based on only those that sought care at our study facilities.
- There may be limited generalizability of our study results to other dengue-endemic parts of Africa.

Keywords: dengue; Africa; seroepidemiologic Studies; incidence

Running head: Evaluating dengue burden in Africa: DVI field studies

For peer review only

Background

Dengue fever, a mosquito-borne flavivirus infection caused by four related but antigenically distinct dengue viruses (DENVs, serotypes 1–4), is a major and rapidly increasing global public health problem. Recent studies have estimated an annual incidence of 50–100 million symptomatic infections globally [1]. Dengue is a high burden disease that disproportionately affects countries in the tropics and subtropics, many of which have limited health care resources [2]. Although one dengue vaccine has been recently licensed in several endemic countries, the vaccine has restricted age and epidemiological indications. Other prevention and control measures such as vector control are suboptimal as stand-alone interventions [3 4], and no drugs for treatment are currently available.

Like in Asia and the Americas, there were reports of multiple epidemics of dengue in Africa in 1779-1780[5]. Specifically for Africa, there are records of multiple dengue case reports between 1964 and 1968 with DENV 2 in Nigeria[6]. Data from several studies conducted in the 1960-70s in Nigeria supported a substantially high level of immunity in adults as well as children [7 8]. In 2011, Amarasinghe et al. conducted a comprehensive review of literature on dengue in Africa and described that dengue cases have been reported in 34 countries in Africa, with most of these countries also having *Aedes* mosquitoes [9]. However, prior studies which suggested the presence of dengue in Africa were limited by their retrospective design or sample collection (blood donors or sample collected from surveys of other diseases), and often from travellers, with a small number of reported autochthonous cases, to demonstrate the true, population-based, burden of dengue. Also, while many dengue endemic countries in Asia and Latin America have mandatory reporting of dengue cases to public health authorities, and national surveillance systems in place to monitor incidence patterns [10], most African countries lack such established reporting mechanisms and only

For peer review only

sporadic outbreaks and individual case reports have been documented. In addition, the frequently non-specific clinical presentation of dengue may be difficult to distinguish from the myriad other infectious diseases present in Africa, since dengue diagnostic assays are not widely available. Thus, the burden of dengue remains largely unknown in Africa [9 11]. Without such dengue burden data, informed decision-making about prevention and control measures, including dengue vaccine introduction, in Africa are not possible.

Limited by surveillance capacity hindering continuous reporting in the region, there had not been frequent and systematic reporting of dengue in Africa. African ancestry is known to be protective against severe dengue and the candidate genes were recently identified in Cuban patient [12 13]. Bhatt et al.'s modelling of global dengue burden suggests high burden of dengue in Africa in terms of equal numbers of, both apparent and inapparent, infections as that of Latin America[1]. There are new findings about dengue in Africa, but there is still much unknown about the magnitude of dengue problem in the continent. To improve estimates of population-based dengue disease burden in Africa and validate whether the undocumented burden of dengue is as high in Africa as in the Americas with empirical data, the Dengue Vaccine Initiative (DVI) initiated field studies at three sites in West, West-Central, and East Africa in Ouagadougou, Burkina Faso; Lambaréné, Gabon; and Mombasa, Kenya; respectively. In each of the three sites, a standardized package of study components, including passive facility-based fever surveillance, healthcare utilization surveys, cost-of-illness surveys, and serological surveys (Fig 1), was initiated between December 2014 and March 2016.

Methods

Site selection

Study sites were selected, in part, based on their likelihood of supporting DENV transmission. In site selection, we considered dengue outbreaks and cases reports in the literature, available seroprevalence studies, as well as country-specific dengue risk maps of the probability of DENV transmission and the level of evidence of dengue presence reporting the uncertainty of the consensus estimates of dengue in Africa [6 14]. In addition, adequate research infrastructure to implement the studies was taken into account. Finally, inclusion of different regions of Africa was also a factor in site selection. Thus, Ouagadougou, Burkina Faso; Lambaréné, Gabon; and Mombasa, Kenya; were selected, respectively, to measure the true burden of dengue in selected sites from West, (West-) Central, and East Africa.

In Burkina Faso, the first reported dengue outbreak occurred in Ouagadougou in 1982 due to DENV-2 [9]. Serological prevalence of dengue was found to be 26.3% in a rural setting (Nouna village) and 36.5% in an urban setting (Ouagadougou) in 2006 [15]. More recently, an observational study conducted by Ridde et al. among febrile patients consulting at selected study facilities in 2013-14 showed 8.7 % (33/379) to be positive by dengue RDT; and 15 of 60 samples tested by RT-PCR to be dengue positive [16]. With evidence for the presence of dengue, along with a strong health and demographic surveillance system (Ouaga-HDSS) which could be used to describe the demographic characteristics of the catchment area, a field study was initiated in Ouagadougou, Burkina Faso in December 2014.

In Gabon, cases of dengue hemorrhagic fever (DHF) caused by up to three different DENV serotypes have been reported, and dengue seroprevalence has been found to be between 5 and 20% [17-19]. Results of a recently published study demonstrated seroprevalence of 12.3% among toddlers approximately 30 months of age in semi-rural Lambaréné between 2007 and 2010 [20]. However, a different study in 2005-2008 suggested minimal DENV transmission in rural areas of Gabon [21]. This latter study examined antibodies against dengue in individuals from randomly selected villages representing about 10% of all Gabonese

For peer review only - http://bmjopen.bmj.com/site/about/guidelines.xhtml

villages. Blood samples were tested by anti-DENV IgG and IgM capture ELISA and found to have only minimal IgG (0.5%) and IgM (0.5%) seroprevalence. Based on these low prevalences, authors concluded that there was no active circulation of DENV in rural Gabon. However, the low seroprevalence may have been affected by low sensitivities of the tests used leading to a high rate of false negative, and/or selection bias in the blood sample pool among the selected villagers [22]. Seroprevalence estimates may have also been impacted by the possibility of false-positive results due to IgG cross-reactivity among flaviviruses [21]. Nevertheless, given the possibility of DENV circulation in Gabon, a field study was initiated in Lambaréné in March 2015 in a community with a catchment population of about 77,000 residents, using the clinical research infrastructure of the Centre de Recherches Medicales de Lambaréné (CERMEL), benefiting from experienced research staff who conducted a large Phase 3 malaria vaccine trial [23 24].

In Kenya, more evidence is available for the presence of dengue based on local data. Dengue was the most common viral pathogen in retrospectively tested blood specimens from HIV-negative survey samples from the 2007 Kenya AIDS Indicator Survey. Antibody testing for dengue as well as chikungunya and Rift Valley fever was performed by IgG ELISA using either commercial kits or CDC assays; 12.5% were found to be dengue positive [25]. Similarly, a household survey found 13% of individuals from 701 households in Mombasa had serological evidence of either past or current DENV infection [26]. These data suggest that there is more dengue in Kenya than indicated by public health reporting, possibly due to misdiagnosis [25 26]. A field study was initiated in Mombasa, Kenya in March 2016.

Study participants

For the passive facility-based fever surveillance, individuals who met the following criteria were eligible for study enrollment:

For peer review only

1. Age 1- 55 years old;
2. Resident of the catchment area covered by healthcare facilities participating in the study, without plans to move out of the catchment area within 12 months;
3. Signed informed consent, and assent for those aged between 7 and 18 years; and
4. Patients presenting with current fever (axillary temperature ≥ 37.5º C) or history of fever for ≤ 7 days duration without localizing signs (fever caused by a localized infection as well as fever with a known and confirmed etiology other than dengue, such as malaria confirmed by malaria RDT- listed in the patient identification SOP).

For the serological survey, criteria 1-3 were applied. For the healthcare utilization survey, household interviews were conducted among the heads or representatives of the household invited from each family participating in the serosurvey.

Study area and population

Burkina Faso, located in West Africa, has a population of 14,017,462 with 22.7% living in urban areas. The country is mainly rural with about 29% of the population reported to be living in urban areas in 2014. However, Burkina Faso is urbanizing rapidly and is positioned as the country with the fourth fastest urbanization in the last 25 years [27 28]. The capital, Ouagadougou, has a population of 2,741,128. The majority of the population live in urban settings. About 45% of the population are under 15 years of age [29]. The city is divided into 12 districts and 52 sectors. Ouagadougou is the country's largest city and the cultural and economic center. The city is part of the Soudano-Sahelian area, with a rainfall of about 800 mm per year. The rainy season is from May to October, with a mean temperature of 28 °C (82 °F). The cold season runs from December to January, with a minimum average

For peer review only

temperature of 16 °C (61 °F). During the hot season, which runs from March to May, the temperature can reach as high as 43 °C (109 °F).

The Health and Demographic Surveillance System is in place in Ouagadougou. Ouaga HDSS monitors a population of 81,717 residents; according to this surveillance system, the city population is very stable with a rate of migration of 4.1% and more than 80% of the inhabitants with ownership of their houses [20]. A map of the city and distribution of the population is shown in Figure 2.

Gabon, located on the west coast of Central Africa, has an area of nearly 270,000 square kilometres (100,000 sq. mi) with a population estimated at 1.5 million. Its capital and largest city is Libreville. In 2014, it is reported that 87% of the Gabonese population lived in urban areas [28]. The sixth largest city, Lambaréné, the capital of Moyen-Ogooué, is located 75 kilometers south of the equator, with a population of 25,257 in 2009. The majority of Lambaréné residents live in semi-rural areas. About 42% of the Gabonese population is under 15 years of age [29]. Similarly, Lambaréné's population is relatively young with about 50% under 20 years of age.

The health services of Gabon are mostly public, but there are some private institutions as well. With one of the best medical infrastructure in the region, almost 90% of the population have access to health care services. Albert Schweitzer Hospital (ASH) is a private institution which served as a study site for the passive fever surveillance study [30 31]. The study area in Lambaréné is shown in Figure 3.

Kenya, located in East Africa, lies on the equator, covering 581,309 km^2 (224,445 sq. mi), with a population of approximately 45 million people in 2014 [2]. Kenya generally has a warm and humid tropical climate, but is diverse ranging from the cooler climate around the capital city, Nairobi, to a hot and dry climate inland, as well as a desert-

For peer review only

like climate in the north-eastern regions along the border with Somalia and Ethiopia [32]. The capital, Nairobi, is a regional commercial hub. The main industries include agriculture, exporting tea and coffee, as well as the service industry.

Kenya is divided into 47 semi-autonomous counties. Mombasa is the country's second largest city after Nairobi and is located on the east coast of the country [2]. Administratively, Mombasa is the capital of Mombasa County, what was formerly called Coast Province. This overall Coast region covers over 80,000 km^2 in the south-eastern part of Kenya, constituting about 15% of the country's land area, with a population of 3,325,307 residents.

The main driver of economy of Mombasa is tourism and trading industry. Mombasa itself has a population of about 1.3 million with almost 50% of the population under 15 years of age [29]. Increasingly, the population of the province lives in urban areas; at present about 45% live in Mombasa and other urban centers. The long rains begins around April and the short rains begins in October [32]. Mean annual temperature ranges from 24°C to 27°C, but maximum temperature averages over 30°C during the hottest months, January to April.

Figure 4 shows the area of Mvita subcounty of Mombasa, which was the catchment area for the study in Kenya, with a catchment population of 74,735 residents. The map indicates the three facilities involved in the study.

Sample size

Given the paucity of available age-specific dengue incidence data in the study countries or nearby countries, it was difficult to obtain population-based incidence to make assumptions when calculating sample sizes. The required catchment population for the passive facility-based fever surveillance was roughly estimated based on the limited data available in the literature. Annual incidence estimates were calculated based on available

For peer review only

prevalence estimates with the assumption that the outcome of interest has zero prevalence at age zero, and that force of infection is constant. It was assumed that prevalence estimates found for one particular age group would be adjusted as the annual incidence and used across all ages.

Wichmann et al. calculated an expansion factor for children by comparing data from three cohort studies to national surveillance data in Southeast Asia [33]. For children in Thailand, the age-specific expansion factors calculated were 11.85 for <5 years, 8.76 for 5-9 years, and 7.81 for 10-14 years [33]. The results show that, even for Asia where better reporting and surveillance systems are available, there is a considerable degree of underreporting. For Africa, such information on extent of under-reporting of dengue was not available. Also, the estimates used in our sample size calculations were not from population-based studies. While it would have been ideal to adjust the incidence further for likely underestimation, the annual incidence used in sample size calculations could not be adjusted for possible under-reporting due to the lack of data. The sample sizes were calculated with 95% confidence levels and a margin of error at a fixed significance level within 25% of the true proportion of incidence. This gives relative precision of 75%, considering the gap in evidence for dengue incidence in the study areas. The final sample sizes were calculated by assuming 20% non-response rate or loss to follow-up. The required catchment population size for the fever surveillance study in Burkina Faso was estimated to be 100,000, Gabon to be 77,000, and Kenya to be 70,000. In these catchment populations, the number of enrolled subjects depends on the number of eligible patients who seek care at the study facilities. How many eligible febrile episodes would actually present at our study facilities was difficult to predict; but after assessment of the volume of febrile patients at the facilities, a realistic upper limit for enrollment for a study period of approximately 1.5 years was set at 3,000 subjects to offer enrollment to all consenting eligible patients.

For peer review only

For the serological survey, the sample size was calculated similarly using the prevalence proportion based on published literature. Seroprevalence of 0.304 for Burkina Faso [15], 0.123 for Gabon [21], and 0.144 for Kenya [34] were used. With the same confidence levels and allowed margin of error, and assuming 10-30% (variable by site) non-response rate, the sample size was calculated to be 3,000 participants at each site. Again, with the scarcity of data from the selected countries, there were no other prevalence estimates reported or estimates from different age groups. As prevalence is expected to increase with age, and higher prevalence would give a smaller sample size, our calculations are likely to be conservative.

Study components

Fever surveillance – design and methods

To determine burden due to symptomatic dengue in each of the three sites in Burkina Faso, Gabon, and Kenya, passive facility-based fever surveillance was implemented in a well-defined catchment area population. In Burkina Faso, the surveillance study was initiated in December 2014 in five selected primary health care centres, locally called "Centre de Santé et de Promotion Sociale" (CSPS), in the municipality of Ouagadougou, with a catchment population of 105,000 residents. This project was implemented in collaboration with Centre Muraz in Bobo-Dioulasso, EQUITE sante program (a collaborative program between University of Montreal and Action-Gouvernance-Integration-Reinforcement, AGIR, based in Ouagadougou, funded by Canadian Institute of Health Research), and DVI. In Gabon, the surveillance study was initiated in the Albert Schweitzer Hospital serving a catchment population of 130,000 residents in the Moyen-Ogooué and surroundings within Lambaréné, in collaboration with CERMEL and Institute of Tropical Medicine in Tubingen,

For peer review only

Germany. In Kenya, the surveillance study was implemented at Ganjoni dispensary, Tudor sub-county Hospital, and Coast Provincial General Hospital, serving a catchment population of 70,000 residents in Mombasa, in collaboration with Kenya Medical Research Institute (KEMRI) and Ministry of Health of Kenya.

As described in Figure 5, both outpatients and inpatients at the designated study facilities, who meet inclusion criteria as mentioned earlier are tested for dengue, first with SD Dengue Duo® RDT. Dengue confirmation is done by detection of dengue virus in serum samples using PCR, as well as anti-dengue IgM and IgG antibodies in acute and convalescent serum by ELISA (SD Dengue IgM & IgG capture ELISA® tests, Standard Diagnostics, Yongin-Si, Korea) [35 36]. Every consecutive patient meeting inclusion criteria is eligible for enrolment during the study period. Infants < 1 year old were not included due to operational limitations, such as difficulty of infantile bleeding.

In Ouagadougou, Burkina Faso, the fever surveillance initiated in December 2014 continued until February 2017 (approximately 2 years). In Lambaréné, Gabon, the fever surveillance initiated in April 2015 continued until January 2017 (approximately 1.5 years). In Mombasa, Kenya, the fever surveillance initiated in March 2016 will continue until June 2017.

Among subjects enrolled in the fever surveillance, those who are positive by dengue rapid diagnostic test are offered further enrolment in the cost-of-illness survey, consisting of interviews on the day of acute illness visit, day 10-14 from the first visit, and day 28, if illness continues. The cost-of-illness survey questionnaire was designed to estimate the direct medical, direct non-medical, and indirect costs associated with dengue-positive patients identified at study facilities. This survey also estimates the cost of treating dengue at the

For peer review only

facility level. Data are gathered by linking patients' medical records concerning outpatient visits, inpatient visits, and service consumption (e.g., diagnostic tests, medication, and other services provided to patients). The cost-of-illness portion of the study will be described separately.

Fever surveillance – laboratory testing

As shown in Figure. 6, in all three sites, acute samples are tested using a commercial RDT for dengue NS1 and IgM/IgG (Dengue Duo®, Standard Diagnostics, Yongin-Si, Korea). Dengue Duo RDT is used on the day of acute illness visit at the site of patient presentation (day 1). The acute and convalescent samples are subsequently tested at a local laboratory using dengue IgM/IgG ELISA (SD Dengue IgM & IgG Capture ELISA®, Standard Diagnostics, Yongin-Si, Korea). The serum is separated and stored in 4 aliquots of about 500 μL for various lab tests, as indicated in consent documents.

After ELISA testing, samples will be shipped to IVI in Korea. Samples with positive results by RDT or ELISA undergo further testing by RT-PCR at the Clinical Immunology Laboratory of IVI. Four DENV serotype-specific real-time RT-PCR assays are used for laboratory confirmation of dengue and serotyping [37]. The DENV 1-4 RT-PCR assays are carried out in 25μL reaction mixtures containing 5μL template RNA, TagMan® Fast Virus 1-step mastermix (Applied Biosystems®), 0.9 μM of each primer, and 0.2 μM probe [37].

Amplification and detection are performed in a StepOne Plus real-time PCR system and the baseline and threshold are determined using the auto-baseline and threshold feature in StepOne Software v2.2.2 (Applied Biosystems®). Thermocycling parameters are as follows: reverse transcription at 50 °C for 5 min, inactivation at 95 °C for 20 s, followed by 45 cycles

For peer review only - http://bmjopen.bmj.com/site/about/guidelines.xhtml

of fluorescence detection at 95 °C for 3 s, and annealing at 60 °C for 30 s [37]. A specimen is considered positive if target amplification was recorded within 40 cycles.

Serological survey – design and methods

While the facility-based fever surveillance studies provide estimates of the burden of medically-attended dengue disease, evaluation of all DENV infections in a population – including subclinical and mildly symptomatic infections, which impact immune status – is needed to capture the overall impact of dengue. As part of the study package, population-based serological surveys were conducted in the same catchment population used for the fever surveillance. At each of the three sites in Africa, the serosurvey was conducted on a cohort of approximately 3,000 randomly selected residents of urban and semi-urban parts of Ouagadougou, Lambaréné, and Mombasa. Without individual-level census information on all residents of Lambaréné and Mombasa, with help of community/village health workers, randomization was done based on neighbourhoods (or defined areas for which the health workers/volunteers are responsible) as cluster units. As the community/village health workers are familiar with the villages and their residents, they are good entry points into the communities. With these health workers, the field team screened houses in the selected villages by knocking on doors of every 5~7 houses, depending on the household density per neighbourhood. Also, demographic information collected in previous research projects conducted in the same area was used as a guide, if available. In the case of the site in Ouagadougou, HDSS data were available and the EQUITE SANTE, a CIHR funded research program of University of Montreal had set up a geographic information system (GIS) database system of houses in the study area. Using these data, households of potential enrollees of the serosurvey were pre-selected randomly and household visits were made in

For peer review only

Ouagadougou. In the three sites, about 45% of the serosurvey samples were targeted to be collected from children 1 - 14 years-of-age, and 55% were targeted to be collected from adults between 15 and 55 years of age to reflect the age distribution of the general population of the area. Household-based enrollment was offered to the head of the household until the specific cap for the age-group was reached in Lambaréné and Mombasa.

Randomly-selected subjects 1-55 years of age underwent phlebotomy (5ml for children and 7ml for adults) twice — during pre-transmission (before the rainy season) and post-transmission (after the rainy season) at 6 month intervals. The sera were evaluated using IgG indirect ELISA at baseline and after 6 months. The presence of dengue IgG antibodies at 6 month intervals will be used to estimate the level of occurrence of inapparent DENV infection and to calculate the rate of infection in the catchment population. Flow cytometry-based DENV neutralization assays will be applied to a subset of samples to assess for presence of dengue neutralizing antibodies and seroconversion over the 6 month interval. In addition to overall seroconversion, age-specific seroconversion estimates in the catchment population, as well as the proportion of inapparent infections, are determined.

Serological survey – laboratory testing

From the samples collected in the serosurvey, about 200 μL of serum are used and tested at a local laboratory using dengue IgG ELISA (Panbio Dengue IgG Indirect ELISA®, Alere North America, LLC, Florida, United States). After ELISA testing for dengue IgG at the local labs, samples will be shipped to IVI. Given potential serological cross-reactivity among flaviviruses [38], flow cytometry-based neutralization assays will be performed against selected flaviviruses to include yellow fever virus, West Nile virus, Zika virus, and Japanese Encephalitis virus at the Clinical Immunology Lab of the International Vaccine Institute (IVI),

For peer review only

Seoul, Korea [39 40]. About 50 samples per bleed for 4 bleeds in Burkina Faso and 2 bleeds in Gabon and Kenya will be tested.

About 1,000 µL of serum is kept aside for this procedure. The flow cytometry-based neutralization assays are performed in duplicate in 96-well cell culture plates with flat-bottom wells, each containing DC-SIGN-expressing U937 cells [39]. The amount of virus used in the assay infects between 7 and 15% of the cells. Human immune sera are serially diluted and the virus is pre-incubated with the sera for 1 h at 37°C [39]. The cells are washed, and the virus and serum mixture is added to the cells for 1 h at 37°C, and the cells are further incubated for 24 to 48 h at 37°C in 5% CO_2. The cells are fixed, permeabilized, and stained with fluoresce-conjugated monoclonal antibody 4G2, which recognizes the flavivirus E protein [41]. FACScan flow cytometer (Becton Dickinson, San Diego, CA) is used to analyze the cells [39]. The serum dilution that neutralized 50% of the viruses is calculated by nonlinear, dose-response regression analysis with Prism 4.0 software (GraphPad Software, Inc., San Diego, CA).

In addition, a Luminex-based multiplex immunoassay will be performed on a randomly selected sub-sample to assess for IgG to different flaviviruses [42]. About 200 samples per bleed for 4 bleeds in Burkina Faso and 2 bleeds in Gabon will be tested. Detection of IgG against ZIKV and each the four DENV serotypes was performed on patient serum samples using an in-house microsphere-based multiplex immuno-assay (arbo-MIA) at the Clinical Immunology Lab of IVI [43 44]. The arbo-MIA is based on a mixture of microspheres covalently coupled with either DENV-1, -2, -3, -4 or ZIKV recombinant antigens (E protein domain III) produced in Drosophila S2 expression system. Briefly, microsphere mixtures were sequentially incubated in the dark under constant shaking with a 1:400 dilution of patient serum samples, with 2 µg/mL anti-human IgG biotin-conjugated antibody (Jackson Immunoresearch, West Grove, PA) and with 2 µg/mL streptavidin-R-phycoerythrin conjugate (Life technologies). After the final incubation, the median

For peer review only

fluorescence intensity (MFI) of each microsphere set was quantified using a BioPlex 200 instrument (Bio-Rad Laboratories, Hercules, CA). Samples were considered seropositive if the ratio of MFI values obtained for the viral antigen to the control antigen was superior to the defined cut-off. The cut-off of the MIA was determined for each viral antigen by ROC curve analysis using well characterized sera.

In Lambaréné, the enrolment bleed, took place in November -December 2015 with 2nd blood collection in May 2016. In Ouagadougou, the enrolment bleed took place in May-June 2015 with follow-up blood collections in December 2015, June 2016, and January 2017. In Mombasa, enrolment bleed took place in May 2016 with the 2nd blood collection in November 2016 – February 2017.

Healthcare Utilization Survey

As the passive fever surveillance is conducted at the study facilities, potential dengue patients could be missed if they seek care elsewhere. To identify the proportion of fever and dengue cases potentially missed by the passive surveillance system due to patients living in the study area but seeking care outside of study facilities, a population-based healthcare utilization survey was conducted in 400 randomly selected households from the study catchment area to characterize their healthcare utilization patterns of the households when they have (self-reported) febrile episodes among the family members. In addition to assessing health-seeking behaviours of the residents, preferences in terms of health-seeking behavior and respective reasons for their preferences were investigated. The questionnaire was administered to 400 heads of households. Among 3,000 residents who participated in the serosurvey, there were about 600 households. From these households, 400 heads of households were randomly selected and offered enrolment in the health utilization survey.

For peer review only

Heads of households or a senior representative within the household were asked questions on health seeking patterns of their family members. The surveys provide data to determine the proportion of these cases missed by our passive fever surveillance system. Also, the surveys document health seeking patterns, who would seek care at which facilities, who would make health-seeking related decisions, and the reasons for their preferences.

Study questionnaires

For the fever surveillance study, questionnaires are administered at the acute illness visit and the convalescent visit. The convalescent visit may take place at the health care facility (10-14 days later) or at the patient's home (15-21 days after the acute visit), according to patient preference and availability. The questionnaires are completed by medical staff of the study facilities, including demographic and clinical information (e.g., signs, symptoms, past medical history, treatments prescribed, and diagnoses). The same staff also complete the follow-up questionnaire at the convalescent visit within 21 days from the acute visit. Study nurses complete surveillance enrolment log. Lab technicians complete the lab section (mostly dengue-related diagnostics) and the forms are compiled by the study coordinator on site.

For the serosurvey component, questionnaires are administered at the household by trained field team staff at each serosurvey visit. Study nurses complete the questionnaire after a brief physical and medical examination. There are (a) follow-up visit(s) in about 6 months and the same staff make the household visits to complete the follow-up questionnaire. Enrolment log is maintained by our study coordinator on site.

Variables of the surveillance questionnaires

The variables collected are listed in table 1.

For peer review only

Table 1. List of variables collected in the passive fever surveillance data collection form

Topic	Description	Items
Basic information	Demographic and basic information about the patient and the treatment received	Type of treatment where patient is enrolled (IPD vs. OPD)<br>Date of fever onset, duration of fever<br>Current temperature<br>Tourniquet test results<br>Patient's address (district and village-level)<br>Date of visit, date of birth, age, and sex<br>Weight and height
General health condition	Current condition of the patient (self-report) and underlying diseases of the patient	How well the patient could handle daily activities<br>Pre-existing conditions
Signs and symptoms during this illness	A set of sign and symptoms that may be related to fever and dengue (DF and DHF)	Rash, fatigue, headache, retro-orbital pain, neck/ear pain, sore throat, breathing difficulty, cough, expectoration, gastrointestinal signs (Nausea/vomiting, diarrhea, abdominal pain, etc.), hemorrhagic signs (nose/gum bleeding, ecchymosis, petechiae, etc.), signs of shock (cyanosis, capillary refill), arthralgia, myalgia, loss of appetite, jaundice, etc.
Medical History:	Previous dengue-related or other flavivirus infection as well as vaccination history (self-report)	Previous dengue infection and related hospitalization<br>Previous infection to other commonly circulating arboviral infection in the area<br>Yellow fever vaccination history
Laboratory findings	Records from the routine laboratory tests widely used in clinical fever/dengue patient management, as part of the hospital care procedure	Platelet count, hematocrit, haemoglobin, leukocytes, neutrophils, protein level, AST, ALT, urine test results, etc.
Clinical Diagnosis	Clinician's diagnosis with or without referring to the RDT	Diagnosis given by the physician based on clinical presentation after physical examination of the patient.
Dengue testing results	Results from the dengue tests, mainly RDTs for dengue as well as other commonly circulating arbovirus in the area	Dates of blood draw<br>Test results of the RDT<br>IgM/IgG capture ELISA results<br>PCR results (if available)
Treatment	Medicine(s) prescribed and the starting and end dates	Antibiotics, paracetamol, ibuprofen, aspirin, and others that may be site-specifically prescribed
Outcome	Outcome of this particular visit	Hospitalized, returned home, or

For peer review only

		referral
Hospitalization	Information collected only among hospitalized patients in the surveillance to record other severe signs and progression of illness	Admission and discharge diagnoses<br>Presence of haemorrhagic signs or shock syndrome
Hospital Charges	Expenses and hospital charges incurred by patient on the visit 1	Amount of the out of pocket payment by the patient or the family/or guardian<br>Breakdown of the hospital charges (laboratory, medication, admission-related charges)
Final outcome	Outcome of the patient's illness at the 2nd visit	Final diagnosis given for the patient outcome of illness<br>Completion of study participation (early termination and the reason, etc.)

Planned statistical analysis

From the fever surveillance data, incidence of symptomatic dengue among patients that seek health care at the study facilities will be calculated. Age-specific incidence rates in all the children and adults will be determined by referring to the size and distribution of the general population of the study area at the time of surveillance as the denominator in calculation of the incidence of symptomatic dengue cases. Each person residing in the study area is assumed to contribute 12 months of person time to the denominator. Although the study areas all report a low migration rate, the in-migration is assumed to balance the out-migration of the population during the study period. Age-specific incidence of symptomatic dengue will be calculated by using age-specific denominators and the number of symptomatic dengue cases in eligible individuals as the numerator.

Using the data collected in the Healthcare Utilization Survey, the proportion of febrile cases missed by the passive surveillance system will be determined. Then using the proportion, the numerator will be further adjusted in recognition of those missed fever cases

For peer review only

from the study area, which could have been dengue. Also, comparison will be made between those that agreed to participate and those that declined participation among the eligible potential enrollees. The enrollment log, which recorded basic information obtained during the screening process of potential enrollees, will be reviewed. In addition to checking that our sample of febrile cases is representative of febrile patients of the general population in the catchment area, refusal rates will be determined based on information in the log. Then, the refusal rates will be used to adjust the numerator.

SPSS software will be used for analysis of the fever surveillance data. Multivariable logistic regression will be used to compare confirmed dengue patients versus non-dengue febrile patients in terms of symptomatic presentation, based on signs and symptoms collected from all patients with laboratory-confirmed dengue by serology and RT-PCR, adjusting for possible confounders, such as age, days since onset of fever, primary vs. secondary infection, inpatient vs. outpatient, etc. Differences in symptomatic complex of DF (and DHF, if data allows) by age and serotype will be also determined using multivariable logistic regression.

As outpatient disease accounts for the greater part of dengue disease burden, clinical profile of individuals with DENV infection will be characterized by the type of treatment (hospitalized and outpatients), as well as by severity of the disease (severe vs. non-severe by the 2009 WHO criteria) [45]. Classification is determined after the course of illness is completed (typically during the convalescent visit). Symptomatic dengue is classified as outpatient or hospitalized. Progression of dengue is recorded in the CRF as DF, DHF I, DHF II, DHF III or DHF IV and clinical patterns will be compared by the severity grade [45 46]. These will be compared to results obtained from other DVI studies in Latin America (Colombia) and Asia (Thailand, Vietnam, and Cambodia). Overall, comparisons will be made across Burkina Faso, Gabon, and Kenya.

With the age-stratified sera that reflect the age distribution of the general population

For peer review only

of the country, the serological survey sampling strategy ensures sufficient subjects to obtain precise age-specific estimates of sero-positivity and sero-conversion of the catchment area population. The sero-conversion rate and change in the immune status will be determined by age group during the study period. The age-stratified serosurvey data will also allow calculation of the force of infection of dengue in the study population. After enrolment, there were subjects who drop out in the follow-up bleeds about 6 months later. Basic demographic information will be compared between those that completed participation and those with incomplete participation to check whether our sample represents the catchment area population. Comparisons will be made among Burkina Faso, Gabon and Kenya.

Ethical considerations

To minimize inconvenience of the study to patients, clinicians and nurses were sensitized and trained regarding the study requirements and procedures in order for data collection to be integrated into routine patient care. The clinicians and nurses selected for the study receive coordinated support from study field staff throughout the study process. Written informed consent, and assent for participants 7 (13 for Kenya) -17 years of age, were obtained from patients by study staff. Study staff go through consent and assent documents for short summary of the disease, detailed description of study procedure, and information on reimbursement. Patient data are documented in the study designated office and only the study staff have access to the data that had been de-identified without any personal identifiable information. Data are exclusively handled in the study office and stored safely in a protected database in the study office as well as the DVI main server.

The protocol for each study obtained ethical approvals from the Institutional Review Boards (IRBs) of the International Vaccine Institute, the London School of Hygiene and

For peer review only

Tropical Medicine, and the Ethics Committee of host country institutions, including KEMRI Scientific and Ethical Review Unit in Kenya, Gabon National Ethics Committee and Institutional Ethics Committee, Scientific Review Board of CERMEL in Gabon, and the IRB of Centre Hospitalier de l'Université de Montréal (CRCHUM) at University of Montreal, and the National Health Ethical Committee of Burkina Faso.

Discussion

Dengue cases have been detected since 1960's in Africa and there has been continued presence of the *Aedes* vectors in the continent [5 6]. However, very few dengue studies have been conducted in Africa. Also, little evidence is from population-based studies [9]. Compared to the volume of evidence from SE Asia and the Americas, there is critical data scarcity on dengue in Africa. Suspicion of substantial dengue burden in Africa is based on limited reports of outbreaks and a handful of sero-prevalence studies testing different viruses among samples that likely do not represent the general population. In the three countries selected for our field studies, somewhat more data were available but were still very limited. In Burkina Faso, a recent observational study conducted in 2013 reported that 8.7% of the febrile patients showed positive results on dengue RDT [16]. In Gabon, one study suggested minimal DENV circulation in rural areas [21], while a recent study reported 12.3% seroprevalence, by IgG antibodies against dengue, among toddlers 30 months of age in semi-rural parts of Lambaréné [20]. In Kenya, about 13% of the individuals in Mombasa have been reported evidence of past or current DENV infection by RT-PCR and IgM anti-dengue ELISA after the 2013 outbreak [26]. Despite the limited scope and generalizability of these studies, they suggest that there may be more dengue than previously appreciated due to underestimation and misdiagnosis [25 26].

These studies all indicate presence of dengue and some level of underlying sero-

For peer review only

prevalence in the countries of our field studies. However, often these studies are limited by their retrospective design or sample collection (blood donors or sample collected from surveys of other diseases) in terms of assessing the true, population-based, burden of dengue. We proposed to address this gap by population-based dengue surveillance and sero-prevalence studies in West, (West-) Central, and East Africa.

The present studies at three sites in Africa will provide important information on undocumented DENV circulation in Africa. Such data will help to strengthen the evidence base for dengue burden in Africa. Better defined disease burden data based on our studies could be used to assess the relative need for dengue prevention and control measures, such as whether a dengue vaccine would be a cost-effective public health intervention for countries in Africa. Clinical findings from our studies could also be used as a guide for dengue case detection and case management.

The studies have some important limitations. One potential source of bias in estimating the incidence of symptomatic dengue is under-ascertainment due to the community residents with relevant symptoms seeking care from other healthcare providers and facilities than the facility under surveillance. As the study design remains passive surveillance, cases are ascertained only at our study facilities. By estimating the proportion of febrile patients seeking care elsewhere, as well as refusal rates among the potential enrollees that were screened for eligibility criteria, the degree of fever patients missed by the study facility will be determined. Inverse probability weighting will be used to account for these potential subjects missed by the surveillance as adjustments in incidence calculation. Also, depending on the transmission volume of dengue or other co-circulating diseases with onset of fever there may be patients that are diagnosed with other diseases and ruled out of dengue. These factors may increase the likelihood of under-reporting or over-reporting.

For peer review only

In addition, the sero-survey and healthcare utilization survey are conducted on a randomized sub-sample of the catchment area population and there may be limited generalizability of the data collected from these surveys. With unknown differences among those that agree to participate and those that do not agree, the data may not be representative of the general population of the study countries.

Conclusions

Data collection continues and study closure is planned in all three sites in April – June 2017. Although not described in this paper, preliminary results indicate a substantial level of dengue incidence and prevalence in each site. The data collected from our studies will provide a more accurate assessment of the unknown dengue disease burden in Burkina Faso, Gabon, and Kenya. These data can fill a gap in undocumented burden of dengue in the region and, collectively, may be used to infer dengue burden in other areas of Western, Central, and Eastern Africa. Countries in Africa may not consider introduction of a dengue vaccine as the foremost priority in the near future due to many other competing public health problems and limited resources as a major challenge. For cost-effective implementation of public health interventions, accurate data on dengue burden from epidemiological studies would be needed for policy makers to make evidence-based decisions on control and prevention of dengue. Our studies will provide some much needed information based on population-based research to assess dengue burden in Africa.

For peer review only

List of abbreviations

GDAC - Global Dengue and *Aedes*-transmitted Diseases Consortium
IVI - International Vaccine Institute
DENV- dengue viruses
DVI - Dengue Vaccine Initiative
Ouaga-HDSS - health and demographic surveillance system
DHF - dengue hemorrhagic fever
CERMEL - Centre de Recherches Médicales de Lambaréné
ASH – Albert Schweitzer Hospital
CSPS - Centre de Santé et de Promotion Sociale
KEMRI - Kenya Medical Research Institute
SD – Standard Diagnostics
GIS = geographic information system
MFI - median fluorescence intensity
CRCHUM - Centre Hospitalier de l'Université de Montréal

For peer review only

Declarations

- Ethics approval and consent to participate

The protocol for each study obtained ethical approvals from the Institutional Review Boards (IRBs) of the International Vaccine Institute, the London School of Hygiene and Tropical Medicine, and the Ethics Committee of host country institutions, including KEMRI Scientific and Ethical Review Unit in Kenya, Gabon National Ethics Committee and Institutional Ethics Committee, Scientific Review Board of CERMEL in Gabon, and the IRB of Centre Hospitalier de l'Université de Montréal (CRCHUM) at University of Montreal, and the National Health Ethical Committee of Burkina Faso.

- Consent for publication

Not applicable

- Availability of data and material

Data sharing is not applicable to this article as no datasets were analyzed during the current study.

This manuscript does not include data from the studies described here in. This is a protocol paper. The datasets that are being generated for analysis as described in the current study are not yet publicly available as the studies are currently ongoing at the time of submission. They will be available from the corresponding author on reasonable request.

- Competing interests

The authors declare that they have no competing interests.

- Funding

The current study was supported by funds from the Bill and Melinda Gates Foundation (OPP 1053432). The funding body had no role in the design of the study and collection, analysis, and interpretation of data and in writing the manuscript.

For peer review only

- Authors' contributions

JKL designed the study, is overseeing data collection, and was a major contributor in writing the manuscript. MC co-designed the study, oversaw some parts of data collection, and supported in writing of the manuscript. JSL was a contributor in designing of the study and oversight of parts of data collection. KSL was a contributor in oversight of data collection. SN supported in data collection. SKL supported in data collection. VR supported in designing of the study and was a major contributor in finalization of the manuscript. JF was a contributor in data collection. BL was a contributor in designing of the study and data collection. SHM was a contributor in designing of the study and site establishment. ME was a contributor in designing of the study. EA supported in data collection. NO supported in data collection. AB supported in data collection. EB supported in data generation. SMN was a contributor in designing of the study and site establishment. STA was a contributor in designing of the study and site establishment. SY was a contributor in designing of the study and site establishment. NA was a major contributor in providing oversight of the data collection and finalization of the manuscript. IKY was a major contributor in designing of the study and finalization of the manuscript.
All authors read and approved the final manuscript.

- Acknowledgements

We thank the doctors and laboratory staff of CSPS of Ouagadougou, ASH, Ganjoni dispensary, Tudor sub-county Hospital, and Coast Provincial General Hospital, as well as at Centre Muraz, CERMEL, and KEMRI. We thank collaborators at AGIR, IRD France, and University of Montreal, and University of Tubingen. Lastly, we would like to thank the DVI team and statisticians and administrative staff at the International Vaccine Institute for their helpful comments during the preparation of this manuscript and support during the studies.

For peer review only

References

1. Bhatt S, Gething PW, Brady OJ, et al. The global distribution and burden of dengue. Nature 2013;**496**(7446):504-7.
2. Murray NE, Quam MB, Wilder-Smith A. Epidemiology of dengue: past, present and future prospects. Clin Epidemiol 2013;**5**:299-309.
3. Chang M, Christophel E, Gopinath D, et al. Challenges and future perspective for dengue vector control in the Western Pacific Region, Regional Analysis. Western Pacific Surveillance and Response Journal 2011;**2**(2):9-16.
4. Eisen L, Beaty B, Morrison A, et al. ProactiveVector control strategies and improved monitoring and evaluation practices for dengue prevention. J Med Entomol 2009;**46**(6):1245-55.
5. Gubler D, Clark G. Dengue/dengue hemorrhagic fever: the emergence of a global health problem. Emerg Infect Dis 1995;**1**(2):55-57. .
6. Messina JP BO, Scott TW, Zou C, Pigott DM, Duda KA, Bhatt S, Katzelnick L, Howes RE, Battle KE, Simmons CP, Hay SI. Global spread of dengue virus types: mapping the 70 year history. Trends Microbiol 2014;**22**(3):138-46.
7. Fagbami A, Monath T, Fabiyi A. Dengue virus infections in Nigeria: a survey for antibodies in monkeys and humans. Trans R Soc Trop Med Hyg 1977;**71**(1):60-5.
8. Carey D, Causey O, Reddy S, et al. Dengue viruses from febrile patients in Nigeria, 1964-68. Lancet 1971;**1**(7690):105-6.
9. Amarasinghe A KJ, Letson GW, Margolis HS. Dengue virus infection in Africa. Emerg Infect Dis 2011;**17**(8):1349-54. .
10. Beatty ME SA, Fitzsimons DW, Hanna JN, Lam SK, Vong S, Guzman MG, Mendez-Galvan JF, Halstead SB, Letson GW, Kuritsky J, Mahoney R, Margolis HS; Asia-Pacific and Americas Dengue Prevention Boards Surveillance Working Group. Best practices in dengue surveillance: a report from the Asia-Pacific and Americas Dengue Prevention Boards. PLoS Negl Trop Dis 2010;**4**(11):e890.
11. Were F. The dengue situation in Africa. Paediatrics and Internatinoal Child Health 2012;**32**(S1):18-21.
12. Sierra B, Triska P, Soares P, et al. OSBPL10, RXRA and lipid metabolism confer African-ancestry protection against dengue haemorrhagic fever in admixed Cubans. PLoS Pathog 2017;**13**(2):e1006220. .
13. Chacón-Duque J, Adhikari K, Avendaño E, et al. African genetic ancestry is associated with a protective effect on Dengue severity in colombian populations. Infect Genet Evol 2014;**27**:89-95. .
14. Brady OJ, Gething PW, Bhatt S, et al. Refining the global spatial limits of dengue virus transmission by evidence-based consensus. PLoS neglected tropical diseases 2012;**6**(8):e1760.
15. Collenberg E, Ouedraogo T, Ganame J, et al. Seroprevalence of six different viruses among pregnant women and blood donors in rural and urban Burkina Faso: A comparative analysis. J Med Virol 2006;**78**(5):683-92.
16. Ridde V, Agier I, Bonnet E, et al. Presence of three dengue serotypes in Ouagadougou (Burkina Faso): research and public health implications. Infect Dis Poverty 2016;**5**(23).
17. Caron M, Grard G, Paupy C, et al. First evidence of simultaneous circulation of three different dengue virus serotypes in Africa. PloS one 2013;**8**(10):e78030.

For peer review only

18. Leroy EM, Nkoghe D, Ollomo B, et al. Concurrent chikungunya and dengue virus infections during simultaneous outbreaks, Gabon, 2007. Emerging infectious diseases 2009;**15**(4):591-3.
19. Caron M, Paupy C, Grard G, et al. Recent introduction and rapid dissemination of Chikungunya virus and Dengue virus serotype 2 associated with human and mosquito coinfections in Gabon, central Africa. Clinical infectious diseases : an official publication of the Infectious Diseases Society of America 2012;**55**(6):e45-53.
20. Gabor J, Schwarz N, Esen M, et al. Dengue and chikungunya seroprevalence in Gabonese infants prior to major outbreaks in 2007 and 2010: A sero-epidemiological study. Travel Med Infect Dis 2016.
21. Pourrut X, Nkoghe D, Gonzalez JP, et al. No evidence of dengue virus circulation in rural Gabon. Emerging infectious diseases 2011;**17**(8):1568-9.
22. Andries A, Duong V, Ngan C, et al. Field evaluation and impact on clinical management of a rapid diagnostic kit that detects dengue NS1, IgM and IgG. PLoS Negl Trop Dis 2012;**6**(12):e1993.
23. Lell B, Agnandji S, von Glasenapp I, et al. A randomized trial assessing the safety and immunogenicity of AS01 and AS02 adjuvanted RTS,S malaria vaccine candidates in children in Gabon. PLoS One 2009;**4**(10):e7611. .
24. Agnandji ST, Lell B, Soulanoudjingar SS, et al. First results of phase 3 trial of RTS,S/AS01 malaria vaccine in African children. N Engl J Med 2011;**365**(20):1863-75. .
25. Ochieng C AP, Vittor AY, Nyoka R, Gikunju S, Wachira C, Waiboci L, Umuro M, Kim AA, Nderitu L, Juma B, Montgomery JM, Breiman RF, Fields B. Seroprevalence of Infections with Dengue, Rift Valley Fever and Chikungunya Viruses in Kenya, 2007. PLoS One 2015;**10**(7).
26. Ellis EM, Neatherlin JC, Delorey M, et al. A household serosurvey to estimate the magnitude of a dengue outbreak in Mombasa, Kenya, 2013. PLoS Negl Trop Dis 2015;**9**(4):e0003733.
27. United Nations Development Programme. Population, urban (% of population). In: Reports HD, ed.: UNDP, 2013.
28. United Nations. World Urbanization Prospects: The 2014 Revision, Highlights. In: Department of Economic and Social Affairs PD, ed. United Nations: United Nations, 2014.
29. Central Intelligence Agency. Age structure. The world factbook Central Intelligence Agency.
30. Ramharter M, Adegnika AA, Agnandji ST, et al. History and perspectives of medical research at the Albert Schweitzer Hospital in Lambaréné, Gabon. The Middle European Journal of Medicine, WIENER KLINISCHE WOCHENSCHRIFT 2007;**119**(Suppl 3):8–12.
31. Schwarz NG, Gysels M, Pell C, et al. Reasons for non-adherence to vaccination at mother and child care clinics (MCCs) in Lambaréné, Gabon. Vaccine 2009;**27**(2009):5371-75.
32. Kenya. Secondary Kenya. https://en.wikipedia.org/w/index.php?title=Kenya&oldid=737329303
33. Wichmann O YI, Vong S, Limkittikul K, Gibbons R, Mammen MP, et al. Dengue in Thailand and Cambodia: An assessment of the degree of underestimated disease burden based on reported cases. . PLoS NTD 2010;**Accepted in Feb. 2010.**
34. Mease L, Coldren R, Musila L, et al. Seroprevalence and distribution of arboviral infections among rural Kenyan adults: a cross-sectional study. Virol J 2011;**8**(371).

For peer review only

35. Beatty ME, Stone A, Fitzsimons D, et al. Best Practices in Dengue Surveillance: A Report from the Asia-Pacific and Americas Dengue Prevention Boards. PLoS NTD 2010;***Submitted***.
36. World Health Organization. Guidelines for the clinical evaluation of dengue vaccines in endemic areas. Geneva: World Health Organization, 2008.
37. Alm E, Lindegren G, Falk K, et al. One-step real-time RT-PCR assays for serotyping dengue virus in clinical samples. BMC Infect Dis 2015;**15**(493).
38. Peeling R, Artsob H, Pelegrino J, et al. Evaluation of diagnostic tests: dengue. Nature Reviews Microbiology 2010:S30-S37
39. Kraus A, Messer W, Haymore L, et al. Comparison of plaque- and flow cytometry-based methods for measuring dengue virus neutralization. J Clin Microbiol 2007;**45**(11):3777-80.
40. de Alwis R, de Silva A. Measuring antibody neutralization of dengue virus (DENV) using a flow cytometry-based technique. Methods Mol Biol 2014;**1138**:27-39.
41. Lambeth C, White L, Johnston R, et al. Flow cytometry-based assay for titrating dengue virus. J Clin Microbiol 2005;**43**(7):3267-72.
42. Cabral-Castroa M, Saramago Peraltab R, Cavalcantia M, et al. A Luminex-based single DNA fragment amplification assay as a practical tool for detecting and serotyping dengue virus. Journal of virological methods 2016;**236**:18-24.
43. Balasuriya U, Shi P, Wong S, et al. Detection of antibodies to West Nile virus in equine sera using microsphere immunoassay. J Vet Diagn Invest 2006;**18**(4):392-5.
44. Beck C, Desprès P, Paulous S, et al. A High-Performance Multiplex Immunoassay for Serodiagnosis of Flavivirus-Associated Neurological Diseases in Horses. Biomed Res Int 2015;**2015**(678084).
45. Hadinegoro SRS. The revised WHO dengue case classification: does the system need to be modified? Paediatr Int Child Health 2012;**32**(S1):33-38.
46. Guilarde AO, Turchi MD, Siqueira Jr JB, et al. Dengue and Dengue Hemorrhagic Fever among Adults: Clinical Outcomes Related to Viremia, Serotypes, and Antibody Response. The Journal of Infectious Diseases;**197**(6): 817-24. .

For peer review only

Fig. 1 Description of the study components, including passive facility-based fever surveillance, healthcare utilization surveys, cost-of-illness surveys, and serological surveys

There are two arms in the study package, composed of four parts. In the health facility-based arm of the study package, there are the passive facility-based fever surveillance and cost-of-illness survey embedded within the surveillance. In the community arm of the study, there are serological survey and healthcare utilization survey.

For peer review only

Fig. 2 Map of the study area in Ouagadougou, Burkina Faso

Fig. 3 Map of the study area in Lambaréné, Gabon

Fig. 4 Map of the study area in Mombasa, Kenya

Figures 2 – 4 show the map of the study area at each site in Burkina Faso, Gabon, and Kenya.

For peer review only

Fig. 5 Patient flow in the fever surveillance

Eligible febrile patients identified and enrolled as study subjects will follow these steps to complete participation in the passive fever surveillance.

For peer review only

Fig. 6 Laboratory testing algorithm for dengue

Samples from subjects of the passive fever surveillance would follow these steps of the testing algorithm for confirmation of dengue.

For peer review only

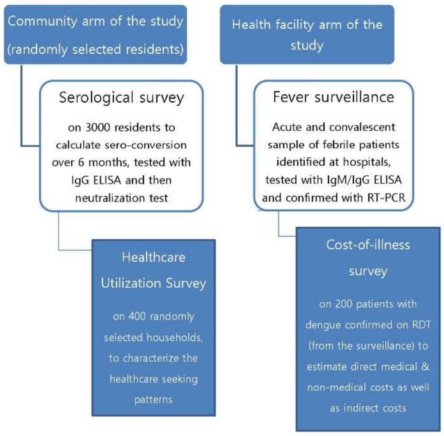

Fig. 1 Description of study components, including passive facility-based fever surveillance, healthcare utilization surveys, cost-of-illness surveys, and serological surveys

210x297mm (300 x 300 DPI)

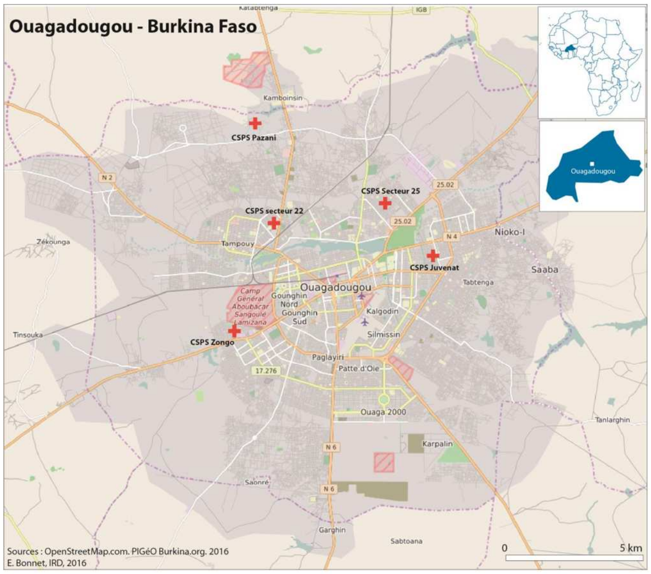

Fig. 2 Map of the study area in Ouagadougou, Burkina Faso

A map of the city and distribution of the population in Ouagadougou, Burkina Faso

140x198mm (300 x 300 DPI)

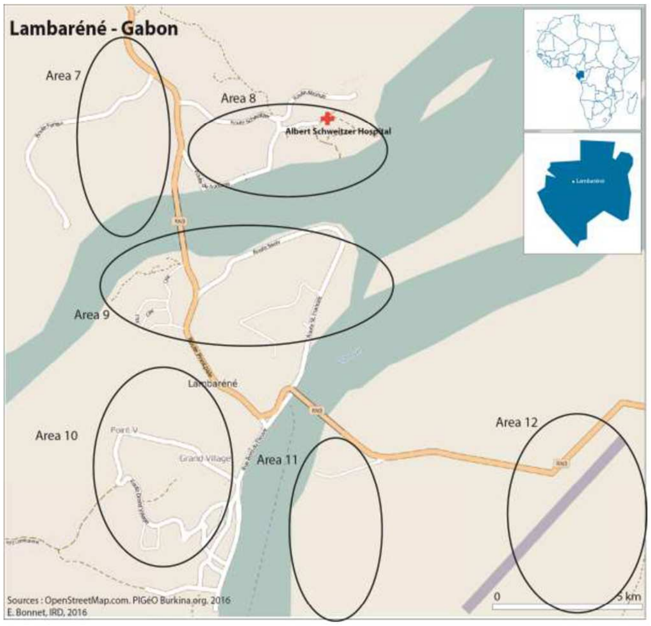

A map of the city and distribution of the population in Lambarene, Gabon

140x198mm (300 x 300 DPI)

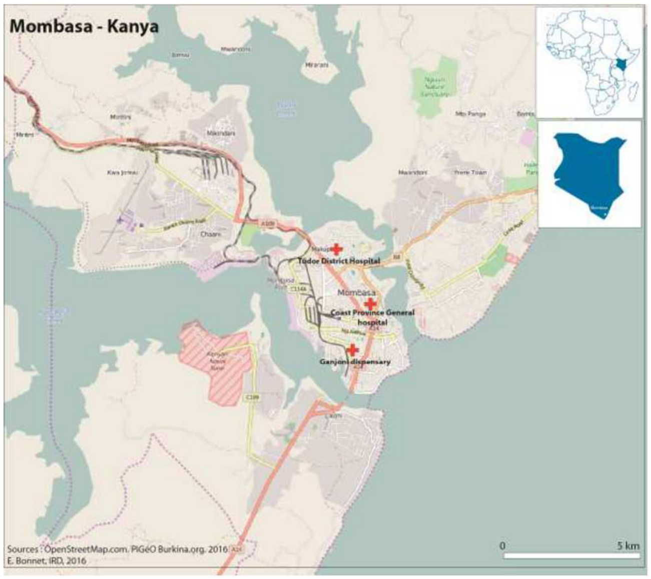

A map of the city and distribution of the population in Mombasa, Kenya

140x198mm (300 x 300 DPI)

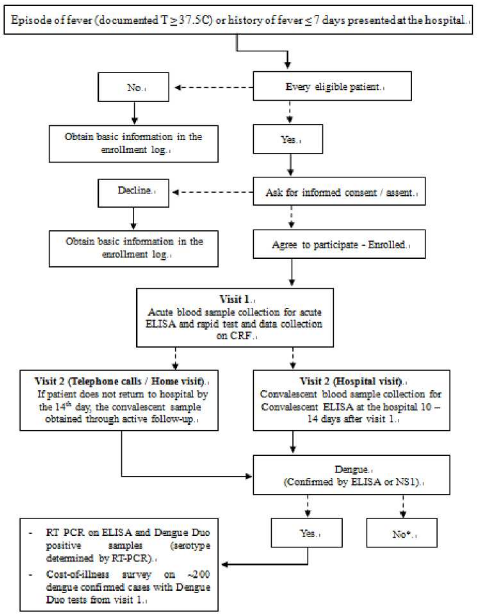

* A small number of those samples that are negative on ELISA or NS1 are tested with PCR to exclude false negative results of the ELISA.

Patient flow in the fever surveillance - Eligible febrile patients identified and enrolled as study subjects will follow these steps to complete participation in the passive fever surveillance.

140x198mm (300 x 300 DPI)

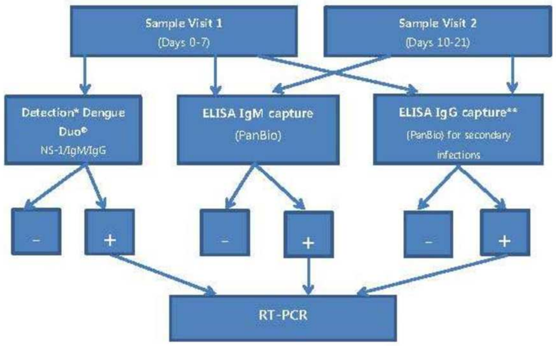

Fig. 6 Laboratory testing algorithm for dengue

* Dengue Duo® test is performed on enrolled febrile patients to identify dengue cases for immediate follow-up of dengue-confirmed cases in the cost-of-illness survey.

**Selected samples, including those that were found positive by IgM and NS1 on Dengue Duo®, as well as those positive by IgM and IgG capture ELISA, will be tested with RT-PCR.

Laboratory testing algorithm for dengue: Samples from subjects of the passive fever surveillance would follow these steps of the testing algorithm for confirmation of dengue.

140x198mm (300 x 300 DPI)

Evaluating dengue burden in Africa in passive fever surveillance and sero-prevalence studies: protocol of field studies of the Dengue Vaccine Initiative

Journal:	*BMJ Open*
Manuscript ID	bmjopen-2017-017673.R2
Article Type:	Protocol
Date Submitted by the Author:	08-Sep-2017
Complete List of Authors:	Lim, Jacqueline; International Vaccine Institute, Global Dengue and Aedes-transmitted Diseases Consortium; London School of Hygiene and Tropical Medicine Faculty of Epidemiology and Population Health<br>Carabali, Mabel; International Vaccine Institute<br>Lee, Jung-Seok; International Vaccine Institute<br>Lee, Kang Sung ; International Vaccine Institute<br>Namkung, Suk; International Vaccine Institute<br>Lim, SI-Ki; International Vaccine Institute<br>Ridde, Valéry; Université de Montréal, Centre de recherche du Centre hospitalier de l'Université de Montréal (CR-CHUM); Université de Montréal, Département de médecine sociale et préventive<br>Fernandes, Jose; Hopital Albert Schweitzer, Lambaréné,, Centre de Recherches Médicales de Lambaréné<br>Lell, Bertrand; Hopital Albert Schweitzer, Lambaréné, Centre de Recherches Médicales de Lambaréné<br>Matendechero, Sultani ; Ministry of Health, Kenya, Dept. of Neglected Tropical Diseases<br>Esen, Meral; University of Tübingen, Institute of Tropical Medicine<br>Andia, Esther; Kenya Medical Research Institute<br>Oyembo, Noah; Kenya Medical Research Institute<br>Barro, Ahmed; Action-Gouvernance-Integration-Reinforcement, Program Equité<br>Bonnet, Emmanuel ; Institut de recherche pour le developpement<br>Njenga, Sammy; Kenya Medical Research Institute, Eastern & Southern Africa Centre of International Parasite Control<br>Agnandji, Selidji ; Hopital Albert Schweitzer, Lambaréné, Centre de Recherches Médicales de Lambaréné<br>Yaro, Seydou; Centre MURAZ<br>Alexander, Neal; London School of Hygiene and Tropical Medicine Faculty of Epidemiology and Population Health<br>Yoon, In Kyu; International Vaccine Institute, Global Dengue and Aedes-transmitted Diseases Consortium
**Primary Subject Heading**:	Infectious diseases
Secondary Subject Heading:	Infectious diseases, Epidemiology, Global health, Public health
Keywords:	dengue, Epidemiology < INFECTIOUS DISEASES, Public health < INFECTIOUS DISEASES, Tropical medicine < INFECTIOUS DISEASES

SCHOLARONE™
Manuscripts

For peer review only

Evaluating dengue burden in Africa in passive fever surveillance and sero-prevalence studies: protocol of field studies of the Dengue Vaccine Initiative

Jacqueline K. Lim[1*], Mabel Carabali[1], Jung-Seok Lee[1], Kang Sung Lee[1], Suk Namkung[1], Sl-Ki Lim[1], Valery Ridde[2], Jose Fernandes[3], Bertrand Lell[3], Sultani Hadley Matendechero[4], Meral Esen[5], Esther Andia[6], Noah Oyembo[6], Ahmed Barro[7], Emmanuel Bonnet[8], Sammy M. Njenga[6], Selidji Todagbe Agnandji[3], Seydou Yaro[9], Neal Alexander[10], In Kyu Yoon[1]

[1]International Vaccine Institute, Seoul, Korea, Republic of; [2]Montreal School of Public Health (ESPUM), Montreal, QC, Canada; [3]Centre de Recherches Médicales de Lambaréné, Hôpital Albert Schweitzer, B.P.118, Lambaréné, Gabon; [4]Communicable Disease Prevention and Control, Ministry of Health, Nairobi, Kenya; [5]Institute of Tropical Medicine, University of Tübingen, Germany; [6]Eastern and Southern Africa Centre of International Parasite Control (ESACIPAC), Kenya Medical Research Institute (KEMRI), Nairobi, Kenya; [7]AGIR, Ouagadougou, Burkina Faso, [8]Institut de Recherche pour le Développement (IRD), Paris, France; [9]Centre Muraz, Bobo-Dioulasso, Burkina Faso; [10]Epidemiology and Public Health Department, London School of Hygiene and Tropical Medicine

*Corresponding author
Jacqueline K. Lim[1*], MPH
Research Scientist
Global Dengue and Aedes-transmitted Diseases Consortium (GDAC)
International Vaccine Institute (IVI)
SNU Research Park, Gwankak-ro 1
Gwanak-gu, Seoul, Korea 151-191
T: +82-2-881-1340 (Dir) / +82-2-872-2801 (ext. 340)
Email: kalim@ivi.int

37 pages
Word count of the text: 6,619 (not counting the Abstract and table 1)
Word count of the abstract: 320
Figures and tables: 6 figures and 1 table

For peer review only - http://bmjopen.bmj.com/site/about/guidelines.xhtml

Abstract

Introduction.

Dengue is an important and well-documented public health problem in the Asia-Pacific and Latin American regions. However, in Africa, information on disease burden is limited to case reports and reports of sporadic outbreaks, thus hindering the implementation of public health actions for disease control. To gather evidence on the undocumented burden of dengue in Africa, epidemiological studies with standardized methods were launched in three locations in Africa.

Methods and Analysis.

In 2014-17, the Dengue Vaccine Initiative (DVI) initiated field studies at three sites in Ouagadougou, Burkina Faso; Lambaréné, Gabon; and Mombasa, Kenya; to obtain comparable incidence data on dengue and assess its burden through standardized hospital-based surveillance and community-based serological methods. Multidisciplinary measurements of the burden of dengue were obtained through field studies that included passive facility-based fever surveillance, cost-of-illness surveys, serological surveys, and healthcare utilization surveys. All three sites conducted case detection using standardized procedures with uniform laboratory assays to diagnose dengue. Healthcare utilization surveys were conducted to adjust population denominators in incidence calculations for differing healthcare seeking patterns. The fever surveillance data will allow calculation of age-specific incidence rates and comparison of symptomatic presentation between dengue and non-dengue patients using multivariable logistic regression. Serological surveys assessed changes in immune status of cohorts of approximately 3,000 randomly selected residents at each site at 6 month intervals. The age-stratified serosurvey data will allow calculation of seroprevalence and force of infection of dengue. Cost-of-illness evaluations were conducted among patients with acute dengue by Rapid Diagnostic Test.

Ethics and Dissemination.

The protocol for each study obtained ethical approvals from the Institutional Review Boards of the International Vaccine Institute, the London School of Hygiene and Tropical Medicine, and the Ethics Committee of host country institutions. By standardizing methods to evaluate dengue burden across several sites in Africa, these studies will generate evidence for dengue burden in Africa and data will be disseminated as publication in peer-review journals by the end of 2017.

For peer review only

Strengths of this study

- There have not been population-based studies conducted with a multi-disciplinary approach (i.e. surveillance, healthcare utilization, and sero-survey in one catchment area population). Data from the passive surveillance will be used to calculate annual incidences of dengue and data from the serosurvey will estimate force of infection and prevalence.
- The studies were conducted in three locations in Africa, based on standardized methods and laboratory algorithm. Thus, comparison by site would be possible.

Limitations of this study

For peer review only - http://bmjopen.bmj.com/site/about/guidelines.xhtml

- This is not a cohort study. The passive facility-based surveillance may lead to under-estimation of the burden of dengue fever by measuring incidence based on only those that sought care at our study facilities.
- There may be limited generalizability of our study results to other dengue-endemic parts of Africa.

Keywords: dengue; Africa; seroepidemiologic Studies; incidence

Running head: Evaluating dengue burden in Africa: DVI field studies

For peer review only

For peer review only - http://bmjopen.bmj.com/site/about/guidelines.xhtml

Background

Dengue fever, a mosquito-borne flavivirus infection caused by four related but antigenically distinct dengue viruses (DENVs, serotypes 1–4), is a major and rapidly increasing global public health problem. Recent studies have estimated an annual incidence of 50–100 million symptomatic infections globally [1]. Dengue is a high burden disease that disproportionately affects countries in the tropics and subtropics, many of which have limited health care resources [2]. Although one dengue vaccine has been recently licensed in several endemic countries, the vaccine has restricted age and epidemiological indications. Other prevention and control measures such as vector control are suboptimal as stand-alone interventions [3 4], and no drugs for treatment are currently available.

Like in Asia and the Americas, epidemics of dengue were reported from Africa in the late 19th and early 20th centuries[5 6]. Specifically for Africa, there are records of multiple dengue case reports between 1964 and 1968 with DENV 2 in Nigeria[7]. Data from several studies conducted in the 1960-70s in Nigeria supported a substantially high level of immunity in adults as well as children [8 9]. In 2011, Amarasinghe et al. conducted a comprehensive review of literature on dengue in Africa and described that dengue cases have been reported in 34 countries in Africa, with most of these countries also having *Aedes* mosquitoes [6]. However, prior studies which suggested the presence of dengue in Africa were limited by their retrospective design or sample collection (blood donors or sample collected from surveys of other diseases), and often from travellers, with a small number of reported autochthonous cases, to demonstrate the true, population-based, burden of dengue. Also, while many dengue endemic countries in Asia and Latin America have mandatory reporting of dengue cases to public health authorities, and national surveillance systems in place to monitor incidence patterns [10], most African countries lack such established reporting

For peer review only - http://bmjopen.bmj.com/site/about/guidelines.xhtml

mechanisms and only sporadic outbreaks and individual case reports have been documented. In addition, the frequently non-specific clinical presentation of dengue may be difficult to distinguish from the myriad other infectious diseases present in Africa, since dengue diagnostic assays are not widely available. Thus, the burden of dengue remains largely unknown in Africa [6 11]. Without such dengue burden data, informed decision-making about prevention and control measures, including dengue vaccine introduction, in Africa are not possible.

Limited by surveillance capacity hindering continuous reporting in the region, there had not been frequent and systematic reporting of dengue in Africa. African ancestry is known to be protective against severe dengue and the candidate genes were recently identified in Cuban patient [12 13]. Bhatt et al.'s modelling of global dengue burden suggests high burden of dengue in Africa in terms of equal numbers of, both apparent and inapparent, infections as that of Latin America[1]. There are new findings about dengue in Africa, but there is still much unknown about the magnitude of dengue problem in the continent. To improve estimates of population-based dengue disease burden in Africa and validate whether the undocumented burden of dengue is as high in Africa as in the Americas with empirical data, the Dengue Vaccine Initiative (DVI) initiated field studies at three sites in West, West-Central, and East Africa in Ouagadougou, Burkina Faso; Lambaréné, Gabon; and Mombasa, Kenya; respectively. In each of the three sites, a standardized package of study components, including passive facility-based fever surveillance, healthcare utilization surveys, cost-of-illness surveys, and serological surveys (Fig 1), was initiated between December 2014 and March 2016.

Methods

For peer review only - http://bmjopen.bmj.com/site/about/guidelines.xhtml

Site selection

Study sites were selected, in part, based on their likelihood of supporting DENV transmission. In site selection, we considered dengue outbreaks and cases reports in the literature, available seroprevalence studies, as well as country-specific dengue risk maps of the probability of DENV transmission and the level of evidence of dengue presence reporting the uncertainty of the consensus estimates of dengue in Africa [7 14]. In addition, adequate research infrastructure to implement the studies was taken into account. Finally, inclusion of different regions of Africa was also a factor in site selection. Thus, Ouagadougou, Burkina Faso; Lambaréné, Gabon; and Mombasa, Kenya; were selected, respectively, to measure the burden of dengue in selected sites from West, (West-) Central, and East Africa.

In Burkina Faso, the first reported dengue outbreak occurred in Ouagadougou in 1982 due to DENV-2 [6]. Serological prevalence of dengue was found to be 26.3% in a rural setting (Nouna village) and 36.5% in an urban setting (Ouagadougou) in 2006 [15]. More recently, an observational study conducted by Ridde et al. among febrile patients consulting at selected study facilities in 2013-14 showed 8.7 % (33/379) to be positive by dengue RDT; and 15 of 60 samples tested by RT-PCR to be dengue positive [16]. With evidence for the presence of dengue, along with a strong health and demographic surveillance system (Ouaga-HDSS) which could be used to describe the demographic characteristics of the catchment area, a field study was initiated in Ouagadougou, Burkina Faso in December 2014.

In Gabon, cases of dengue hemorrhagic fever (DHF) caused by up to three different DENV serotypes have been reported, and dengue seroprevalence has been found to be between 5 and 20% [17-19]. Results of a recently published study demonstrated seroprevalence of 12.3% among toddlers approximately 30 months of age in semi-rural Lambaréné between 2007 and 2010 [20]. However, a different study in 2005-2008 suggested minimal DENV transmission in rural areas of Gabon [21]. This latter study examined antibodies against dengue

For peer review only - http://bmjopen.bmj.com/site/about/guidelines.xhtml

in individuals from randomly selected villages representing about 10% of all Gabonese villages. Blood samples were tested by anti-DENV IgG and IgM capture ELISA and found to have only minimal IgG (0.5%) and IgM (0.5%) seroprevalence. Based on these low prevalences, authors concluded that there was no active circulation of DENV in rural Gabon. However, the low seroprevalence may have been affected by low sensitivities of the tests used leading to a high rate of false negative, and/or selection bias in the blood sample pool among the selected villagers [22]. Seroprevalence estimates may have also been impacted by the possibility of false-positive results due to IgG cross-reactivity among flaviviruses [21]. Nevertheless, given the possibility of DENV circulation in Gabon, a field study was initiated in Lambaréné in March 2015 in a community with a catchment population of about 77,000 residents, using the clinical research infrastructure of the Centre de Recherches Medicales de Lambaréné (CERMEL), benefiting from experienced research staff who conducted a large Phase 3 malaria vaccine trial [23 24].

In Kenya, more evidence is available for the presence of dengue based on local data. Dengue was the most common viral pathogen in retrospectively tested blood specimens from HIV-negative survey samples from the 2007 Kenya AIDS Indicator Survey. Antibody testing for dengue as well as chikungunya and Rift Valley fever was performed by IgG ELISA using either commercial kits or CDC assays; 12.5% were found to be dengue positive [25]. Similarly, a household survey found 13% of individuals from 701 households in Mombasa had serological evidence of either past or current DENV infection [26]. These data suggest that there is more dengue in Kenya than indicated by public health reporting, possibly due to misdiagnosis [25 26]. A field study was initiated in Mombasa, Kenya in March 2016.

Study participants

For peer review only - http://bmjopen.bmj.com/site/about/guidelines.xhtml

For the passive facility-based fever surveillance, individuals who met the following criteria were eligible for study enrollment:

1. Age 1- 55 years old;
2. Resident of the catchment area covered by healthcare facilities participating in the study, without plans to move out of the catchment area within 12 months;
3. Signed informed consent, and assent for those aged between 7 and 18 years; and
4. Patients presenting with current fever (axillary temperature ≥ 37.5º C) or history of fever for ≤ 7 days duration without localizing signs (fever caused by a localized infection as well as fever with a known and confirmed etiology other than dengue, such as malaria confirmed by malaria RDT- listed in the patient identification SOP).

For the serological survey, criteria 1-3 were applied. For the healthcare utilization survey, household interviews were conducted among the heads or representatives of the household invited from each family participating in the serosurvey.

Study area and population

Burkina Faso, located in West Africa, has a population of 14,017,462 with 22.7% living in urban areas. The country is mainly rural with about 29% of the population reported to be living in urban areas in 2014. However, Burkina Faso is urbanizing rapidly and is positioned as the country with the fourth fastest urbanization in the last 25 years [27 28]. The capital, Ouagadougou, has a population of 2,741,128. The majority of the population live in urban settings. About 45% of the population are under 15 years of age [29]. The city is divided into 12 districts and 52 sectors. Ouagadougou is the country's largest city and the cultural and economic center. The city is part of the Soudano-Sahelian area, with a rainfall of about 800

For peer review only - http://bmjopen.bmj.com/site/about/guidelines.xhtml

For peer review only

mm per year. The rainy season is from May to October, with a mean temperature of 28 °C (82 °F). The cold season runs from December to January, with a minimum average temperature of 16 °C (61 °F). During the hot season, which runs from March to May, the temperature can reach as high as 43 °C (109 °F).

The Health and Demographic Surveillance System is in place in Ouagadougou. Ouaga HDSS monitors a population of 81,717 residents; according to this surveillance system, the city population is very stable with a rate of migration of 4.1% and more than 80% of the inhabitants with ownership of their houses [20]. A map of the city and distribution of the population is shown in Figure 2.

Gabon, located on the west coast of Central Africa, has an area of nearly 270,000 square kilometres (100,000 sq. mi) with a population estimated at 1.5 million. Its capital and largest city is Libreville. In 2014, it is reported that 87% of the Gabonese population lived in urban areas [28]. The sixth largest city, Lambaréné, the capital of Moyen-Ogooué, is located 75 kilometers south of the equator, with a population of 25,257 in 2009. The majority of Lambaréné residents live in semi-rural areas. About 42% of the Gabonese population is under 15 years of age [29]. Similarly, Lambaréné's population is relatively young with about 50% under 20 years of age.

The health services of Gabon are mostly public, but there are some private institutions as well. With one of the best medical infrastructure in the region, almost 90% of the population have access to health care services. Albert Schweitzer Hospital (ASH) is a private institution which served as a study site for the passive fever surveillance study [30 31]. The study area in Lambaréné is shown in Figure 3.

Kenya, located in East Africa, lies on the equator, covering 581,309 km^2 (224,445 sq. mi), with a population of approximately 45 million people in 2014 [2]. Kenya

For peer review only - http://bmjopen.bmj.com/site/about/guidelines.xhtml

generally has a warm and humid tropical climate, but is diverse ranging from the cooler climate around the capital city, Nairobi, to a hot and dry climate inland, as well as a desert-like climate in the north-eastern regions along the border with Somalia and Ethiopia [32]. The capital, Nairobi, is a regional commercial hub. The main industries include agriculture, exporting tea and coffee, as well as the service industry.

Kenya is divided into 47 semi-autonomous counties. Mombasa is the country's second largest city after Nairobi and is located on the east coast of the country [2]. Administratively, Mombasa is the capital of Mombasa County, what was formerly called Coast Province. This overall Coast region covers over 80,000 km^2 in the south-eastern part of Kenya, constituting about 15% of the country's land area, with a population of 3,325,307 residents.

The main driver of economy of Mombasa is tourism and trading industry. Mombasa itself has a population of about 1.3 million with almost 50% of the population under 15 years of age [29]. Increasingly, the population of the province lives in urban areas; at present about 45% live in Mombasa and other urban centers. The long rains begins around April and the short rains begins in October [32]. Mean annual temperature ranges from 24°C to 27°C, but maximum temperature averages over 30°C during the hottest months, January to April.

Figure 4 shows the area of Mvita subcounty of Mombasa, which was the catchment area for the study in Kenya, with a catchment population of 74,735 residents. The map indicates the three facilities involved in the study.

Sample size

Given the paucity of available age-specific dengue incidence data in the study countries or nearby countries, it was difficult to obtain population-based incidence to make assumptions when calculating sample sizes. The required catchment population for the

For peer review only - http://bmjopen.bmj.com/site/about/guidelines.xhtml

passive facility-based fever surveillance was roughly estimated based on the limited data available in the literature. Annual incidence estimates were calculated based on available prevalence estimates with the assumption that the outcome of interest has zero prevalence at age zero, and that force of infection is constant. It was assumed that prevalence estimates found for one particular age group would be adjusted as the annual incidence and used across all ages.

Wichmann et al. calculated an expansion factor for children by comparing data from three cohort studies to national surveillance data in Southeast Asia [33]. For children in Thailand, the age-specific expansion factors calculated were 11.85 for <5 years, 8.76 for 5-9 years, and 7.81 for 10-14 years [33]. The results show that, even for Asia where better reporting and surveillance systems are available, there is a considerable degree of underreporting. For Africa, there may be more dengue cases under-ascertained (not seek care) and under-reported (not reported even if a patient with dengue seek care as dengue is not one of the routinely notifiable diseases in Africa), but such information on extent of underestimation of dengue was not available [34 35]. Also, the incidence estimates used in our sample size calculations were not from population-based studies. While it would have been ideal to adjust the incidence further for likely underestimation, the annual incidence used in sample size calculations could not be adjusted for possible under-reporting due to the lack of data. The sample sizes were calculated with 95% confidence levels and a margin of error at a fixed significance level within 25% of the true proportion of incidence. This gives relative precision of 75%, considering the gap in evidence for dengue incidence in the study areas. The final sample sizes were calculated by assuming 20% non-response rate or loss to follow-up. The required catchment population size for the fever surveillance study in Burkina Faso was estimated to be 100,000, Gabon to be 77,000, and Kenya to be 70,000. In these catchment populations, the number of enrolled subjects depends on the number of eligible

For peer review only - http://bmjopen.bmj.com/site/about/guidelines.xhtml

patients who seek care at the study facilities. How many eligible febrile episodes would actually present at our study facilities was difficult to predict; but after assessment of the volume of febrile patients at the facilities, a realistic upper limit for enrollment for a study period of approximately 1.5 years was set at 3,000 subjects to offer enrollment to all consenting eligible patients.

For the serological survey, the sample size was calculated similarly using the prevalence proportion based on published literature. Seroprevalence of 0.304 for Burkina Faso [15], 0.123 for Gabon [21], and 0.144 for Kenya [36] were used. With the same confidence levels and allowed margin of error, and assuming 10-30% (variable by site) non-response rate, the sample size was calculated to be 3,000 participants at each site. Again, with the scarcity of data from the selected countries, there were no other prevalence estimates reported or estimates from different age groups. As prevalence is expected to increase with age, and higher prevalence would give a smaller sample size, our calculations are likely to be conservative.

Study components

Fever surveillance – design and methods

To determine burden due to symptomatic dengue in each of the three sites in Burkina Faso, Gabon, and Kenya, passive facility-based fever surveillance was implemented in a well-defined catchment area population. In Burkina Faso, the surveillance study was initiated in December 2014 in five selected primary health care centres, locally called “Centre de Santé et de Promotion Sociale” (CSPS), in the municipality of Ouagadougou, with a catchment population of 105,000 residents. This project was implemented in collaboration with Centre Muraz in Bobo-Dioulasso, EQUITE sante program (a collaborative program

For peer review only - http://bmjopen.bmj.com/site/about/guidelines.xhtml

between University of Montreal and Action-Gouvernance-Integration-Reinforcement, AGIR, based in Ouagadougou, funded by Canadian Institute of Health Research), and DVI. In Gabon, the surveillance study was initiated in the Albert Schweitzer Hospital serving a catchment population of 130,000 residents in the Moyen-Ogooué and surroundings within Lambaréné, in collaboration with CERMEL and Institute of Tropical Medicine in Tubingen, Germany. In Kenya, the surveillance study was implemented at Ganjoni dispensary, Tudor sub-county Hospital, and Coast Provincial General Hospital, serving a catchment population of 70,000 residents in Mombasa, in collaboration with Kenya Medical Research Institute (KEMRI) and Ministry of Health of Kenya.

As described in Figure 5, both outpatients and inpatients at the designated study facilities, who meet inclusion criteria as mentioned earlier are tested for dengue, first with SD Dengue Duo® RDT. Dengue confirmation is done by detection of dengue virus in serum samples using PCR, as well as anti-dengue IgM and IgG antibodies in acute and convalescent serum by ELISA (SD Dengue IgM & IgG capture ELISA® tests, Standard Diagnostics, Yongin-Si, Korea) [10 37]. Every consecutive patient meeting inclusion criteria is eligible for enrolment during the study period. Infants < 1 year old were not included due to operational limitations, such as difficulty of infantile bleeding.

In Ouagadougou, Burkina Faso, the fever surveillance initiated in December 2014 continued until February 2017 (approximately 2 years). In Lambaréné, Gabon, the fever surveillance initiated in April 2015 continued until January 2017 (approximately 1.5 years). In Mombasa, Kenya, the fever surveillance initiated in March 2016 will continue until June 2017.

Among subjects enrolled in the fever surveillance, those who are positive by dengue

For peer review only - http://bmjopen.bmj.com/site/about/guidelines.xhtml

rapid diagnostic test are offered further enrolment in the cost-of-illness survey, consisting of interviews on the day of acute illness visit, day 10-14 from the first visit, and day 28, if illness continues. The cost-of-illness survey questionnaire was designed to estimate the direct medical, direct non-medical, and indirect costs associated with dengue-positive patients identified at study facilities. This survey also estimates the cost of treating dengue at the facility level. Data are gathered by linking patients' medical records concerning outpatient visits, inpatient visits, and service consumption (e.g., diagnostic tests, medication, and other services provided to patients). The cost-of-illness portion of the study will be described separately.

Fever surveillance – laboratory testing

As shown in Figure. 6, in all three sites, acute samples are tested using a commercial RDT for dengue NS1 and IgM/IgG (Dengue Duo®, Standard Diagnostics, Yongin-Si, Korea). Dengue Duo RDT is used on the day of acute illness visit at the site of patient presentation (day 1). The acute and convalescent samples are subsequently tested at a local laboratory using dengue IgM/IgG ELISA (SD Dengue IgM & IgG Capture ELISA®, Standard Diagnostics, Yongin-Si, Korea). The serum is separated and stored in 4 aliquots of about 500 μL for various lab tests, as indicated in consent documents.

After ELISA testing, samples will be shipped to IVI in Korea. Samples with positive results by RDT or ELISA undergo further testing by RT-PCR at the Clinical Immunology Laboratory of IVI. Four DENV serotype-specific real-time RT-PCR assays are used for laboratory confirmation of dengue and serotyping [38]. The DENV 1-4 RT-PCR assays are carried out in 25μL reaction mixtures containing 5μL template RNA, TagMan® Fast Virus 1-

For peer review only - http://bmjopen.bmj.com/site/about/guidelines.xhtml

step mastermix (Applied Biosystems®), 0.9 μM of each primer, and 0.2 μM probe [38].

Amplification and detection are performed in a StepOne Plus real-time PCR system and the baseline and threshold are determined using the auto-baseline and threshold feature in StepOne Software v2.2.2 (Applied Biosystems®). Thermocycling parameters are as follows: reverse transcription at 50 °C for 5 min, inactivation at 95 °C for 20 s, followed by 45 cycles of fluorescence detection at 95 °C for 3 s, and annealing at 60 °C for 30 s [38]. A specimen is considered positive if target amplification was recorded within 40 cycles.

Serological survey – design and methods

While the facility-based fever surveillance studies provide estimates of the burden of medically-attended dengue disease, evaluation of all DENV infections in a population – including subclinical and mildly symptomatic infections, which impact immune status – is needed to capture the overall impact of dengue. As part of the study package, population-based serological surveys were conducted in the same catchment population used for the fever surveillance. At each of the three sites in Africa, the serosurvey was conducted on a cohort of approximately 3,000 randomly selected residents of urban and semi-urban parts of Ouagadougou, Lambaréné, and Mombasa. Without individual-level census information on all residents of Lambaréné and Mombasa, with help of community/village health workers, randomization was done based on neighbourhoods (or defined areas for which the health workers/volunteers are responsible) as cluster units. As the community/village health workers are familiar with the villages and their residents, they are good entry points into the communities. With these health workers, the field team screened houses in the selected villages by knocking on doors of every 5~7 houses, depending on the household density per neighbourhood. Also, demographic information collected in previous research projects

For peer review only - http://bmjopen.bmj.com/site/about/guidelines.xhtml

conducted in the same area was used as a guide, if available. In the case of the site in Ouagadougou, HDSS data were available and the EQUITE SANTE, a CIHR funded research program of University of Montreal had set up a geographic information system (GIS) database system of houses in the study area. Using these data, households of potential enrollees of the serosurvey were pre-selected randomly and household visits were made in Ouagadougou. In the three sites, about 45% of the serosurvey samples were targeted to be collected from children 1 - 14 years-of-age, and 55% were targeted to be collected from adults between 15 and 55 years of age to reflect the age distribution of the general population of the area. Household-based enrollment was offered to the head of the household until the specific cap for the age-group was reached in Lambaréné and Mombasa.

Randomly-selected subjects 1-55 years of age underwent phlebotomy (5ml for children and 7ml for adults) twice — during pre-transmission (before the rainy season) and post-transmission (after the rainy season) at 6 month intervals. The sera were evaluated using IgG indirect ELISA at baseline and after 6 months. The presence of dengue IgG antibodies at 6 month intervals will be used to estimate the level of occurrence of inapparent DENV infection and to calculate the rate of infection in the catchment population. Flow cytometry-based DENV neutralization assays will be applied to a subset of samples to assess for presence of dengue neutralizing antibodies and seroconversion over the 6 month interval. In addition to overall seroconversion, age-specific seroconversion estimates in the catchment population, as well as the proportion of inapparent infections, are determined.

Serological survey – laboratory testing

From the samples collected in the serosurvey, about 200 μL of serum are used and tested at a local laboratory using dengue IgG ELISA (Panbio Dengue IgG Indirect ELISA®,

For peer review only - http://bmjopen.bmj.com/site/about/guidelines.xhtml

Alere North America, LLC, Florida, United States). After ELISA testing for dengue IgG at the local labs, samples will be shipped to IVI. Given potential serological cross-reactivity among flaviviruses [39], flow cytometry-based neutralization assays will be performed against selected flaviviruses to include yellow fever virus, West Nile virus, Zika virus, and Japanese Encephalitis virus at the Clinical Immunology Lab of the International Vaccine Institute (IVI), Seoul, Korea [40 41]. About 50 samples per bleed for 4 bleeds in Burkina Faso and 2 bleeds in Gabon and Kenya will be tested.

About 1,000 μL of serum is kept aside for this procedure. The flow cytometry-based neutralization assays are performed in duplicate in 96-well cell culture plates with flat-bottom wells, each containing DC-SIGN-expressing U937 cells [40]. The amount of virus used in the assay infects between 7 and 15% of the cells. Human immune sera are serially diluted and the virus is pre-incubated with the sera for 1 h at 37°C [40]. The cells are washed, and the virus and serum mixture is added to the cells for 1 h at 37°C, and the cells are further incubated for 24 to 48 h at 37°C in 5% CO_2. The cells are fixed, permeabilized, and stained with fluoresce-conjugated monoclonal antibody 4G2, which recognizes the flavivirus E protein [42]. FACScan flow cytometer (Becton Dickinson, San Diego, CA) is used to analyze the cells [40]. The serum dilution that neutralized 50% of the viruses is calculated by nonlinear, dose-response regression analysis with Prism 4.0 software (GraphPad Software, Inc., San Diego, CA).

In addition, a Luminex-based multiplex immunoassay will be performed on a randomly selected sub-sample to assess for IgG to different flaviviruses [43]. About 200 samples per bleed for 4 bleeds in Burkina Faso and 2 bleeds in Gabon will be tested. Detection of IgG against ZIKV and each the four DENV serotypes was performed on patient serum samples using an in-house microsphere-based multiplex immuno-assay (arbo-MIA) at the Clinical Immunology Lab of IVI [44 45]. The arbo-MIA is based on a mixture of microspheres covalently coupled with either DENV-1, -2, -3, -4 or ZIKV recombinant

For peer review only - http://bmjopen.bmj.com/site/about/guidelines.xhtml

antigens (E protein domain III) produced in Drosophila S2 expression system. Briefly, microsphere mixtures were sequentially incubated in the dark under constant shaking with a 1:400 dilution of patient serum samples, with 2 µg/mL anti-human IgG biotin-conjugated antibody (Jackson Immunoresearch, West Grove, PA) and with 2 µg/mL streptavidin-R-phycoerythrin conjugate (Life technologies). After the final incubation, the median fluorescence intensity (MFI) of each microsphere set was quantified using a BioPlex 200 instrument (Bio-Rad Laboratories, Hercules, CA). Samples were considered seropositive if the ratio of MFI values obtained for the viral antigen to the control antigen was superior to the defined cut-off. The cut-off of the MIA was determined for each viral antigen by ROC curve analysis using well characterized sera.

In Lambaréné, the enrolment bleed, took place in November -December 2015 with 2nd blood collection in May 2016. In Ouagadougou, the enrolment bleed took place in May-June 2015 with follow-up blood collections in December 2015, June 2016, and January 2017. In Mombasa, enrolment bleed took place in May 2016 with the 2nd blood collection in November 2016 – February 2017.

Healthcare Utilization Survey

As the passive fever surveillance is conducted at the study facilities, potential dengue patients could be missed if they seek care elsewhere. To identify the proportion of fever and dengue cases potentially missed by the passive surveillance system due to patients living in the study area but seeking care outside of study facilities, a population-based healthcare utilization survey was conducted in 400 randomly selected households from the study catchment area to characterize their healthcare utilization patterns of the households when they have (self-reported) febrile episodes among the family members. In addition to assessing

For peer review only - http://bmjopen.bmj.com/site/about/guidelines.xhtml

health-seeking behaviours of the residents, preferences in terms of health-seeking behavior and respective reasons for their preferences were investigated. The questionnaire was administered to 400 heads of households. Among 3,000 residents who participated in the serosurvey, there were about 600 households. From these households, 400 heads of households were randomly selected and offered enrolment in the health utilization survey. Heads of households or a senior representative within the household were asked questions on health seeking patterns of their family members. The surveys provide data to determine the proportion of these cases missed by our passive fever surveillance system. Also, the surveys document health seeking patterns, who would seek care at which facilities, who would make health-seeking related decisions, and the reasons for their preferences.

Study questionnaires

For the fever surveillance study, questionnaires are administered at the acute illness visit and the convalescent visit. The convalescent visit may take place at the health care facility (10-14 days later) or at the patient's home (15-21 days after the acute visit), according to patient preference and availability. The questionnaires are completed by medical staff of the study facilities, including demographic and clinical information (e.g., signs, symptoms, past medical history, treatments prescribed, and diagnoses). The same staff also complete the follow-up questionnaire at the convalescent visit within 21 days from the acute visit. Study nurses complete surveillance enrolment log. Lab technicians complete the lab section (mostly dengue-related diagnostics) and the forms are compiled by the study coordinator on site.

For the serosurvey component, questionnaires are administered at the household by trained field team staff at each serosurvey visit. Study nurses complete the questionnaire after a brief physical and medical examination. There are (a) follow-up visit(s) in about 6 months and the same staff make the household visits to complete the follow-up questionnaire. Enrolment log

For peer review only - http://bmjopen.bmj.com/site/about/guidelines.xhtml

is maintained by our study coordinator on site.

Variables of the surveillance questionnaires

The variables collected are listed in table 1.

Table 1. List of variables collected in the passive fever surveillance data collection form

Topic	Description	Items
Basic information	Demographic and basic information about the patient and the treatment received	Type of treatment where patient is enrolled (IPD vs. OPD)<br>Date of fever onset, duration of fever<br>Current temperature<br>Tourniquet test results<br>Patient's address (district and village-level)<br>Date of visit, date of birth, age, and sex<br>Weight and height
General health condition	Current condition of the patient (self-report) and underlying diseases of the patient	How well the patient could handle daily activities<br>Pre-existing conditions
Signs and symptoms during this illness	A set of sign and symptoms that may be related to fever and dengue (DF and DHF)	Rash, fatigue, headache, retro-orbital pain, neck/ear pain, sore throat, breathing difficulty, cough, expectoration, gastrointestinal signs (Nausea/vomiting, diarrhea, abdominal pain, etc.), hemorrhagic signs (nose/gum bleeding, ecchymosis, petechiae, etc.), signs of shock (cyanosis, capillary refill), arthralgia, myalgia, loss of appetite, jaundice, etc.
Medical History:	Previous dengue-related or other flavivirus infection as well as vaccination history (self-report)	Previous dengue infection and related hospitalization<br>Previous infection to other commonly circulating arboviral infection in the area<br>Yellow fever vaccination history
Laboratory findings	Records from the routine laboratory tests widely used in clinical fever/dengue patient management, as part of the hospital care procedure	Platelet count, hematocrit, haemoglobin, leukocytes, neutrophils, protein level, AST, ALT, urine test results, etc.

For peer review only - http://bmjopen.bmj.com/site/about/guidelines.xhtml

Clinical Diagnosis	Clinician's diagnosis with or without referring to the RDT	Diagnosis given by the physician based on clinical presentation after physical examination of the patient.
Dengue testing results	Results from the dengue tests, mainly RDTs for dengue as well as other commonly circulating arbovirus in the area	Dates of blood draw<br>Test results of the RDT<br>IgM/IgG capture ELISA results<br>PCR results (if available)
Treatment	Medicine(s) prescribed and the starting and end dates	Antibiotics, paracetamol, ibuprofen, aspirin, and others that may be site-specifically prescribed
Outcome	Outcome of this particular visit	Hospitalized, returned home, or referral
Hospitalization	Information collected only among hospitalized patients in the surveillance to record other severe signs and progression of illness	Admission and discharge diagnoses<br>Presence of haemorrhagic signs or shock syndrome
Hospital Charges	Expenses and hospital charges incurred by patient on the visit 1	Amount of the out of pocket payment by the patient or the family/or guardian<br>Breakdown of the hospital charges (laboratory, medication, admission-related charges)
Final outcome	Outcome of the patient's illness at the 2nd visit	Final diagnosis given for the patient outcome of illness<br>Completion of study participation (early termination and the reason, etc.)

Planned statistical analysis

From the fever surveillance data, incidence of symptomatic dengue among patients that seek health care at the study facilities will be calculated. Age-specific incidence rates in all the children and adults will be determined by referring to the size and distribution of the general population of the study area at the time of surveillance as the denominator in calculation of the incidence of symptomatic dengue cases. Each person residing in the study area is assumed to contribute 12 months of person time to the denominator. Although the study areas all report a low migration rate, the in-migration is assumed to balance the out-

For peer review only - http://bmjopen.bmj.com/site/about/guidelines.xhtml

migration of the population during the study period. Age-specific incidence of symptomatic dengue will be calculated by using age-specific denominators and the number of symptomatic dengue cases in eligible individuals as the numerator.

Using the data collected in the Healthcare Utilization Survey, the proportion of febrile cases missed by the passive surveillance system will be determined. Then using the proportion, the numerator will be further adjusted in recognition of those missed fever cases from the study area, which could have been dengue. Also, comparison will be made between those that agreed to participate and those that declined participation among the eligible potential enrollees. The enrollment log, which recorded basic information obtained during the screening process of potential enrollees, will be reviewed. In addition to checking that our sample of febrile cases is representative of febrile patients of the general population in the catchment area, refusal rates will be determined based on information in the log. Then, the refusal rates will be used to adjust the numerator.

SPSS software will be used for analysis of the fever surveillance data. Multivariable logistic regression will be used to compare confirmed dengue patients versus non-dengue febrile patients in terms of symptomatic presentation, based on signs and symptoms collected from all patients with laboratory-confirmed dengue by serology and RT-PCR, adjusting for possible confounders, such as age, days since onset of fever, primary vs. secondary infection, inpatient vs. outpatient, etc. Differences in symptomatic complex of DF (and DHF, if data allows) by age and serotype will be also determined using multivariable logistic regression.

As outpatient disease accounts for the greater part of dengue disease burden, clinical profile of individuals with DENV infection will be characterized by the type of treatment (hospitalized and outpatients), as well as by severity of the disease (severe vs. non-severe by the 2009 WHO criteria) [46]. Classification is determined after the course of illness is completed (typically during the convalescent visit). Symptomatic dengue is classified as

For peer review only - http://bmjopen.bmj.com/site/about/guidelines.xhtml

outpatient or hospitalized. Progression of dengue is recorded in the CRF as DF, DHF I, DHF II, DHF III or DHF IV and clinical patterns will be compared by the severity grade [46 47]. These will be compared to results obtained from other DVI studies in Latin America (Colombia) and Asia (Thailand, Vietnam, and Cambodia). Overall, comparisons will be made across Burkina Faso, Gabon, and Kenya.

With the age-stratified sera that reflect the age distribution of the general population of the country, the serological survey sampling strategy ensures sufficient subjects to obtain precise age-specific estimates of sero-positivity and sero-conversion of the catchment area population. The sero-conversion rate and change in the immune status will be determined by age group during the study period. The age-stratified serosurvey data will also allow calculation of the force of infection of dengue in the study population. After enrolment, there were subjects who drop out in the follow-up bleeds about 6 months later. Basic demographic information will be compared between those that completed participation and those with incomplete participation to check whether our sample represents the catchment area population. Comparisons will be made among Burkina Faso, Gabon and Kenya.

Ethical considerations

To minimize inconvenience of the study to patients, clinicians and nurses were sensitized and trained regarding the study requirements and procedures in order for data collection to be integrated into routine patient care. The clinicians and nurses selected for the study receive coordinated support from study field staff throughout the study process. Written informed consent, and assent for participants 7 (13 for Kenya) -17 years of age, were obtained from patients by study staff. Study staff go through consent and assent documents for short summary of the disease, detailed description of study procedure, and information on

For peer review only - http://bmjopen.bmj.com/site/about/guidelines.xhtml

reimbursement. Patient data are documented in the study designated office and only the study staff have access to the data that had been de-identified without any personal identifiable information. Data are exclusively handled in the study office and stored safely in a protected database in the study office as well as the DVI main server.

The protocol for each study obtained ethical approvals from the Institutional Review Boards (IRBs) of the International Vaccine Institute, the London School of Hygiene and Tropical Medicine, and the Ethics Committee of host country institutions, including KEMRI Scientific and Ethical Review Unit in Kenya, Gabon National Ethics Committee and Institutional Ethics Committee, Scientific Review Board of CERMEL in Gabon, and the IRB of Centre Hospitalier de l'Université de Montréal (CRCHUM) at University of Montreal, and the National Health Ethical Committee of Burkina Faso.

Discussion

Dengue cases have been detected since 1960's in Africa and there has been continued presence of the *Aedes* vectors in the continent [5 7]. However, very few dengue studies have been conducted in Africa. Also, little evidence is from population-based studies [6]. Compared to the volume of evidence from SE Asia and the Americas, there is critical data scarcity on dengue in Africa. Suspicion of substantial dengue burden in Africa is based on limited reports of outbreaks and a handful of sero-prevalence studies testing different viruses among samples that likely do not represent the general population. In the three countries selected for our field studies, somewhat more data were available but were still very limited. In Burkina Faso, a recent observational study conducted in 2013 reported that 8.7% of the febrile patients showed positive results on dengue RDT [16]. In Gabon, one study suggested minimal DENV circulation in rural areas [21], while a recent study reported 12.3% seroprevalence, by IgG antibodies against dengue, among toddlers 30 months of age in semi-

For peer review only - http://bmjopen.bmj.com/site/about/guidelines.xhtml

rural parts of Lambaréné [20]. In Kenya, about 13% of the individuals in Mombasa have been reported evidence of past or current DENV infection by RT-PCR and IgM anti-dengue ELISA after the 2013 outbreak [26]. Despite the limited scope and generalizability of these studies, they suggest that there may be more dengue than previously appreciated due to underestimation and misdiagnosis [25 26].

These studies all indicate presence of dengue and some level of underlying sero-prevalence in the countries of our field studies. However, often these studies are limited by their retrospective design or sample collection (blood donors or sample collected from surveys of other diseases) to demonstrate the true, population-based, burden of dengue. We proposed to address this gap by population-based dengue surveillance and sero-prevalence studies in West, (West-) Central, and East Africa.

The present studies at three sites in Africa will provide important information on undocumented DENV circulation in Africa. Such data will help to strengthen the evidence base for dengue burden in Africa. Better defined disease burden data based on our studies could be used to assess the relative need for dengue prevention and control measures, such as whether a dengue vaccine would be a cost-effective public health intervention for countries in Africa. Clinical findings from our studies could also be used as a guide for dengue case detection and case management.

The studies have some important limitations. We recognize variability of the dengue epidemiology over time and by the region. Due to resource constraints, our studies are limited in terms of time frames and geographically focused for measuring dengue illnesses and infections. Therefore, it may limit generalizability of our data collected from our studies.

One potential source of bias in estimating the incidence of symptomatic dengue is under-ascertainment due to the community residents with relevant symptoms seeking care from other healthcare providers and facilities than the facility under surveillance. As the

For peer review only - http://bmjopen.bmj.com/site/about/guidelines.xhtml

study design remains passive surveillance, cases are ascertained only at our study facilities. By estimating the proportion of febrile patients seeking care elsewhere, as well as refusal rates among the potential enrollees that were screened for eligibility criteria, the degree of fever patients missed by the study facility will be determined. Inverse probability weighting will be used to account for these potential subjects missed by the surveillance as adjustments in incidence calculation. Also, depending on the transmission volume of dengue or other co-circulating diseases with onset of fever there may be patients that are diagnosed with other diseases and ruled out of dengue. Furthermore, with respect to dengue diagnostics for our serological surveys, there are other circulating flaviviruses in Africa leading to challenges in identifying human antibodies that may be due to past dengue infections. While our testing plan covers some of the common flaviviruses, there are others in circulation in Africa, such as Banzi and Usutu viruses [48-50]. Due to resource limitations, our plans for laboratory testing include yellow fever virus, West Nile virus, Zika virus, and Japanese Encephalitis virus as well as DENV 1-4. As our serological surveys were geographically focal and limited to a number of viruses, we recognize diagnostic limitations where we may possibly miss out on true dengue-positive individuals. These factors may increase the likelihood of under-reporting or over-reporting.

In addition, the sero-survey and healthcare utilization survey are conducted on a randomized sub-sample of the catchment area population and there may be limited generalizability of the data collected from these surveys. With unknown differences among those that agree to participate and those that do not agree, the data may not be representative of the general population of the study countries.

Conclusions

For peer review only - http://bmjopen.bmj.com/site/about/guidelines.xhtml

Data collection continues and study closure is planned in all three sites in April – June 2017. Although not described in this paper, preliminary results indicate a substantial level of dengue incidence and prevalence in each site. The data collected from our studies will provide a more accurate assessment of the unknown dengue disease burden in Burkina Faso, Gabon, and Kenya. These data can fill a gap in undocumented burden of dengue in the region and, collectively, may be used to infer dengue burden in other areas of Western, Central, and Eastern Africa. Countries in Africa may not consider introduction of a dengue vaccine as the foremost priority in the near future due to many other competing public health problems and limited resources as a major challenge. For cost-effective implementation of public health interventions, accurate data on dengue burden from epidemiological studies would be needed for policy makers to make evidence-based decisions on control and prevention of dengue. Our studies will provide some much needed information based on population-based research to assess dengue burden in Africa.

For peer review only

For peer review only - http://bmjopen.bmj.com/site/about/guidelines.xhtml

List of abbreviations

GDAC - Global Dengue and *Aedes*-transmitted Diseases Consortium
IVI - International Vaccine Institute
DENV- dengue viruses
DVI - Dengue Vaccine Initiative
Ouaga-HDSS - health and demographic surveillance system
DHF - dengue hemorrhagic fever
CERMEL - Centre de Recherches Médicales de Lambaréné
ASH – Albert Schweitzer Hospital
CSPS - Centre de Santé et de Promotion Sociale
KEMRI - Kenya Medical Research Institute
SD – Standard Diagnostics
GIS = geographic information system
MFI - median fluorescence intensity
CRCHUM - Centre Hospitalier de l'Université de Montréal

For peer review only

For peer review only - http://bmjopen.bmj.com/site/about/guidelines.xhtml

Declarations

- Ethics approval and consent to participate

The protocol for each study obtained ethical approvals from the Institutional Review Boards (IRBs) of the International Vaccine Institute, the London School of Hygiene and Tropical Medicine, and the Ethics Committee of host country institutions, including KEMRI Scientific and Ethical Review Unit in Kenya, Gabon National Ethics Committee and Institutional Ethics Committee, Scientific Review Board of CERMEL in Gabon, and the IRB of Centre Hospitalier de l'Université de Montréal (CRCHUM) at University of Montreal, and the National Health Ethical Committee of Burkina Faso.

- Consent for publication

Not applicable

- Availability of data and material

Data sharing is not applicable to this article as no datasets were analyzed during the current study.

This manuscript does not include data from the studies described here in. This is a protocol paper. The datasets that are being generated for analysis as described in the current study are not yet publicly available as the studies are currently ongoing at the time of submission. They will be available from the corresponding author on reasonable request.

- Competing interests

The authors declare that they have no competing interests.

- Funding

The current study was supported by funds from the Bill and Melinda Gates Foundation (OPP 1053432). The funding body had no role in the design of the study and collection, analysis, and interpretation of data and in writing the manuscript.

For peer review only - http://bmjopen.bmj.com/site/about/guidelines.xhtml

- Authors' contributions

JKL designed the study, is overseeing data collection, and was a major contributor in writing the manuscript. MC co-designed the study, oversaw some parts of data collection, and supported in writing of the manuscript. JSL was a contributor in designing of the study and oversight of parts of data collection. KSL was a contributor in oversight of data collection. SN supported in data collection. SKL supported in data collection. VR supported in designing of the study and was a major contributor in finalization of the manuscript. JF was a contributor in data collection. BL was a contributor in designing of the study and data collection. SHM was a contributor in designing of the study and site establishment. ME was a contributor in designing of the study. EA supported in data collection. NO supported in data collection. AB supported in data collection. EB supported in data generation. SMN was a contributor in designing of the study and site establishment. STA was a contributor in designing of the study and site establishment. SY was a contributor in designing of the study and site establishment. NA was a major contributor in providing oversight of the data collection and finalization of the manuscript. IKY was a major contributor in designing of the study and finalization of the manuscript.
All authors read and approved the final manuscript.

- Acknowledgements

We thank the doctors and laboratory staff of CSPS of Ouagadougou, ASH, Ganjoni dispensary, Tudor sub-county Hospital, and Coast Provincial General Hospital, as well as at Centre Muraz, CERMEL, and KEMRI. We thank collaborators at AGIR, IRD France, and University of Montreal, and University of Tubingen. Lastly, we would like to thank the DVI team and statisticians and administrative staff at the International Vaccine Institute for their helpful comments during the preparation of this manuscript and support during the studies.

For peer review only

References

1. Bhatt S, Gething PW, Brady OJ, et al. The global distribution and burden of dengue. Nature 2013;**496**(7446):504-7.
2. Murray NE, Quam MB, Wilder-Smith A. Epidemiology of dengue: past, present and future prospects. Clin Epidemiol 2013;**5**:299-309.
3. Chang M, Christophel E, Gopinath D, et al. Challenges and future perspective for dengue vector control in the Western Pacific Region, Regional Analysis. Western Pacific Surveillance and Response Journal 2011;**2**(2):9-16.
4. Eisen L, Beaty B, Morrison A, et al. ProactiveVector control strategies and improved monitoring and evaluation practices for dengue prevention. J Med Entomol 2009;**46**(6):1245-55.
5. Gubler D, Clark G. Dengue/dengue hemorrhagic fever: the emergence of a global health problem. Emerg Infect Dis 1995;**1**(2):55-57. .
6. Amarasinghe A, Kuritsky J, Letson G, et al. Dengue virus infection in Africa. Emerg Infect Dis 2011;**17**(8):1349-54. .
7. Messina JP BO, Scott TW, Zou C, Pigott DM, Duda KA, Bhatt S, Katzelnick L, Howes RE, Battle KE, Simmons CP, Hay SI. Global spread of dengue virus types: mapping the 70 year history. Trends Microbiol 2014;**22**(3):138-46.
8. Fagbami A, Monath T, Fabiyi A. Dengue virus infections in Nigeria: a survey for antibodies in monkeys and humans. Trans R Soc Trop Med Hyg 1977;**71**(1):60-5.
9. Carey D, Causey O, Reddy S, et al. Dengue viruses from febrile patients in Nigeria, 1964-68. Lancet 1971;**1**(7690):105-6.
10. Beatty M, Stone A, Fitzsimons D, et al. Best practices in dengue surveillance: a report from the Asia-Pacific and Americas Dengue Prevention Boards. PLoS Negl Trop Dis 2010;**4**(11):e890.
11. Were F. The dengue situation in Africa. Paediatrics and Internatinoal Child Health 2012;**32**(S1):18-21.
12. Sierra B, Triska P, Soares P, et al. OSBPL10, RXRA and lipid metabolism confer African-ancestry protection against dengue haemorrhagic fever in admixed Cubans. PLoS Pathog 2017;**13**(2):e1006220. .
13. Chacón-Duque J, Adhikari K, Avendaño E, et al. African genetic ancestry is associated with a protective effect on Dengue severity in colombian populations. Infect Genet Evol 2014;**27**:89-95. .
14. Brady OJ, Gething PW, Bhatt S, et al. Refining the global spatial limits of dengue virus transmission by evidence-based consensus. PLoS neglected tropical diseases 2012;**6**(8):e1760.
15. Collenberg E, Ouedraogo T, Ganame J, et al. Seroprevalence of six different viruses among pregnant women and blood donors in rural and urban Burkina Faso: A comparative analysis. J Med Virol 2006;**78**(5):683-92.
16. Ridde V, Agier I, Bonnet E, et al. Presence of three dengue serotypes in Ouagadougou (Burkina Faso): research and public health implications. Infect Dis Poverty 2016;**5**(23).
17. Caron M, Grard G, Paupy C, et al. First evidence of simultaneous circulation of three different dengue virus serotypes in Africa. PloS one 2013;**8**(10):e78030.
18. Leroy EM, Nkoghe D, Ollomo B, et al. Concurrent chikungunya and dengue virus infections during simultaneous outbreaks, Gabon, 2007. Emerging infectious diseases 2009;**15**(4):591-3.

For peer review only - http://bmjopen.bmj.com/site/about/guidelines.xhtml

19. Caron M, Paupy C, Grard G, et al. Recent introduction and rapid dissemination of Chikungunya virus and Dengue virus serotype 2 associated with human and mosquito coinfections in Gabon, central Africa. Clinical infectious diseases : an official publication of the Infectious Diseases Society of America 2012;**55**(6):e45-53.
20. Gabor J, Schwarz N, Esen M, et al. Dengue and chikungunya seroprevalence in Gabonese infants prior to major outbreaks in 2007 and 2010: A sero-epidemiological study. Travel Med Infect Dis 2016.
21. Pourrut X, Nkoghe D, Gonzalez JP, et al. No evidence of dengue virus circulation in rural Gabon. Emerging infectious diseases 2011;**17**(8):1568-9.
22. Andries A, Duong V, Ngan C, et al. Field evaluation and impact on clinical management of a rapid diagnostic kit that detects dengue NS1, IgM and IgG. PLoS Negl Trop Dis 2012;**6**(12):e1993.
23. Lell B, Agnandji S, von Glasenapp I, et al. A randomized trial assessing the safety and immunogenicity of AS01 and AS02 adjuvanted RTS,S malaria vaccine candidates in children in Gabon. PLoS One 2009;**4**(10):e7611. .
24. Agnandji ST, Lell B, Soulanoudjingar SS, et al. First results of phase 3 trial of RTS,S/AS01 malaria vaccine in African children. N Engl J Med 2011;**365**(20):1863-75. .
25. Ochieng C AP, Vittor AY, Nyoka R, Gikunju S, Wachira C, Waiboci L, Umuro M, Kim AA, Nderitu L, Juma B, Montgomery JM, Breiman RF, Fields B. Seroprevalence of Infections with Dengue, Rift Valley Fever and Chikungunya Viruses in Kenya, 2007. PLoS One 2015;**10**(7).
26. Ellis EM, Neatherlin JC, Delorey M, et al. A household serosurvey to estimate the magnitude of a dengue outbreak in Mombasa, Kenya, 2013. PLoS Negl Trop Dis 2015;**9**(4):e0003733.
27. United Nations Development Programme. Population, urban (% of population). In: Reports HD, ed.: UNDP, 2013.
28. United Nations. World Urbanization Prospects: The 2014 Revision, Highlights. In: Department of Economic and Social Affairs PD, ed. United Nations: United Nations, 2014.
29. Central Intelligence Agency. Age structure. The world factbook Central Intelligence Agency.
30. Ramharter M, Adegnika AA, Agnandji ST, et al. History and perspectives of medical research at the Albert Schweitzer Hospital in Lambaréné, Gabon. The Middle European Journal of Medicine, WIENER KLINISCHE WOCHENSCHRIFT 2007;**119**(Suppl 3):8–12.
31. Schwarz NG, Gysels M, Pell C, et al. Reasons for non-adherence to vaccination at mother and child care clinics (MCCs) in Lambaréné, Gabon. Vaccine 2009;**27**(2009):5371-75.
32. Kenya. Secondary Kenya. https://en.wikipedia.org/w/index.php?title=Kenya&oldid=737329303
33. Wichmann O, Yoon I, Vong S, et al. Dengue in Thailand and Cambodia: An assessment of the degree of underestimated disease burden based on reported cases. . PLoS NTD 2010.
34. Gibbons C, Mangen M, Plass D, et al. Measuring underreporting and under-ascertainment in infectious disease datasets: a comparison of methods. BMC public health 2014;**14**(147).
35. European Centre for Disease Prevention and Control (ECDC). Data quality monitoring and surveillance system evaluation - A handbook of methods and applications. Stockholm: European Centre for Disease Prevention and Control (ECDC), 2014.

For peer review only - http://bmjopen.bmj.com/site/about/guidelines.xhtml

36. Mease L, Coldren R, Musila L, et al. Seroprevalence and distribution of arboviral infections among rural Kenyan adults: a cross-sectional study. Virol J 2011;**8**(371).
37. World Health Organization. Guidelines for the clinical evaluation of dengue vaccines in endemic areas. Geneva: World Health Organization, 2008.
38. Alm E, Lindegren G, Falk K, et al. One-step real-time RT-PCR assays for serotyping dengue virus in clinical samples. BMC Infect Dis 2015;**15**(493).
39. Peeling R, Artsob H, Pelegrino J, et al. Evaluation of diagnostic tests: dengue. Nature Reviews Microbiology 2010:S30-S37
40. Kraus A, Messer W, Haymore L, et al. Comparison of plaque- and flow cytometry-based methods for measuring dengue virus neutralization. J Clin Microbiol 2007;**45**(11):3777-80.
41. de Alwis R, de Silva A. Measuring antibody neutralization of dengue virus (DENV) using a flow cytometry-based technique. Methods Mol Biol 2014;**1138**:27-39.
42. Lambeth C, White L, Johnston R, et al. Flow cytometry-based assay for titrating dengue virus. J Clin Microbiol 2005;**43**(7):3267-72.
43. Cabral-Castro M, Saramago Peralta R, Cavalcanti M, et al. A Luminex-based single DNA fragment amplification assay as a practical tool for detecting and serotyping dengue virus. Journal of virological methods 2016;**236**:18-24.
44. Balasuriya U, Shi P, Wong S, et al. Detection of antibodies to West Nile virus in equine sera using microsphere immunoassay. J Vet Diagn Invest 2006;**18**(4):392-5.
45. Beck C, Desprès P, Paulous S, et al. A High-Performance Multiplex Immunoassay for Serodiagnosis of Flavivirus-Associated Neurological Diseases in Horses. Biomed Res Int 2015;**2015**(678084).
46. Hadinegoro SRS. The revised WHO dengue case classification: does the system need to be modified? Paediatr Int Child Health 2012;**32**(S1):33-38.
47. Guilarde AO, Turchi MD, Siqueira Jr JB, et al. Dengue and Dengue Hemorrhagic Fever among Adults: Clinical Outcomes Related to Viremia, Serotypes, and Antibody Response. The Journal of Infectious Diseases;**197**(6): 817-24. .
48. Mossel E, Crabtree M, Mutebi J, et al. Arboviruses Isolated From Mosquitoes Collected in Uganda, 2008-2012. J Med Entomol 2017;**54**(5):1403-09. .
49. Shope R, al. e. *The Flaviviruses: Detection, Diagnosis and Vaccine Development*: Academic Press, 2003.
50. Nikolay B, Diallo M, Boye C, et al. Usutu virus in Africa. Vector Borne Zoonotic Dis 2011;**11**(11):1417-23.

For peer review only - http://bmjopen.bmj.com/site/about/guidelines.xhtml

Fig. 1 Description of the study components, including passive facility-based fever surveillance, healthcare utilization surveys, cost-of-illness surveys, and serological surveys

There are two arms in the study package, composed of four parts. In the health facility-based arm of the study package, there are the passive facility-based fever surveillance and cost-of-illness survey embedded within the surveillance. In the community arm of the study, there are serological survey and healthcare utilization survey.

For peer review only

For peer review only - http://bmjopen.bmj.com/site/about/guidelines.xhtml

Fig. 2 Map of the study area in Ouagadougou, Burkina Faso

Fig. 3 Map of the study area in Lambaréné, Gabon

Fig. 4 Map of the study area in Mombasa, Kenya

Figures 2 – 4 show the map of the study area at each site in Burkina Faso, Gabon, and Kenya.

For peer review only

For peer review only - http://bmjopen.bmj.com/site/about/guidelines.xhtml

Fig. 5 Patient flow in the fever surveillance

Eligible febrile patients identified and enrolled as study subjects will follow these steps to complete participation in the passive fever surveillance.

For peer review only

For peer review only - http://bmjopen.bmj.com/site/about/guidelines.xhtml

Fig. 6 Laboratory testing algorithm for dengue

Samples from subjects of the passive fever surveillance would follow these steps of the testing algorithm for confirmation of dengue.

For peer review only

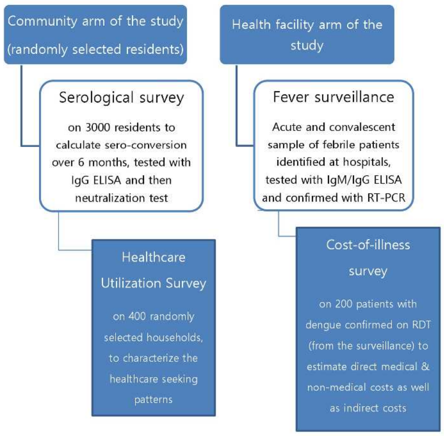

Fig. 1 Description of study components, including passive facility-based fever surveillance, healthcare utilization surveys, cost-of-illness surveys, and serological surveys

210x297mm (300 x 300 DPI)

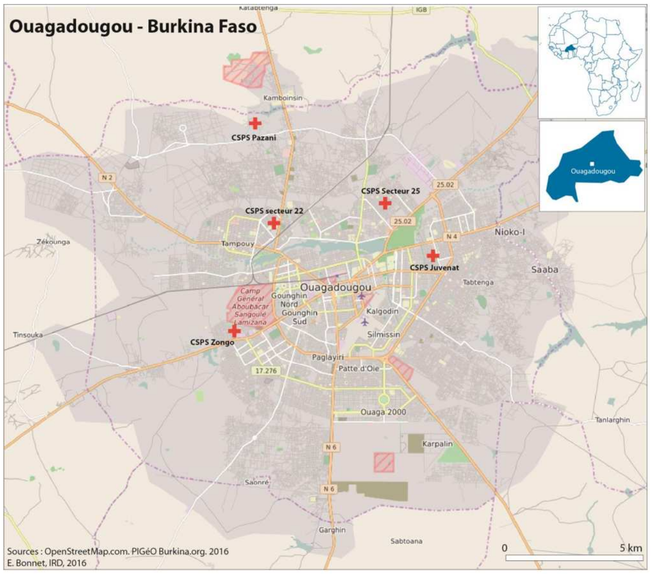

Fig. 2 Map of the study area in Ouagadougou, Burkina Faso

A map of the city and distribution of the population in Ouagadougou, Burkina Faso

140x198mm (300 x 300 DPI)

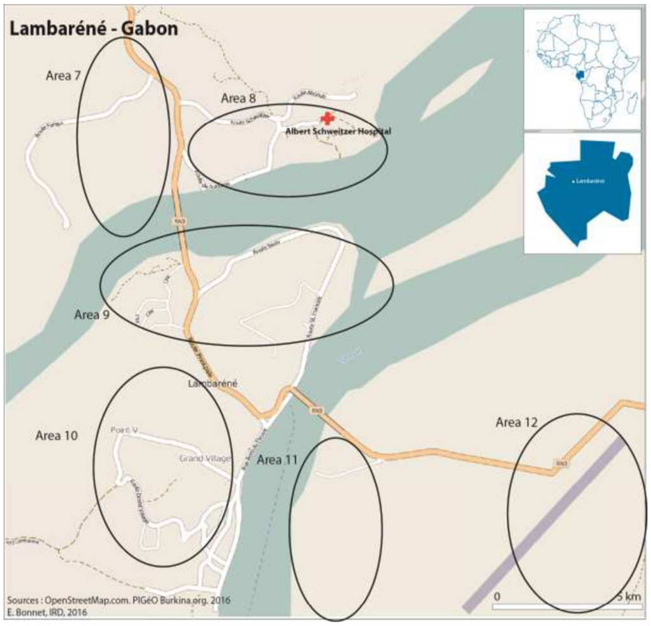

A map of the city and distribution of the population in Lambarene, Gabon

140x198mm (300 x 300 DPI)

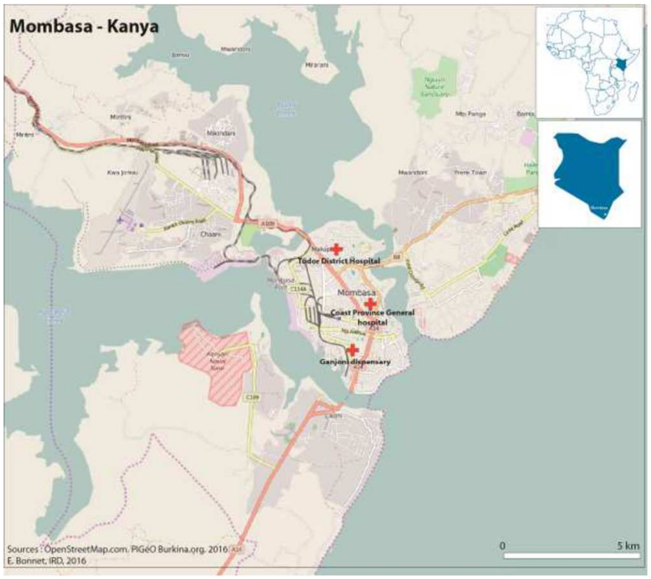

A map of the city and distribution of the population in Mombasa, Kenya

140x198mm (300 x 300 DPI)

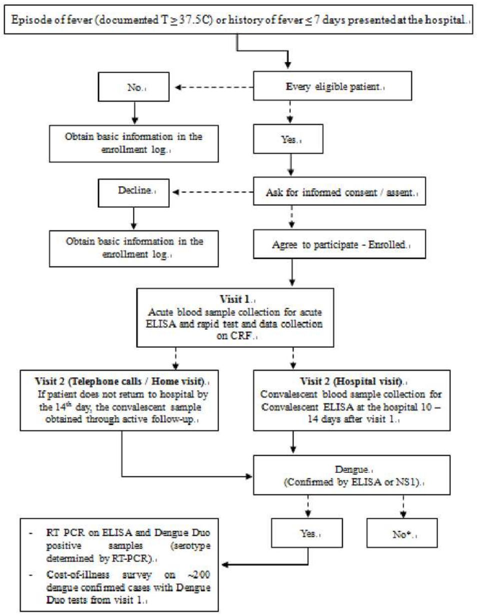

* A small number of those samples that are negative on ELISA or NS1 are tested with PCR to exclude false negative results of the ELISA.

Patient flow in the fever surveillance - Eligible febrile patients identified and enrolled as study subjects will follow these steps to complete participation in the passive fever surveillance.

140x198mm (300 x 300 DPI)

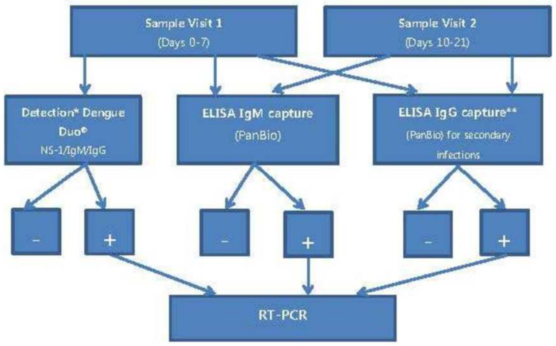

Fig. 6 Laboratory testing algorithm for dengue

* Dengue Duo® test is performed on enrolled febrile patients to identify dengue cases for immediate follow-up of dengue-confirmed cases in the cost-of-illness survey.

**Selected samples, including those that were found positive by IgM and NS1 on Dengue Duo®, as well as those positive by IgM and IgG capture ELISA, will be tested with RT-PCR.

Laboratory testing algorithm for dengue: Samples from subjects of the passive fever surveillance would follow these steps of the testing algorithm for confirmation of dengue.

140x198mm (300 x 300 DPI)

BMJ Open

Evaluating dengue burden in Africa in passive fever surveillance and sero-prevalence studies: protocol of field studies of the Dengue Vaccine Initiative

Journal:	*BMJ Open*
Manuscript ID	bmjopen-2017-017673.R3
Article Type:	Protocol
Date Submitted by the Author:	25-Sep-2017
Complete List of Authors:	Lim, Jacqueline; International Vaccine Institute, Global Dengue and Aedes-transmitted Diseases Consortium; London School of Hygiene and Tropical Medicine Faculty of Epidemiology and Population Health<br>Carabali, Mabel; International Vaccine Institute, Global Dengue and Aedes-transmitted Diseases Consortium<br>Lee, Jung-Seok; International Vaccine Institute, Development and Delivery<br>Lee, Kang Sung ; International Vaccine Institute, Development and Delivery<br>Namkung, Suk; International Vaccine Institute, Global Dengue and Aedes-transmitted Diseases Consortium<br>Lim, Sl-Ki; International Vaccine Institute, Global Dengue and Aedes-transmitted Diseases Consortium<br>Ridde, Valéry; Universite de Montreal Ecole de Sante Publique, ESPUM; Université de Montréal, Département de médecine sociale et préventive<br>Fernandes, Jose; Centre de Recherches Médicales de Lambaréné, Fondation Internationale de l'Hôpital Albert Schweitzer<br>Lell, Bertrand; Centre de Recherches Médicales de Lambaréné, Fondation Internationale de l'Hôpital Albert Schweitzer<br>Matendechero, Sultani ; Ministry of Health, Kenya, Communicable Disease Prevention and Control<br>Esen, Meral; University of Tübingen, Institute of Tropical Medicine<br>Andia, Esther; Kenya Medical Research Institute, Eastern and Southern Africa Centre of International Parasite Control (ESACIPAC)<br>Oyembo, Noah; Kenya Medical Research Institute, Eastern and Southern Africa Centre of International Parasite Control (ESACIPAC)<br>Barro, Ahmed; Action-Gouvernance-Integration-Reinforcement, Program Equité<br>Bonnet, Emmanuel ; Institut de recherche pour le developpement<br>Njenga, Sammy; Kenya Medical Research Institute, Eastern & Southern Africa Centre of International Parasite Control (ESACIPAC)<br>Agnandji, Selidji ; Centre de Recherches Médicales de Lambaréné, Fondation Internationale de l'Hôpital Albert Schweitzer<br>Yaro, Seydou; Centre MURAZ<br>Alexander, Neal; London School of Hygiene and Tropical Medicine Faculty of Epidemiology and Population Health<br>Yoon, In Kyu; International Vaccine Institute, Global Dengue and Aedes-transmitted Diseases Consortium
**Primary Subject Heading**:	Infectious diseases

Secondary Subject Heading:	Infectious diseases, Epidemiology, Global health, Public health
Keywords:	dengue, Epidemiology < INFECTIOUS DISEASES, Public health < INFECTIOUS DISEASES, Tropical medicine < INFECTIOUS DISEASES

SCHOLARONE™ Manuscripts

For peer review only

Evaluating dengue burden in Africa in passive fever surveillance and sero-prevalence studies: protocol of field studies of the Dengue Vaccine Initiative

Jacqueline K. Lim[1 11*], Mabel Carabali[1], Jung-Seok Lee[2], Kang Sung Lee[2], Suk Namkung[1], Sl-Ki Lim[1], Valery Ridde[3], Jose Fernandes[4], Bertrand Lell[4], Sultani Hadley Matendechero[5], Meral Esen[6], Esther Andia[7], Noah Oyembo[7], Ahmed Barro[8], Emmanuel Bonnet[9], Sammy M. Njenga[7], Selidji Todagbe Agnandji[4], Seydou Yaro[10], Neal Alexander[11], In Kyu Yoon[1]

[1]International Vaccine Institute, Global Dengue and Aedes-transmitted Diseases Consortium, Seoul, Korea, Republic of; [2]International Vaccine Institute, Development and Delivery, Seoul, Korea, Republic of; [3]Montreal School of Public Health (ESPUM), Montreal, QC, Canada; [4]Centre de Recherches Médicales de Lambaréné, Fondation Internationale de l'Hôpital Albert Schweitzer, B.P.242, Lambaréné, Gabon; [5]Communicable Disease Prevention and Control, Ministry of Health, Nairobi, Kenya; [6]Institute of Tropical Medicine, University of Tübingen, Germany; [7]Eastern and Southern Africa Centre of International Parasite Control (ESACIPAC), Kenya Medical Research Institute (KEMRI), Nairobi, Kenya; [8]Action-Gouvernance-Integration-Reinforcement (AGIR), Program Equité, Ouagadougou, Burkina Faso, [9]Institut de Recherche pour le Développement (IRD), Paris, France; [10]Centre Muraz, Bobo-Dioulasso, Burkina Faso; [11]Faculty of Epidemiology and Population Health, London School of Hygiene and Tropical Medicine

*Corresponding author
Jacqueline K. Lim[1*], MPH
Research Scientist
Global Dengue and Aedes-transmitted Diseases Consortium (GDAC)
International Vaccine Institute (IVI)
SNU Research Park, Gwankak-ro 1
Gwanak-gu, Seoul, Korea 151-191
T: +82-2-881-1340 (Dir) / +82-2-872-2801 (ext. 340)
Email: kalim@ivi.int

37 pages
Word count of the text: 6,516 (not counting the Abstract and table 1)
Word count of the abstract: 319
Figures and tables: 6 figures and 1 table

For peer review only - http://bmjopen.bmj.com/site/about/guidelines.xhtml

Abstract

Introduction.

Dengue is an important and well-documented public health problem in the Asia-Pacific and Latin American regions. However, in Africa, information on disease burden is limited to case reports and reports of sporadic outbreaks, thus hindering the implementation of public health actions for disease control. To gather evidence on the undocumented burden of dengue in Africa, epidemiological studies with standardized methods were launched in three locations in Africa.

Methods and Analysis.

In 2014-17, the Dengue Vaccine Initiative (DVI) initiated field studies at three sites in Ouagadougou, Burkina Faso; Lambaréné, Gabon; and Mombasa, Kenya; to obtain comparable incidence data on dengue and assess its burden through standardized hospital-based surveillance and community-based serological methods. Multidisciplinary measurements of the burden of dengue were obtained through field studies that included passive facility-based fever surveillance, cost-of-illness surveys, serological surveys, and healthcare utilization surveys. All three sites conducted case detection using standardized procedures with uniform laboratory assays to diagnose dengue. Healthcare utilization surveys were conducted to adjust population denominators in incidence calculations for differing healthcare seeking patterns. The fever surveillance data will allow calculation of age-specific incidence rates and comparison of symptomatic presentation between dengue and non-dengue patients using multivariable logistic regression. Serological surveys assessed changes in immune status of cohorts of approximately 3,000 randomly selected residents at each site at 6-month intervals. The age-stratified serosurvey data will allow calculation of seroprevalence and force of infection of dengue. Cost-of-illness evaluations were conducted among patients with acute dengue by Rapid Diagnostic Test.

Ethics and Dissemination.

The protocol for each study obtained ethical approvals from the Institutional Review Boards of the International Vaccine Institute, the London School of Hygiene and Tropical Medicine, and the Ethics Committee of host country institutions. By standardizing methods to evaluate dengue burden across several sites in Africa, these studies will generate evidence for dengue burden in Africa and data will be disseminated as publication in peer-review journals by the end of 2017.

For peer review only

Strengths of this study

- There have not been population-based studies conducted with a multi-disciplinary approach (i.e. surveillance, healthcare utilization, and sero-survey in one catchment area population). Data from the passive surveillance will be used to calculate annual incidences of dengue and data from the serosurvey will estimate the force of infection and prevalence.
- The studies were conducted in three locations in Africa, based on standardized methods and laboratory algorithm. Thus, comparison by site would be possible.

Limitations of this study

For peer review only - http://bmjopen.bmj.com/site/about/guidelines.xhtml

- This is not a cohort study. The passive facility-based surveillance may lead to under-estimation of the burden of dengue fever by measuring incidence based on only those that sought care at our study facilities.
- There may be limited generalizability of our study results to other dengue-endemic parts of Africa.

Keywords: dengue; Africa; seroepidemiologic Studies; incidence

Running head: Evaluating dengue burden in Africa: DVI field studies

For peer review only

For peer review only - http://bmjopen.bmj.com/site/about/guidelines.xhtml

Background

Dengue fever, a mosquito-borne flavivirus infection caused by four related but antigenically distinct dengue viruses (DENVs, serotypes 1–4), is a major and rapidly increasing global public health problem. Recent studies have estimated an annual incidence of 50–100 million symptomatic infections globally [1]. Dengue is a high burden disease that disproportionately affects countries in the tropics and subtropics, many of which have limited health care resources [2]. Although one dengue vaccine has been recently licensed in several endemic countries, the vaccine has restricted age and epidemiological indications. Other prevention and control measures such as vector control are suboptimal as stand-alone interventions [3 4], and no drugs for treatment are currently available.

Like in Asia and the Americas, epidemics of dengue were reported from Africa in the late 19th and early 20th centuries[5 6]. Specifically for Africa, there are records of multiple dengue case reports between 1964 and 1968 with DENV 2 in Nigeria[7]. Data from several studies conducted in the 1960-70s in Nigeria supported a substantially high level of immunity in adults as well as children [8 9]. In 2011, Amarasinghe et al. conducted a comprehensive review of literature on dengue in Africa and described that dengue cases have been reported in 34 countries in Africa, with most of these countries also having *Aedes* mosquitoes [6]. However, prior studies which suggested the presence of dengue in Africa were limited by their retrospective design or sample collection (blood donors or sample collected from surveys of other diseases), and often from travellers, with a small number of reported autochthonous cases, to demonstrate the true, population-based, burden of dengue. Also, while many dengue endemic countries in Asia and Latin America have mandatory reporting of dengue cases to public health authorities, and national surveillance systems in place to monitor incidence patterns [10], most African countries lack such established reporting

For peer review only - http://bmjopen.bmj.com/site/about/guidelines.xhtml

mechanisms and only sporadic outbreaks and individual case reports have been documented. In addition, the frequently non-specific clinical presentation of dengue may be difficult to distinguish from the myriad other infectious diseases present in Africa, since dengue diagnostic assays are not widely available. Thus, the burden of dengue remains largely unknown in Africa [6 11]. Without such dengue burden data, informed decision-making about prevention and control measures, including dengue vaccine introduction, in Africa are not possible.

Limited by surveillance capacity hindering continuous reporting in the region, there had not been frequent and systematic reporting of dengue in Africa. African ancestry is known to be protective against severe dengue and the candidate genes were recently identified in a Cuban patient [12 13]. Bhatt et al.'s modelling of the global dengue burden suggests high burden in Africa in terms of equal numbers of infections (both apparent and inapparent) as in Latin America[1]. There are new findings about dengue in Africa, but there is still much unknown about the magnitude of the dengue problem in the continent. To improve estimates of population-based dengue disease burden in Africa and validate whether the undocumented burden of dengue is as high in Africa as in the Americas with empirical data, the Dengue Vaccine Initiative (DVI) initiated field studies at three sites in West, West-Central, and East Africa in Ouagadougou, Burkina Faso; Lambaréné, Gabon; and Mombasa, Kenya; respectively. In each of the three sites, a standardized package of study components, including passive facility-based fever surveillance, healthcare utilization surveys, cost-of-illness surveys, and serological surveys (Fig 1), was initiated between December 2014 and March 2016.

Methods

For peer review only - http://bmjopen.bmj.com/site/about/guidelines.xhtml

Site selection

Study sites were selected, in part, based on their likelihood of supporting DENV transmission. To select sites, we considered dengue outbreaks and cases reports in the literature, available seroprevalence studies, as well as country-specific dengue risk maps of the probability of DENV transmission and the level of evidence of dengue presence, reporting the uncertainty of the consensus estimates of dengue in Africa [7 14]. In addition, adequate research infrastructure to implement the studies was taken into account. Finally, inclusion of different regions of Africa was also a factor in site selection. Thus, Ouagadougou, Burkina Faso; Lambaréné, Gabon; and Mombasa, Kenya; were selected, respectively, to measure the burden of dengue in selected sites from West, (West-) Central, and East Africa.

In Burkina Faso, the first reported dengue outbreak occurred in Ouagadougou in 1982 due to DENV-2 [6]. Serological prevalence of dengue antibodies among pregnant women and blood donors was found to be 26.3% in a rural setting (Nouna village) and 36.5% in an urban setting (Ouagadougou) in 2006 [15]. More recently, an observational study conducted by Ridde et al. among febrile patients consulting at selected study facilities in 2013-14 showed 8.7 % (33/379) to be positive by dengue RDT; and 15 of 60 samples tested by RT-PCR to be dengue positive [16]. With evidence for the presence of dengue, along with a strong health and demographic surveillance system (Ouaga-HDSS) which could be used to describe the catchment area, a field study was initiated in Ouagadougou, Burkina Faso in December 2014.

In Gabon, cases of dengue hemorrhagic fever (DHF) caused by up to three different DENV serotypes have been reported, and dengue seroprevalence has been found to be between 5 and 20% [17-19]. Results of a recently published study demonstrated seroprevalence of 12.3% among toddlers approximately 30 months of age in semi-rural Lambaréné between 2007 and 2010 [20]. However, a different study in 2005-2008 suggested minimal DENV

For peer review only - http://bmjopen.bmj.com/site/about/guidelines.xhtml

transmission in rural areas of Gabon [21]. This latter study examined antibodies against dengue in individuals from randomly selected villages representing about 10% of all Gabonese villages. Blood samples were tested by anti-DENV IgG and IgM capture ELISA and found to have only minimal IgG (0.5%) and IgM (0.5%) seroprevalence. Based on these low prevalences, the authors concluded that there was no active circulation of DENV in rural Gabon. However, the low seroprevalence may have been affected by low sensitivities of the tests used, leading to a high rate of false negative, and/or selection bias in the blood sample pool among the selected villagers [22]. Seroprevalence estimates in the 2007/2010 study may have also been impacted by the possibility of false-positive results due to IgG cross-reactivity among flaviviruses [21]. Nevertheless, given the possibility of DENV circulation in Gabon, a field study was initiated in Lambaréné in March 2015 in a community with a catchment population of about 77,000 residents, using the clinical research infrastructure of the Centre de Recherches Medicales de Lambaréné (CERMEL), benefiting from experienced research staff who conducted a large Phase 3 malaria vaccine trial [23 24].

In Kenya, more evidence is available for the presence of dengue based on local data. Dengue was the most common viral pathogen in retrospectively tested blood specimens from HIV-negative survey samples from the 2007 Kenya AIDS Indicator Survey. Antibody testing for dengue as well as chikungunya and Rift Valley fever was performed by IgG ELISA using either commercial kits or CDC assays; 12.5% were found to be dengue positive [25]. Similarly, a household survey found 13% of individuals from 701 households in Mombasa had serological evidence of either past or current DENV infection [26]. These data suggest that there is more dengue in Kenya than indicated by public health reporting, possibly due to misdiagnosis [25 26]. A field study was initiated in Mombasa, Kenya in March 2016.

Study participants

For peer review only - http://bmjopen.bmj.com/site/about/guidelines.xhtml

For the passive facility-based fever surveillance, individuals who met the following criteria were eligible for study enrollment:

1. Age 1- 55 years old;
2. Resident of the catchment area covered by healthcare facilities participating in the study, without plans to move out of the catchment area within 12 months;
3. Signed informed consent, and assent for those aged between 7 and 18 years; and
4. Patients presenting with current fever (axillary temperature ≥ 37.5º C) or history of fever for ≤ 7 days duration without localizing signs (fever caused by a localized infection as well as fever with a known and confirmed etiology other than dengue, such as malaria confirmed by malaria RDT- listed in the patient identification SOP).

For the serological survey, criteria 1-3 were applied. For the healthcare utilization survey, household interviews were conducted among the heads or representatives of the household invited from each family participating in the serosurvey.

Study area and population

Burkina Faso, located in West Africa, has a population of 14,017,462. The country is mainly rural with about 29% of the population reported to be living in urban areas in 2014. However, Burkina Faso is urbanizing rapidly and is positioned as the country with the fourth fastest urbanization in the last 25 years [27 28]. The capital, Ouagadougou, has a population of 2,741,128. The majority of the population live in urban settings. About 45% of the population are under 15 years of age [29]. The city is divided into 12 districts and 52 sectors. Ouagadougou is the country's largest city and the cultural and economic center. The city is part of the Soudano-Sahelian area, with a rainfall of about 800 mm per year. The rainy season is from

For peer review only - http://bmjopen.bmj.com/site/about/guidelines.xhtml

May to October, with a mean temperature of 28 °C (82 °F). The cold season runs from December to January, with a minimum average temperature of 16 °C (61 °F). During the hot season, which runs from March to May, the temperature can reach as high as 43 °C (109 °F).

The Health and Demographic Surveillance System is in place in Ouagadougou. Ouaga-HDSS monitors a population of 81,717 residents; according to this surveillance system, the city population is very stable with a rate of migration of 4.1% and more than 80% of the inhabitants with ownership of their houses [20]. A map of the city and distribution of the population is shown in Figure 2.

Gabon, located on the west coast of Central Africa, has an area of nearly 270,000 square kilometres (100,000 sq. mi) with a population estimated at 1.5 million. Its capital and largest city is Libreville. In 2014, it is reported that 87% of the Gabonese population lived in urban areas [28]. The sixth largest city, Lambaréné, the capital of Moyen-Ogooué province, is located 75 kilometers south of the equator, with a population of 25,257 in 2009. The majority of Lambaréné residents live in semi-rural areas. About 42% of the Gabonese population is under 15 years of age [29]. Similarly, Lambaréné's population is relatively young with about 50% under 20 years of age.

The health services of Gabon are mostly public, but there are some private institutions as well. With one of the best medical infrastructure in the region, almost 90% of the population have access to health care services. Albert Schweitzer Hospital (ASH) is a private institution which served as a study site for the passive fever surveillance study [30 31]. The study area in Lambaréné is shown in Figure 3.

Kenya, located in East Africa, lies on the equator, covering 581,309 km^2 (224,445 sq. mi), with a population of approximately 45 million people in 2014 [2]. Kenya generally has a warm and humid tropical climate but is diverse, ranging from the cooler

For peer review only - http://bmjopen.bmj.com/site/about/guidelines.xhtml

climate around the capital city, Nairobi, to a hot and dry climate inland, as well as a desert-like climate in the north-eastern regions along the border with Somalia and Ethiopia [32]. The capital, Nairobi, is a regional commercial hub. The main industries include agriculture, exporting tea and coffee, as well as the service industry.

Kenya is divided into 47 semi-autonomous counties. Mombasa is the country's second largest city after Nairobi and is located on the east coast of the country [2]. Administratively, Mombasa is the capital of Mombasa County, which was formerly called Coast Province. This overall Coast region covers over 80,000 km^2 in the south-eastern part of Kenya, constituting about 15% of the country's land area, with a population of 3,325,307 residents.

The main economic driver of Mombasa is tourism and trading industry. Mombasa itself has a population of about 1.3 million with almost 50% of the population under 15 years of age [29]. Increasingly, the population of the province lives in urban areas; at present about 45% live in Mombasa and other urban centers. The "long rains" period begins around April and the "short rains" period begins in October [32]. Mean annual temperature ranges from 24°C to 27°C, but maximum temperature averages over 30°C during the hottest months, January to April.

Figure 4 shows the area of Mvita subcounty of Mombasa, which was the catchment area for the study in Kenya, with a catchment population of 74,735 residents. The map indicates the three facilities involved in the study.

Sample size

Given the paucity of available age-specific dengue incidence data in the study countries or nearby countries, it was difficult to obtain population-based incidence to make assumptions when calculating sample sizes. The required catchment population for the

For peer review only

passive facility-based fever surveillance was roughly estimated based on the limited data available in the literature. Annual incidence estimates were calculated based on available prevalence estimates with the assumption that the outcome of interest has zero prevalence at age zero, and that force of infection is constant. It was assumed that prevalence estimates found for one particular age group would be adjusted as the annual incidence and used across all ages.

Wichmann et al. calculated an expansion factor for children by comparing data from three cohort studies to national surveillance data in Southeast Asia [33]. For children in Thailand, the age-specific expansion factors calculated were 11.85 for <5 years, 8.76 for 5-9 years, and 7.81 for 10-14 years [33]. The results show that, even for Asia where better reporting and surveillance systems are available, there is a considerable degree of underreporting. For Africa, there may be more dengue cases under-ascertained (not seeking care) and under-reported (not reported even if a patient with dengue seeks care, given that dengue is not one of the routinely notifiable diseases in Africa), but such information on the extent of underestimation of dengue was not available [34 35]. Also, the incidence estimates used in our sample size calculations were not from population-based studies. While it would have been ideal to adjust the incidence further for likely underestimation, the annual incidence used in sample size calculations could not be adjusted for possible under-reporting due to the lack of data. The sample sizes were calculated with 95% confidence levels and a margin of error at a fixed significance level within 25% of the true proportion of incidence. This gives relative precision of 75%, considering the gap in evidence for dengue incidence in the study areas. The final sample sizes were calculated by assuming 20% non-response rate or loss to follow-up. The required catchment population size for the fever surveillance study in Burkina Faso was estimated to be 100,000, Gabon to be 77,000, and Kenya to be 70,000. In these catchment populations, the number of enrolled subjects depends on the number of eligible

For peer review only - http://bmjopen.bmj.com/site/about/guidelines.xhtml

patients who seek care at the study facilities. How many eligible febrile episodes would actually present at our study facilities was difficult to predict; but after assessment of the volume of febrile patients at the facilities, a realistic upper limit for enrollment for a study period of approximately 1.5 years was set at 3,000 subjects to offer enrollment to all consenting eligible patients.

For the serological survey, the sample size was calculated similarly using the prevalence proportion based on published literature. Seroprevalence of 0.304 for Burkina Faso [15], 0.123 for Gabon [21], and 0.144 for Kenya [36] were used. With the same confidence levels and allowed margin of error, and assuming 10-30% (variable by site) non-response rate, the sample size was calculated to be 3,000 participants at each site. Again, with the scarcity of data from the selected countries, there were no other prevalence estimates reported or estimates from different age groups. As prevalence is expected to increase with age, and higher prevalence would give a smaller sample size, our calculations are likely to be conservative.

Study components

Fever surveillance – design and methods

To determine burden due to symptomatic dengue in each of the three sites in Burkina Faso, Gabon, and Kenya, passive facility-based fever surveillance was implemented in a well-defined catchment area population. In Burkina Faso, the surveillance study was initiated in December 2014 in five selected primary health care centres, locally called “Centre de Santé et de Promotion Sociale” (CSPS), in the municipality of Ouagadougou, with a catchment population of 105,000 residents. This project was implemented in collaboration with Centre Muraz in Bobo-Dioulasso, EQUITE sante program (a collaborative program

For peer review only - http://bmjopen.bmj.com/site/about/guidelines.xhtml

between University of Montreal and Action-Gouvernance-Integration-Reinforcement, AGIR, based in Ouagadougou, funded by the Canadian Institute of Health Research), and DVI. In Gabon, the surveillance study was initiated in the Albert Schweitzer Hospital serving a catchment population of 130,000 residents in the Moyen-Ogooué and surroundings within Lambaréné, in collaboration with CERMEL and Institute of Tropical Medicine in Tubingen, Germany. In Kenya, the surveillance study was implemented at Ganjoni dispensary, Tudor sub-county Hospital, and Coast Provincial General Hospital, serving a catchment population of 70,000 residents in Mombasa, in collaboration with Kenya Medical Research Institute (KEMRI) and Ministry of Health of Kenya.

As described in Figure 5, both outpatients and inpatients at the designated study facilities, who meet inclusion criteria as mentioned earlier are tested for dengue, first with SD Dengue Duo® RDT. Dengue confirmation is done by detection of dengue virus in serum samples using PCR, as well as anti-dengue IgM and IgG antibodies in acute and convalescent serum by ELISA (SD Dengue IgM & IgG capture ELISA® tests, Standard Diagnostics, Yongin-Si, Korea) [10 37]. Every consecutive patient meeting inclusion criteria is eligible for enrolment during the study period. Infants < 1 year old were not included due to operational limitations, such as difficulty of infantile bleeding.

In Ouagadougou, Burkina Faso, the fever surveillance was initiated in December 2014 and continued until February 2017 (approximately 2 years). In Lambaréné, Gabon, the fever surveillance was initiated in April 2015 and continued until January 2017 (approximately 1.5 years). In Mombasa, Kenya, the fever surveillance was initiated in March 2016 was continued until June 2017.

Among subjects enrolled in the fever surveillance, those who are positive by dengue

For peer review only - http://bmjopen.bmj.com/site/about/guidelines.xhtml

rapid diagnostic test are offered further enrolment in the cost-of-illness survey, consisting of interviews on the day of acute illness visit, day 10-14 from the first visit, and day 28, if illness continues. The cost-of-illness survey questionnaire was designed to estimate the direct medical, direct non-medical, and indirect costs associated with dengue-positive patients identified at study facilities. This survey also estimates the cost of treating dengue at the facility level. Data are gathered by linking patients' medical records concerning outpatient visits, inpatient visits, and service consumption (e.g., diagnostic tests, medication, and other services provided to patients). The cost-of-illness portion of the study will be described separately.

Fever surveillance – laboratory testing

As shown in Figure 6, in all three sites, acute samples are tested using a commercial RDT for dengue NS1 and IgM/IgG (Dengue Duo®, Standard Diagnostics, Yongin-Si, Korea). Dengue Duo RDT is used on the day of acute illness visit at the site of patient presentation (day 1). The acute and convalescent samples are subsequently tested at a local laboratory using dengue IgM/IgG ELISA (SD Dengue IgM & IgG Capture ELISA®, Standard Diagnostics, Yongin-Si, Korea). The serum is separated and stored in 4 aliquots of about 500 μL for various laboratory tests, as indicated in consent documents.

After ELISA testing, samples will be shipped to the International Vaccine Institute (IVI) in Korea. Samples with positive results by RDT or ELISA undergo further testing by RT-PCR at the Clinical Immunology Laboratory of IVI. Four DENV serotype-specific real-time RT-PCR assays are used for laboratory confirmation of dengue, and serotyping [38]. The DENV 1-4 RT-PCR assays are carried out in 25μL reaction mixtures containing 5μL

For peer review only - http://bmjopen.bmj.com/site/about/guidelines.xhtml

template RNA, TagMan® Fast Virus 1-step mastermix (Applied Biosystems®), 0.9 μM of each primer, and 0.2 μM probe [38]. Amplification and detection are performed in a StepOne Plus real-time PCR system, and the baseline and threshold are determined using the auto-baseline and threshold feature in StepOne Software v2.2.2 (Applied Biosystems®). Thermocycling parameters are as follows: reverse transcription at 50 °C for 5 min, inactivation at 95 °C for 20 s, followed by 45 cycles of fluorescence detection at 95 °C for 3 s, and annealing at 60 °C for 30 s [38]. A specimen is considered positive if target amplification is recorded within 40 cycles.

Serological survey – design and methods

While the facility-based fever surveillance studies provide estimates of the burden of medically-attended dengue disease, evaluation of all DENV infections in a population – including subclinical and mildly symptomatic infections, which impact immune status – is needed to capture the overall impact of dengue. As part of the study package, population-based serological surveys were conducted in the same catchment population used for the fever surveillance. At each of the three sites in Africa, the serosurvey was conducted on a cohort of approximately 3,000 randomly selected residents of urban and semi-urban parts of Ouagadougou, Lambaréné, and Mombasa. Without individual-level census information on all residents of Lambaréné and Mombasa, with help of community/village health workers, randomization was done based on neighbourhoods (or defined areas for which the health workers/volunteers are responsible) as cluster units. As the community/village health workers are familiar with the villages and their residents, they are good entry points into the communities. With these health workers, the field team screened houses in the selected

For peer review only - http://bmjopen.bmj.com/site/about/guidelines.xhtml

villages by knocking on doors of every 5~7 houses, depending on the household density per neighbourhood. Also, demographic information collected in previous research projects conducted in the same area was used as a guide, if available. In the case of the site in Ouagadougou, HDSS data were available and the EQUITE SANTE, a CIHR funded research program of the University of Montreal, had set up a geographic information system (GIS) database of houses in the study area. Using these data, households of potential enrollees of the serosurvey were pre-selected randomly and household visits were made in Ouagadougou. In the three sites, about 45% of the serosurvey samples were targeted to be collected from children 1 - 14 years-of-age, and 55% were targeted to be collected from adults between 15 and 55 years of age to reflect the age distribution of the general population of the area. Household-based enrollment was offered to the head of the household until the specific cap for the age-group was reached in Lambaréné and Mombasa.

Randomly-selected subjects 1-55 years of age underwent phlebotomy (5ml for children and 7ml for adults) twice — before the rainy season and after the rainy season, at approximately 6-month intervals. The sera were evaluated using IgG indirect ELISA at baseline and after 6 months. The presence of dengue IgG antibodies at 6-month intervals will be used to estimate the level of occurrence of inapparent DENV infection and to calculate the rate of infection in the catchment population. Flow cytometry-based DENV neutralization assays will be applied to a subset of samples to assess for presence of dengue neutralizing antibodies and seroconversion over the 6-month interval. In addition to overall seroconversion, age-specific seroconversion estimates in the catchment population, as well as the proportion of inapparent infections, will be determined.

For peer review only - http://bmjopen.bmj.com/site/about/guidelines.xhtml

Serological survey – laboratory testing

From the samples collected in the serosurvey, about 200 μL of serum are used and tested at a local laboratory using dengue IgG ELISA (Panbio Dengue IgG Indirect ELISA®, Alere North America, LLC, Florida, United States). After ELISA testing for dengue IgG at the local laboratories, samples will be shipped to IVI. Given potential serological cross-reactivity among flaviviruses [39], flow cytometry-based neutralization assays will be performed against selected flaviviruses to include yellow fever virus, West Nile virus, Zika virus, and Japanese Encephalitis virus at the Clinical Immunology Lab of IVI [40 41]. About 50 samples per bleed for 4 bleeds in Burkina Faso and 2 bleeds in Gabon and Kenya will be tested.

About 1,000 μL of serum is allotted for this procedure. The flow cytometry-based neutralization assays are performed in duplicate in 96-well cell culture plates with flat-bottom wells, each containing DC-SIGN-expressing U937 cells [40]. The amount of virus used in the assay infects between 7 and 15% of the cells. Human immune sera are serially diluted and the virus is pre-incubated with the sera for 1 h at 37°C [40]. The cells are washed, and the virus and serum mixture is added to the cells for 1 h at 37°C, and the cells are further incubated for 24 to 48 h at 37°C in 5% CO_2. The cells are fixed, permeabilized, and stained with fluoresce-conjugated monoclonal antibody 4G2, which recognizes the flavivirus E protein [42]. FACScan flow cytometer (Becton Dickinson, San Diego, CA) is used to analyze the cells [40]. The serum dilution that neutralizes 50% of the viruses is calculated by nonlinear, dose-response regression analysis with Prism 4.0 software (GraphPad Software, Inc., San Diego, CA).

In addition, a Luminex-based multiplex immunoassay will be performed on a randomly selected sub-sample to assess for IgG to different flaviviruses [43]. About 200

For peer review only - http://bmjopen.bmj.com/site/about/guidelines.xhtml

samples per bleed for 4 bleeds in Burkina Faso and 2 bleeds in Gabon will be tested. Detection of IgG against ZIKV and each of the four DENV serotypes will be performed on patient serum samples using an in-house microsphere-based multiplex immuno-assay (arbo-MIA) at the Clinical Immunology Lab of IVI [44 45]. The arbo-MIA is based on a mixture of microspheres covalently coupled with either DENV-1, -2, -3, -4 or ZIKV recombinant antigens (E protein domain III) produced in Drosophila S2 expression system. Briefly, microsphere mixtures were sequentially incubated in the dark under constant shaking with a 1:400 dilution of patient serum samples, with 2 µg/mL anti-human IgG biotin-conjugated antibody (Jackson Immunoresearch, West Grove, PA) and with 2 µg/mL streptavidin-R-phycoerythrin conjugate (Life technologies). After the final incubation, the median fluorescence intensity (MFI) of each microsphere set is quantified using a BioPlex 200 instrument (Bio-Rad Laboratories, Hercules, CA). Samples are considered seropositive if the ratio of MFI values obtained for the viral antigen to the control antigen is superior to the defined cut-off. The cut-off of the MIA is determined for each viral antigen by ROC curve analysis using well characterized sera.

In Lambaréné, the enrolment bleed took place in November -December 2015, while the 2nd blood collection occurred in May 2016. In Ouagadougou, the enrolment bleed took place in May-June 2015 with follow-up blood collections in December 2015, June 2016, and January 2017. In Mombasa, the enrolment bleed took place in May 2016 with the 2nd blood collection in November 2016 – February 2017.

<u>Healthcare Utilization Survey</u>

As the passive fever surveillance is conducted at study facilities, potential dengue patients could be missed if they seek care elsewhere. To identify the proportion of fever and

For peer review only - http://bmjopen.bmj.com/site/about/guidelines.xhtml

dengue cases potentially missed by the passive surveillance system due to patients living in the study area but seeking care outside of study facilities, a population-based healthcare utilization survey was conducted in 400 randomly selected households from the study catchment area to characterize the healthcare utilization patterns of the households when they have (self-reported) febrile episodes among the family members. In addition to assessing health-seeking behaviours of the residents, preferences in terms of health-seeking behavior and respective reasons for their preferences were investigated. The questionnaire was administered to 400 heads of households. Among 3,000 residents who participated in the serosurvey, there were about 600 households. From these households, 400 heads of households were randomly selected and offered enrolment in the health utilization survey. Heads of households or a senior representative within the household were asked questions on health seeking patterns of their family members.

Study questionnaires

For the fever surveillance study, questionnaires are administered at the acute illness visit and the convalescent visit. The convalescent visit may take place at the health care facility (10-14 days later) or at the patient's home (15-21 days after the acute visit), according to patient preference and availability. The questionnaires are completed by medical staff of the study facilities, including demographic and clinical information (e.g., signs, symptoms, past medical history, treatments prescribed, and diagnoses). The same staff also complete the follow-up questionnaire at the convalescent visit within 21 days from the acute visit. Study nurses complete surveillance enrolment log. Lab technicians complete the lab section (mostly dengue-related diagnostics) and the forms are compiled by the study coordinator on site.

For the serosurvey component, questionnaires are administered at the household by trained field team staff at each serosurvey visit. Study nurses complete the questionnaire after a brief

For peer review only - http://bmjopen.bmj.com/site/about/guidelines.xhtml

physical and medical examination. At the follow-up visit(s) in about 6 months, the same staff make the household visits to complete the follow-up questionnaire. Enrolment log is maintained by the study coordinator on site.

Variables of the surveillance questionnaires

The variables collected are listed in table 1.

Table 1. List of variables collected in the passive fever surveillance data collection form

Topic	Description	Items
Basic information	Demographic and basic information about the patient and the treatment received	Type of treatment where patient is enrolled (IPD vs. OPD)<br>Date of fever onset, duration of fever<br>Current temperature<br>Tourniquet test results<br>Patient's address (district and village-level)<br>Date of visit, date of birth, age, and sex<br>Weight and height
General health condition	Current condition of the patient (self-report) and underlying diseases of the patient	How well the patient could handle daily activities<br>Pre-existing conditions
Signs and symptoms during this illness	A set of sign and symptoms that may be related to fever and dengue (DF and DHF)	Rash, fatigue, headache, retro-orbital pain, neck/ear pain, sore throat, breathing difficulty, cough, expectoration, gastrointestinal signs (Nausea/vomiting, diarrhea, abdominal pain, etc.), hemorrhagic signs (nose/gum bleeding, ecchymosis, petechiae, etc.), signs of shock (cyanosis, capillary refill), arthralgia, myalgia, loss of appetite, jaundice, etc.
Medical History:	Previous dengue-related or other flavivirus infection as well as vaccination history (self-report)	Previous dengue infection and related hospitalization<br>Previous infection to other commonly circulating arboviral infection in the area<br>Yellow fever vaccination history
Laboratory findings	Records from the routine laboratory tests widely used in	Platelet count, hematocrit, haemoglobin, leukocytes, neutrophils,

For peer review only - http://bmjopen.bmj.com/site/about/guidelines.xhtml

	clinical fever/dengue patient management, as part of the hospital care procedure	protein level, AST, ALT, urine test results, etc.
Clinical Diagnosis	Clinician's diagnosis with or without referring to the RDT	Diagnosis given by the physician based on clinical presentation after physical examination of the patient.
Dengue testing results	Results from the dengue tests, mainly RDTs for dengue as well as other commonly circulating arbovirus in the area	Dates of blood draw<br>Test results of the RDT<br>IgM/IgG capture ELISA results<br>PCR results (if available)
Treatment	Medicine(s) prescribed and the starting and end dates	Antibiotics, paracetamol, ibuprofen, aspirin, and others that may be site-specifically prescribed
Outcome	Outcome of this particular visit	Hospitalized, returned home, or referral
Hospitalization	Information collected only among hospitalized patients in the surveillance to record other severe signs and progression of illness	Admission and discharge diagnoses<br>Presence of haemorrhagic signs or shock syndrome
Hospital Charges	Expenses and hospital charges incurred by patient on the visit 1	Amount of the out of pocket payment by the patient or the family/or guardian<br>Breakdown of the hospital charges (laboratory, medication, admission-related charges)
Final outcome	Outcome of the patient's illness at the 2nd visit	Final diagnosis given for the patient outcome of illness<br>Completion of study participation (early termination and the reason, etc.)

Planned statistical analysis

From the fever surveillance data, incidence of symptomatic dengue among patients that seek health care at the study facilities will be calculated. Age-specific incidence rates in all the children and adults will be determined by referring to the size and distribution of the general population of the study area at the time of surveillance as the denominator in calculation of the incidence of symptomatic dengue cases. Each person residing in the study area is assumed to contribute 12 months of person time to the denominator. Although the

For peer review only - http://bmjopen.bmj.com/site/about/guidelines.xhtml

study areas all report a low migration rate, the in-migration is assumed to balance the out-migration of the population during the study period. Age-specific incidence of symptomatic dengue will be calculated by using age-specific denominators and the number of symptomatic dengue cases in eligible individuals as the numerator.

Using the data collected in the Healthcare Utilization Survey, the proportion of febrile cases missed by the passive surveillance system will be determined. Then using the proportion, the numerator will be further adjusted in recognition of those missed fever cases from the study area, which could have been dengue. Also, comparison will be made between those that agreed to participate and those that declined participation among the eligible potential enrollees. The enrollment log, which records basic information obtained during the screening process of potential enrollees, will be reviewed. In addition to checking that our sample of febrile cases is representative of febrile patients of the general population in the catchment area, refusal rates will be determined based on information in the log. Then, the refusal rates will be used to adjust the numerator.

SPSS software will be used for analysis of the fever surveillance data. Multivariable logistic regression will be used to compare confirmed dengue patients versus non-dengue febrile patients in terms of symptomatic presentation, based on signs and symptoms collected from all patients with laboratory-confirmed dengue by serology and RT-PCR, adjusting for possible confounders, such as age, days since onset of fever, primary vs. secondary infection, inpatient vs. outpatient, etc. Differences in symptomatic complex of DF (and DHF, if data allows) by age and serotype will be also determined using multivariable logistic regression.

As outpatient disease accounts for the greater part of dengue disease burden, clinical profile of individuals with DENV infection will be characterized by the type of treatment (hospitalized and outpatients), as well as by severity of the disease (severe vs. non-severe by the 2009 WHO criteria) [46]. Classification is determined after the course of illness is

For peer review only - http://bmjopen.bmj.com/site/about/guidelines.xhtml

completed (typically during the convalescent visit). Symptomatic dengue is classified as outpatient or hospitalized. Progression of dengue is recorded as DF, DHF I, DHF II, DHF III or DHF IV, and clinical patterns will be compared by the severity grade [46 47]. These will be compared to results obtained from other DVI studies in Latin America (Colombia) and Asia (Thailand, Vietnam, and Cambodia). Overall, comparisons will be made across Burkina Faso, Gabon, and Kenya.

With the age-stratified sera that reflect the age distribution of the general population of the country, the serological survey sampling strategy ensures sufficient subjects to obtain precise age-specific estimates of sero-positivity and sero-conversion of the catchment area population. The sero-conversion rate and change in the immune status will be determined by age group during the study period. The age-stratified serosurvey data will also allow calculation of the force of infection of dengue in the study population. After enrolment, there are subjects who drop out in the follow-up bleeds about 6 months later. Basic demographic information will be compared between those that completed participation and those with incomplete participation to check whether study subjects represent the catchment area population. Comparisons will be made among Burkina Faso, Gabon and Kenya.

Ethical considerations

To minimize inconvenience of the study to patients, clinicians and nurses were sensitized and trained regarding the study requirements and procedures in order for data collection to be integrated into routine patient care. The clinicians and nurses selected for the study receive coordinated support from study field staff throughout the study process. Written informed consent, and assent for participants 7 (13 for Kenya) -17 years of age, were obtained from patients by study staff. Study staff go through consent and assent documents

For peer review only - http://bmjopen.bmj.com/site/about/guidelines.xhtml

for short summary of the disease, detailed description of study procedures, and information on reimbursement. Patient data are documented in the study designated office; only the study staff have access to the data that are de-identified. Data are exclusively handled in the study office and stored safely in a protected database in the study office as well as on the DVI main server.

The protocol for each study obtained ethical approvals from the Institutional Review Boards (IRBs) of the International Vaccine Institute, the London School of Hygiene and Tropical Medicine, and the Ethics Committee of host country institutions, including KEMRI Scientific and Ethical Review Unit in Kenya, Gabon National Ethics Committee and Institutional Ethics Committee, Scientific Review Board of CERMEL in Gabon, and the IRB of Centre Hospitalier de l'Université de Montréal (CRCHUM) at University of Montreal, and the National Health Ethical Committee of Burkina Faso.

Discussion

Dengue cases have been detected since the 1960's in Africa, and there has been continued presence of *Aedes* vectors in the continent [5 7]. However, very few dengue studies have been conducted in Africa, and little evidence is based on population-based studies [6]. Compared to the volume of evidence from SE Asia and the Americas, there is critical data scarcity on dengue in Africa. Suspicion of substantial dengue burden in Africa is based on limited reports of outbreaks and a handful of sero-prevalence studies testing different viruses among samples that likely do not represent the general population. In the three countries selected for our field studies, somewhat more data are available, but are still very limited. In Burkina Faso, a recent observational study conducted in 2013 reported that 8.7% of the febrile patients showed positive results on dengue RDT [16]. In Gabon, one study suggested minimal DENV circulation in rural areas [21], while another study reported 12.3%

For peer review only - http://bmjopen.bmj.com/site/about/guidelines.xhtml

seroprevalence, by IgG antibodies against dengue, among toddlers 30 months of age in semi-rural parts of Lambaréné [20]. In Kenya, about 13% of the individuals in Mombasa have been reported to have evidence of past or current DENV infection by RT-PCR and IgM anti-dengue ELISA after the 2013 outbreak [26]. Despite the limited scope and generalizability of these studies, they suggest that there may be more dengue than previously appreciated due to underestimation and misdiagnosis [25 26].

These studies suggest the presence of dengue and some level of underlying sero-prevalence in the countries of our field studies. However, often these studies are limited by their retrospective design or sample collection (blood donors or sample collected from surveys of other diseases) to demonstrate the true, population-based, burden of dengue. We proposed to address this gap by population-based dengue surveillance and sero-prevalence studies in West, (West-) Central, and East Africa.

The present studies at three sites in Africa will provide important information on undocumented DENV circulation in Africa. Such data will help to strengthen the evidence base for dengue burden in Africa. Better defined disease burden data based on our studies could be used to assess the relative need for dengue prevention and control measures, such as whether a dengue vaccine would be a cost-effective public health intervention for countries in Africa. Clinical findings from our studies could also be used as a guide for dengue case detection and case management.

The studies have some important limitations. We recognize variability of dengue epidemiology over time and by region. Due to resource constraints, our studies are limited in terms of time frames and geographical extent These constraints may limit the generalizability of our studies.

One potential source of bias in estimating the incidence of symptomatic dengue is under-ascertainment due to the community residents with relevant symptoms seeking care

For peer review only - http://bmjopen.bmj.com/site/about/guidelines.xhtml

from other healthcare providers and facilities than the study facilities. As the study design remains passive surveillance, cases are ascertained only at our study facilities. By estimating the proportion of febrile patients seeking care elsewhere, as well as refusal rates among the potential enrollees that were screened for eligibility criteria, the degree of febrile patients missed by the study can be determined. Inverse probability weighting will be used to account for these potential subjects missed by the surveillance as adjustments in incidence calculation. Also, depending on the transmission volume of dengue or other co-circulating diseases with onset of fever, there may be patients that are diagnosed with other diseases and ruled out for dengue. Furthermore, with respect to dengue diagnostics for our serological surveys, there are other circulating flaviviruses in Africa leading to challenges in identifying antibodies to past dengue infections. While our testing plan assesses for some flaviviruses, others known to circulate in Africa, such as Banzi and Usutu viruses, are not part of the testing plan [48-50]. Due to resource limitations, serological testing will be limited to yellow fever virus, West Nile virus, Zika virus, and Japanese Encephalitis virus as well as DENV 1-4. Therefore, in some cases, it may be difficult to determine prior exposure to DENV versus other flaviviruses based on serological data. This cross-reactivity may lead to over-estimation of dengue force of infection

In addition, the sero-survey and healthcare utilization survey are conducted on a randomized sub-sample of the catchment area population and there may be limited generalizability of the data collected from these surveys. With unknown differences among those that agree to participate and those that do not agree, the data may not be representative of the general population of the study countries.

Conclusions

For peer review only

For peer review only - http://bmjopen.bmj.com/site/about/guidelines.xhtml

The data collected from our studies will contribute to the assessment of the unknown dengue disease burden in Burkina Faso, Gabon, and Kenya. These data can fill a gap in undocumented burden of dengue in the region and, collectively, may be used to infer dengue burden in other areas of Western, Central, and Eastern Africa. Countries in Africa may not consider introduction of a dengue vaccine as a priority in the near future due to many other competing public health problems and limited resources. For cost-effective implementation of public health interventions, accurate data on dengue burden from epidemiological studies would be needed for policy makers to make evidence-based decisions on control and prevention of dengue. Our studies will provide some much needed information based on population-based research to assess dengue burden in Africa.

For peer review only

For peer review only - http://bmjopen.bmj.com/site/about/guidelines.xhtml

List of abbreviations

GDAC - Global Dengue and *Aedes*-transmitted Diseases Consortium
IVI - International Vaccine Institute
DENV- dengue viruses
DVI - Dengue Vaccine Initiative
Ouaga-HDSS - health and demographic surveillance system
DHF - dengue hemorrhagic fever
CERMEL - Centre de Recherches Médicales de Lambaréné
ASH – Albert Schweitzer Hospital
CSPS - Centre de Santé et de Promotion Sociale
KEMRI - Kenya Medical Research Institute
SD – Standard Diagnostics
GIS = geographic information system
MFI - median fluorescence intensity
CRCHUM - Centre Hospitalier de l'Université de Montréal

For peer review only

For peer review only - http://bmjopen.bmj.com/site/about/guidelines.xhtml

Declarations

- Ethics approval and consent to participate

The protocol for each study obtained ethical approvals from the Institutional Review Boards (IRBs) of the International Vaccine Institute, the London School of Hygiene and Tropical Medicine, and the Ethics Committee of host country institutions, including KEMRI Scientific and Ethical Review Unit in Kenya, Gabon National Ethics Committee and Institutional Ethics Committee, Scientific Review Board of CERMEL in Gabon, and the IRB of Centre Hospitalier de l'Université de Montréal (CRCHUM) at University of Montreal, and the National Health Ethical Committee of Burkina Faso.

- Consent for publication

Not applicable

- Availability of data and material

Data sharing is not applicable to this article as no datasets were analyzed during the current study.

This manuscript does not include data from the studies described here in. This is a protocol paper. The datasets that are being generated for analysis as described in the current study are not yet publicly available as the studies are currently ongoing at the time of submission. They will be available from the corresponding author on reasonable request.

- Competing interests

The authors declare that they have no competing interests.

- Funding

The current study was supported by funds from the Bill and Melinda Gates Foundation (OPP 1053432). The funding body had no role in the design of the study and collection, analysis, and interpretation of data and in writing the manuscript.

NA receives support from the United Kingdom Medical Research Council (MRC) and Department for International Development (DFID) (MR/K012126/1). VR holds a CIHR-funded Research Chair in Applied Public Health (CPP-137901).

For peer review only - http://bmjopen.bmj.com/site/about/guidelines.xhtml

- Authors' contributions

JKL designed the study, is overseeing data collection, and was a major contributor in writing the manuscript. MC co-designed the study, oversaw some parts of data collection, and supported in writing of the manuscript. JSL was a contributor in designing of the study and oversight of parts of data collection. KSL was a contributor in oversight of data collection. SN supported in data collection. SKL supported in data collection. VR supported in designing of the study and was a major contributor in finalization of the manuscript. JF was a contributor in data collection. BL was a contributor in designing of the study and data collection. SHM was a contributor in designing of the study and site establishment. ME was a contributor in designing of the study. EA supported in data collection. NO supported in data collection. AB supported in data collection. EB supported in data generation. SMN was a contributor in designing of the study and site establishment. STA was a contributor in designing of the study and site establishment. SY was a contributor in designing of the study and site establishment. NA was a major contributor in providing oversight of the data collection and finalization of the manuscript. IKY was a major contributor in designing of the study and finalization of the manuscript.
All authors read and approved the final manuscript.

- Acknowledgements

We thank the doctors and laboratory staff of CSPS of Ouagadougou, ASH, Ganjoni dispensary, Tudor sub-county Hospital, and Coast Provincial General Hospital, as well as at Centre Muraz, CERMEL, and KEMRI. We thank collaborators at AGIR, IRD France, and University of Montreal, and University of Tubingen. Lastly, we would like to thank the DVI team and statisticians and administrative staff at the International Vaccine Institute for their helpful comments during the preparation of this manuscript and support during the studies.

For peer review only

References

1. Bhatt S, Gething PW, Brady OJ, et al. The global distribution and burden of dengue. Nature 2013;**496**(7446):504-7.
2. Murray NE, Quam MB, Wilder-Smith A. Epidemiology of dengue: past, present and future prospects. Clin Epidemiol 2013;**5**:299-309.
3. Chang M, Christophel E, Gopinath D, et al. Challenges and future perspective for dengue vector control in the Western Pacific Region, Regional Analysis. Western Pacific Surveillance and Response Journal 2011;**2**(2):9-16.
4. Eisen L, Beaty B, Morrison A, et al. ProactiveVector control strategies and improved monitoring and evaluation practices for dengue prevention. J Med Entomol 2009;**46**(6):1245-55.
5. Gubler D, Clark G. Dengue/dengue hemorrhagic fever: the emergence of a global health problem. Emerg Infect Dis 1995;**1**(2):55-57. .
6. Amarasinghe A, Kuritsky J, Letson G, et al. Dengue virus infection in Africa. Emerg Infect Dis 2011;**17**(8):1349-54. .
7. Messina J, Brady O, Scott T, et al. Global spread of dengue virus types: mapping the 70 year history. Trends Microbiol 2014;**22**(3):138-46.
8. Fagbami A, Monath T, Fabiyi A. Dengue virus infections in Nigeria: a survey for antibodies in monkeys and humans. Trans R Soc Trop Med Hyg 1977;**71**(1):60-5.
9. Carey D, Causey O, Reddy S, et al. Dengue viruses from febrile patients in Nigeria, 1964-68. Lancet 1971;**1**(7690):105-6.
10. Beatty M, Stone A, Fitzsimons D, et al. Best practices in dengue surveillance: a report from the Asia-Pacific and Americas Dengue Prevention Boards. PLoS Negl Trop Dis 2010;**4**(11):e890.
11. Were F. The dengue situation in Africa. Paediatrics and Internatinoal Child Health 2012;**32**(S1):18-21.
12. Sierra B, Triska P, Soares P, et al. OSBPL10, RXRA and lipid metabolism confer African-ancestry protection against dengue haemorrhagic fever in admixed Cubans. PLoS Pathog 2017;**13**(2):e1006220. .
13. Chacón-Duque J, Adhikari K, Avendaño E, et al. African genetic ancestry is associated with a protective effect on Dengue severity in colombian populations. Infect Genet Evol 2014;**27**:89-95. .
14. Brady OJ, Gething PW, Bhatt S, et al. Refining the global spatial limits of dengue virus transmission by evidence-based consensus. PLoS neglected tropical diseases 2012;**6**(8):e1760.
15. Collenberg E, Ouedraogo T, Ganame J, et al. Seroprevalence of six different viruses among pregnant women and blood donors in rural and urban Burkina Faso: A comparative analysis. J Med Virol 2006;**78**(5):683-92.
16. Ridde V, Agier I, Bonnet E, et al. Presence of three dengue serotypes in Ouagadougou (Burkina Faso): research and public health implications. Infect Dis Poverty 2016;**5**(23).
17. Caron M, Grard G, Paupy C, et al. First evidence of simultaneous circulation of three different dengue virus serotypes in Africa. PloS one 2013;**8**(10):e78030.
18. Leroy EM, Nkoghe D, Ollomo B, et al. Concurrent chikungunya and dengue virus infections during simultaneous outbreaks, Gabon, 2007. Emerging infectious diseases 2009;**15**(4):591-3.
19. Caron M, Paupy C, Grard G, et al. Recent introduction and rapid dissemination of Chikungunya virus and Dengue virus serotype 2 associated with human and mosquito

For peer review only

coinfections in Gabon, central Africa. Clinical infectious diseases : an official publication of the Infectious Diseases Society of America 2012;**55**(6):e45-53.
20. Gabor J, Schwarz N, Esen M, et al. Dengue and chikungunya seroprevalence in Gabonese infants prior to major outbreaks in 2007 and 2010: A sero-epidemiological study. Travel Med Infect Dis 2016.
21. Pourrut X, Nkoghe D, Gonzalez JP, et al. No evidence of dengue virus circulation in rural Gabon. Emerging infectious diseases 2011;**17**(8):1568-9.
22. Andries A, Duong V, Ngan C, et al. Field evaluation and impact on clinical management of a rapid diagnostic kit that detects dengue NS1, IgM and IgG. PLoS Negl Trop Dis 2012;**6**(12):e1993.
23. Lell B, Agnandji S, von Glasenapp I, et al. A randomized trial assessing the safety and immunogenicity of AS01 and AS02 adjuvanted RTS,S malaria vaccine candidates in children in Gabon. PLoS One 2009;**4**(10):e7611. .
24. Agnandji ST, Lell B, Soulanoudjingar SS, et al. First results of phase 3 trial of RTS,S/AS01 malaria vaccine in African children. N Engl J Med 2011;**365**(20):1863-75. .
25. Ochieng C AP, Vittor AY, Nyoka R, Gikunju S, Wachira C, Waiboci L, Umuro M, Kim AA, Nderitu L, Juma B, Montgomery JM, Breiman RF, Fields B. Seroprevalence of Infections with Dengue, Rift Valley Fever and Chikungunya Viruses in Kenya, 2007. PLoS One 2015;**10**(7).
26. Ellis EM, Neatherlin JC, Delorey M, et al. A household serosurvey to estimate the magnitude of a dengue outbreak in Mombasa, Kenya, 2013. PLoS Negl Trop Dis 2015;**9**(4):e0003733.
27. United Nations Development Programme. Population, urban (% of population). In: Reports HD, ed.: UNDP, 2013.
28. United Nations. World Urbanization Prospects: The 2014 Revision, Highlights. In: Department of Economic and Social Affairs PD, ed. United Nations: United Nations, 2014.
29. Central Intelligence Agency. Age structure. The world factbook Central Intelligence Agency.
30. Ramharter M, Adegnika AA, Agnandji ST, et al. History and perspectives of medical research at the Albert Schweitzer Hospital in Lambaréné, Gabon. The Middle European Journal of Medicine, WIENER KLINISCHE WOCHENSCHRIFT 2007;**119**(Suppl 3):8–12.
31. Schwarz NG, Gysels M, Pell C, et al. Reasons for non-adherence to vaccination at mother and child care clinics (MCCs) in Lambaréné, Gabon. Vaccine 2009;**27**(2009):5371-75.
32. Kenya. Secondary Kenya. https://en.wikipedia.org/w/index.php?title=Kenya&oldid=737329303
33. Wichmann O, Yoon I, Vong S, et al. Dengue in Thailand and Cambodia: An assessment of the degree of underestimated disease burden based on reported cases. . PLoS NTD 2010.
34. Gibbons C, Mangen M, Plass D, et al. Measuring underreporting and under-ascertainment in infectious disease datasets: a comparison of methods. BMC public health 2014;**14**(147).
35. European Centre for Disease Prevention and Control (ECDC). Data quality monitoring and surveillance system evaluation - A handbook of methods and applications. Stockholm: European Centre for Disease Prevention and Control (ECDC), 2014.
36. Mease L, Coldren R, Musila L, et al. Seroprevalence and distribution of arboviral infections among rural Kenyan adults: a cross-sectional study. Virol J 2011;**8**(371).

For peer review only - http://bmjopen.bmj.com/site/about/guidelines.xhtml

37. World Health Organization. Guidelines for the clinical evaluation of dengue vaccines in endemic areas. Geneva: World Health Organization, 2008.
38. Alm E, Lindegren G, Falk K, et al. One-step real-time RT-PCR assays for serotyping dengue virus in clinical samples. BMC Infect Dis 2015;**15**(493).
39. Peeling R, Artsob H, Pelegrino J, et al. Evaluation of diagnostic tests: dengue. Nature Reviews Microbiology 2010:S30-S37
40. Kraus A, Messer W, Haymore L, et al. Comparison of plaque- and flow cytometry-based methods for measuring dengue virus neutralization. J Clin Microbiol 2007;**45**(11):3777-80.
41. de Alwis R, de Silva A. Measuring antibody neutralization of dengue virus (DENV) using a flow cytometry-based technique. Methods Mol Biol 2014;**1138**:27-39.
42. Lambeth C, White L, Johnston R, et al. Flow cytometry-based assay for titrating dengue virus. J Clin Microbiol 2005;**43**(7):3267-72.
43. Cabral-Castro M, Saramago Peralta R, Cavalcanti M, et al. A Luminex-based single DNA fragment amplification assay as a practical tool for detecting and serotyping dengue virus. Journal of virological methods 2016;**236**:18-24.
44. Balasuriya U, Shi P, Wong S, et al. Detection of antibodies to West Nile virus in equine sera using microsphere immunoassay. J Vet Diagn Invest 2006;**18**(4):392-5.
45. Beck C, Desprès P, Paulous S, et al. A High-Performance Multiplex Immunoassay for Serodiagnosis of Flavivirus-Associated Neurological Diseases in Horses. Biomed Res Int 2015;**2015**(678084).
46. Hadinegoro SRS. The revised WHO dengue case classification: does the system need to be modified? Paediatr Int Child Health 2012;**32**(S1):33-38.
47. Guilarde AO, Turchi MD, Siqueira Jr JB, et al. Dengue and Dengue Hemorrhagic Fever among Adults: Clinical Outcomes Related to Viremia, Serotypes, and Antibody Response. The Journal of Infectious Diseases;**197**(6): 817-24. .
48. Mossel E, Crabtree M, Mutebi J, et al. Arboviruses Isolated From Mosquitoes Collected in Uganda, 2008-2012. J Med Entomol 2017;**54**(5):1403-09. .
49. Shope R, al. e. *The Flaviviruses: Detection, Diagnosis and Vaccine Development*: Academic Press, 2003.
50. Nikolay B, Diallo M, Boye C, et al. Usutu virus in Africa. Vector Borne Zoonotic Dis 2011;**11**(11):1417-23.

For peer review only - http://bmjopen.bmj.com/site/about/guidelines.xhtml

Fig. 1 Description of the study components, including passive facility-based fever surveillance, healthcare utilization surveys, cost-of-illness surveys, and serological surveys

There are two arms in the study package, composed of four parts. In the health facility-based arm of the study package, there are the passive facility-based fever surveillance and cost-of-illness survey embedded within the surveillance. In the community arm of the study, there are serological survey and healthcare utilization survey.

For peer review only

For peer review only - http://bmjopen.bmj.com/site/about/guidelines.xhtml

Fig. 2 Map of the study area in Ouagadougou, Burkina Faso

Fig. 3 Map of the study area in Lambaréné, Gabon

Fig. 4 Map of the study area in Mombasa, Kenya

Figures 2 – 4 show the map of the study area at each site in Burkina Faso, Gabon, and Kenya.

For peer review only

For peer review only - http://bmjopen.bmj.com/site/about/guidelines.xhtml

Fig. 5 Patient flow in the fever surveillance

Eligible febrile patients identified and enrolled as study subjects will follow these steps to complete participation in the passive fever surveillance.

For peer review only

Fig. 6 Laboratory testing algorithm for dengue

Samples from subjects of the passive fever surveillance would follow these steps of the testing algorithm for confirmation of dengue.

For peer review only

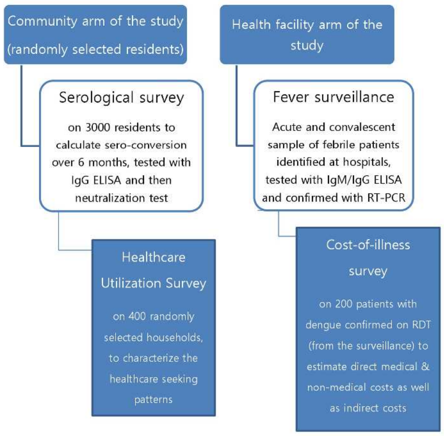

Fig. 1 Description of study components, including passive facility-based fever surveillance, healthcare utilization surveys, cost-of-illness surveys, and serological surveys

210x297mm (300 x 300 DPI)

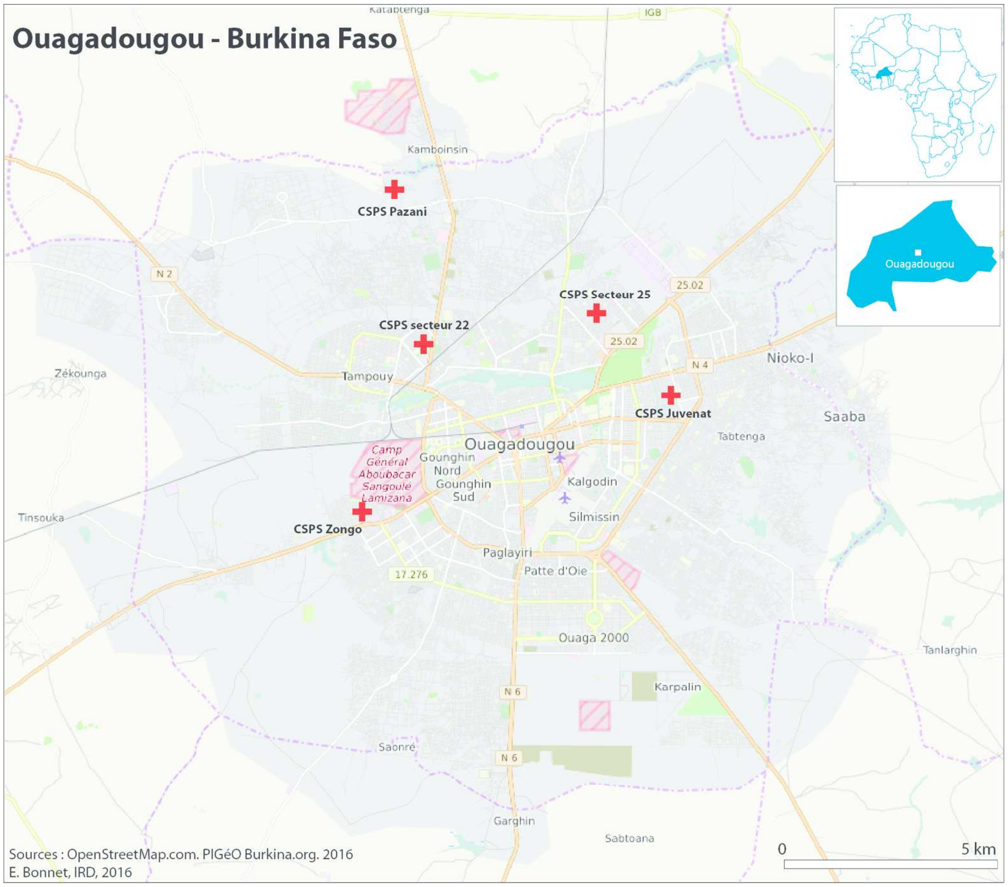

A map of the study area in Ouagadougou, Burkina Faso

165x145mm (300 x 300 DPI)

only

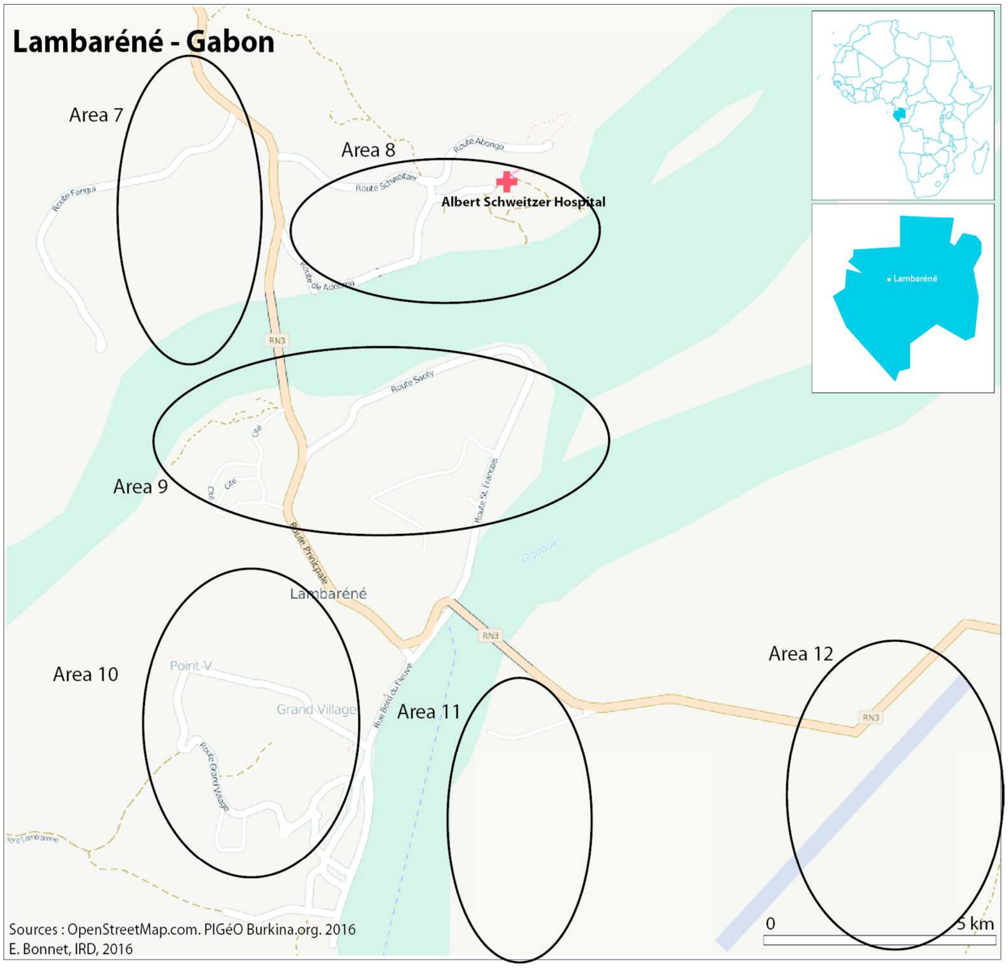

A map of the study area in Lambarene, Gabon

151x145mm (300 x 300 DPI)

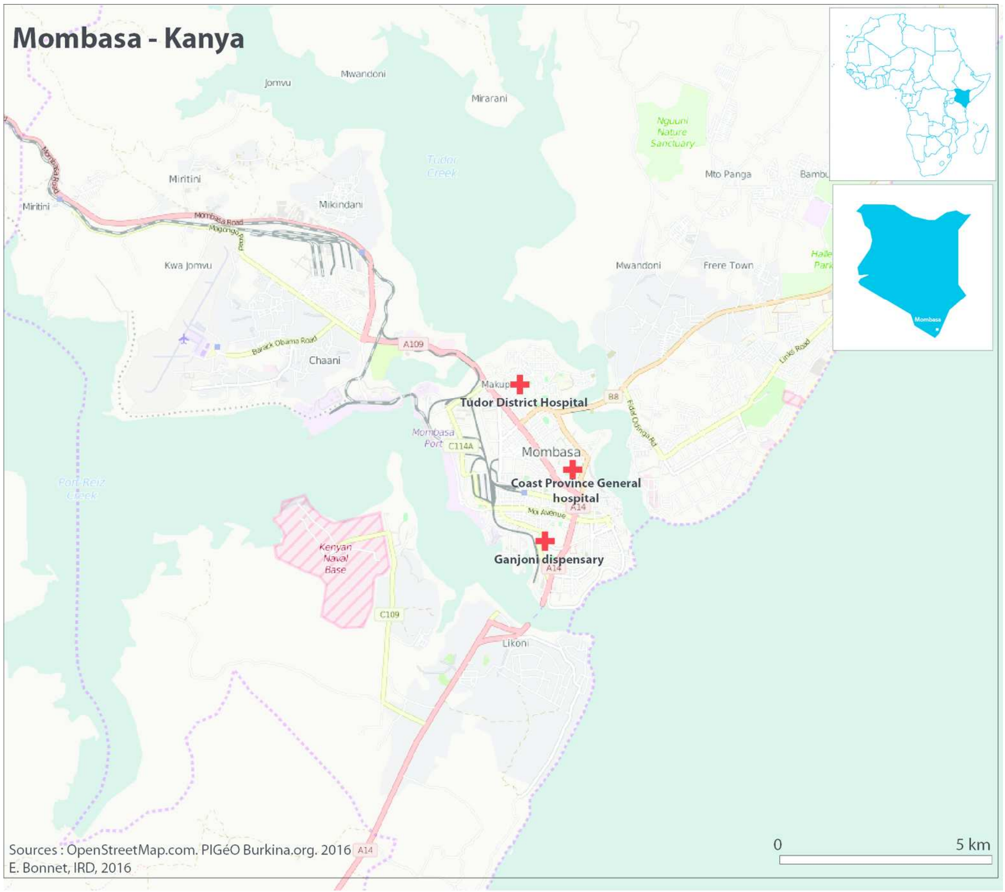

A map of the study area in Mombasa, Kenya

166x147mm (300 x 300 DPI)

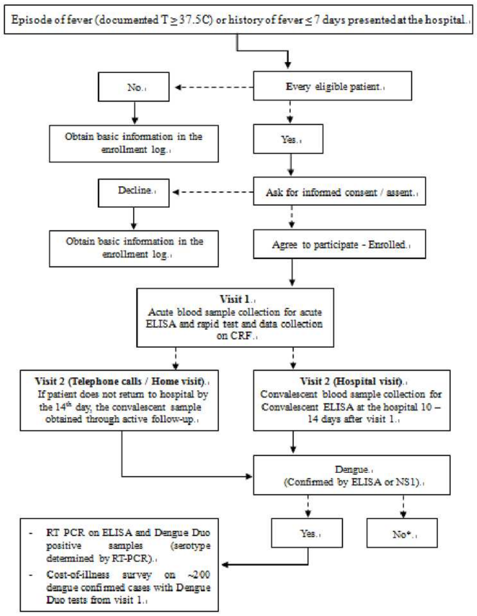

* A small number of those samples that are negative on ELISA or NS1 are tested with PCR to exclude false negative results of the ELISA.

Patient flow in the fever surveillance - Eligible febrile patients identified and enrolled as study subjects will follow these steps to complete participation in the passive fever surveillance.

140x198mm (300 x 300 DPI)

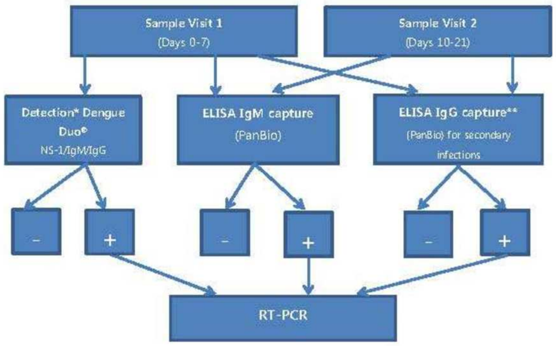

Fig. 6 Laboratory testing algorithm for dengue

* Dengue Duo® test is performed on enrolled febrile patients to identify dengue cases for immediate follow-up of dengue-confirmed cases in the cost-of-illness survey.

**Selected samples, including those that were found positive by IgM and NS1 on Dengue Duo®, as well as those positive by IgM and IgG capture ELISA, will be tested with RT-PCR.

Laboratory testing algorithm for dengue: Samples from subjects of the passive fever surveillance would follow these steps of the testing algorithm for confirmation of dengue.

140x198mm (300 x 300 DPI)
